# Using electronic health records to inform trial feasibility in a rare autoimmune blistering skin disease in England

**DOI:** 10.1186/s12874-021-01212-1

**Published:** 2021-02-04

**Authors:** M. S. M. Persson, K. E. Harman, K. S. Thomas, J. R. Chalmers, Y. Vinogradova, S. M. Langan, J. Hippisley-Cox, S. Gran

**Affiliations:** 1 Centre of Evidence Based Dermatology, School of Medicine, University of Nottingham, Nottingham, UK; 2 Division of Primary Care, University of Nottingham, Nottingham, UK; 3 Department of Non-communicable Disease Epidemiology, London School of Hygiene and Tropical Medicine, London, UK; 4 Nuffield Department of Primary Care Health Sciences, University of Oxford, Oxford, UK

**Keywords:** Bullous pemphigoid, Electronic health records, Clinical practice research Datalink, Oral prednisolone, Hospital episode Statistics, Trial design, Rare disease

## Abstract

**Background:**

Trials of novel agents are required to improve the care of patients with rare diseases, but trial feasibility may be uncertain due to concerns over insufficient patient numbers. We aimed to determine the size of the pool of potential participants in England 2015–2017 for trials in the autoimmune blistering skin disease bullous pemphigoid.

**Methods:**

The size of the pool of potential participants was estimated using routinely collected healthcare data from linked primary care (Clinical Practice Research Datalink; CPRD) and secondary care (Hospital Episode Statistics; HES) databases. Thirteen consultant dermatologists were surveyed to determine the likelihood that a patient would be eligible for a trial based on the presence of cautions or contra-indications to prednisolone use. These criteria were applied to determine how they influenced the potential pool of participants.

**Results:**

Extrapolated to the population of England, we would expect approximately 10,800 (point estimate 10,747; 95% CI 7191 to 17,239) new cases of bullous pemphigoid to be identified in a three-year period. For a future trial involving oral prednisolone (standard care), the application of cautions to its use as exclusion criteria would result in approximately 365 potential participants unlikely to be recruited, a further 5332 could be recruited with caution, and 5104 in whom recruitment is still possible. 11–17% of potential participants may have pre-existing dementia and require an alternative consent process.

**Conclusions:**

Routinely collected electronic health records can be used to inform the feasibility of clinical trials in rare diseases, such as whether recruitment is feasible nationally and how long recruitment might take to meet recruitment targets. Future trials of bullous pemphigoid in England may use the data presented to inform trial design, including eligibility criteria and consent processes for enrolling people with dementia.

**Supplementary Information:**

The online version contains supplementary material available at 10.1186/s12874-021-01212-1.

## Background

Bullous pemphigoid is a rare blistering skin disease that predominantly affects older people [1]. Following a diagnosis of bullous pemphigoid, individuals affected are almost three times more likely to die than their peers [1]. Current treatment regimens are often based on oral prednisolone, which has been the mainstay of treatment for decades. Oral prednisolone is effective in treating blisters, but may lead to the development or exacerbation of potentially life-limiting diseases such as diabetes mellitus and infections [2, 3]. Trials of novel agents are required in order to improve patient management, [4] however, the pool of potentially eligible patients in the UK is currently unknown.

Routinely collected electronic health records (EHR) can be used to inform trial feasibility and guide trial development. Many rare diseases are in need of clinical trials of new treatments, however, it is difficult to proceed with trial planning and design without first knowing if there are enough people to make such a trial feasible. The present work focuses on bullous pemphigoid, but the methodology used may be relevant to a multitude of rare diseases.

Using EHR, we aimed to estimate the number of new cases of bullous pemphigoid over a three-year period in England and to determine how many of these patients were affected by conditions that may exclude them from participating in a trial assuming a control arm treated with 0.5 mg/kg oral prednisolone (considered standard care).

## Methods

### Study design

This was a descriptive study, reported using RECORD guidelines for the reporting of studies conducted using observational routinely collected health data [5].

### Data sources

Two linked data sources were utilised: primary care data from the Clinical Practice Research Datalink (CPRD) GOLD and secondary care data from the Hospital Episode Statistics (HES).

The CPRD GOLD is a longitudinal database that contains the anonymised healthcare records for UK general practices using the Vision software system. Within the CPRD, clinical data from consultations, hospital discharge letters, and specialist clinic letters are recorded using Read codes [6, 7].

Linked data are available from HES admitted patient care for approximately 75% of the English practices within the CPRD, covering approximately 10 million people. Within the HES admitted patient care, diagnoses from each hospitalisation episode are recorded using the International Classification of Diseases version 10 (ICD-10) and procedures are recorded using the Office of Population Censuses and Surveys Classification of Interventions and Procedures (OPCS version 4.6).

### Study population

Adult men and women registered with the 410 HES-linked general practices during the period of 1 January 2015 and 31 December 2017 formed the study population for the examination of the incidence rate of bullous pemphigoid. Only patients whose record was verified as “acceptable” research quality were included. To ensure higher validity of the included patient records, standard data quality checks are conducted to identify and exclude “unacceptable” patients with non-continuous follow up (i.e., not permanently registered with the practice) or poor data recording [7].

For the application of trial exclusion criteria, a subset of the population above was used. Here it was limited to those with an incident diagnosis of bullous pemphigoid between 1 January 2015 and 31 December 2017. Incident diagnoses were those with a bullous pemphigoid index date at least one year after their current registration date with their GP. The one-year lag period was implemented in order to minimise the risk of prevalent cases being identified as incident cases [8].

A diagnosis of bullous pemphigoid is generally made in dermatology clinics, following clinical examination and laboratory investigations, (e.g., skin biopsy for histology and direct immunofluorescence). The diagnosis is subsequently communicated to primary care, where it is usually entered in the CPRD using Read codes, or to the person’s inpatient hospital records, where it is entered in HES admitted patient care using ICD-10 codes. Patients with a Read code for “bullous pemphigoid” (M145), “pemphigoid “(M145.00), “pemphigoid not otherwise specified” (M145z00) or an ICD-10 code for “bullous pemphigoid” (L12.0) or “pemphigoid, unspecified” (L12.9) were identified using a validated clinical-code based algorithm [9].

### Observation period

The three-year study included two observation periods: one observation period for the calculation of the incidence rate and one for the application of trial exclusion criteria to the population of incident cases, described below.

For the incidence calculation, the observation period commenced on the latest of (i) 1 January 2015, (ii) 12 months after the date the patient registered with their current practice, (iii) the date the patient’s practice was declared up-to-standard (i.e., date assigned on completion of regular audits confirming data quality), or (iv) the patient’s 18th birthday. The observation period terminated on the earliest of (i) 31 December 2017, (ii) the date of death, (iii) the date the patient left the practice, (iv) the practice’s last data collection date, or (v) the most recent linkage date between the CPRD and HES.

When applying the trial exclusion criteria to incident cases, only data prior to the bullous pemphigoid index date were examined. Therefore the observation period commenced on the practice’s up-to-standard date and terminated on the index date for bullous pemphigoid.

### Definitions of exclusion criteria

Contra-indications or cautions to oral prednisolone use were identified from those listed in the British National Formulary (BNF, 2020) [3]. A total of 2 contra-indications and 19 cautions were identified. An additional caution, malignancy (excluding basal cell carcinoma, BCC), was added to the list as it is an exclusion criteria that has previously been implemented in clinical trials of oral corticosteroid in bullous pemphigoid [10, 11]. Based on the guidance in the BNF and clinical expertise, each contraindication was coupled to a specified timeframe of relevance, relative to the bullous pemphigoid index date (Table 1). For example, receiving a varicella zoster vaccine was only a contra-indication to oral prednisolone use if it occurred in the three months preceding the bullous pemphigoid index date. Conversely, osteoporosis was considered a caution if it occurred any time before the bullous pemphigoid date.

**Table 1:** Conditions listed as cautions to the use of oral corticosteroids in the British National Formulary and the number (proportion) of incident cases of bullous pemphigoid with a record for each caution within specified timelines. Presented alongside the recruitment status associated with having that condition when considering a clinical trial involving 0.5 mg/kg oral prednisolone

Caution	Timeline*	N (%) of cases	Recruitment status**
Total incident cases		237	
Diabetes mellitus	Any time before	55 (23.2%)	Recruit with caution
Diverticulitis	In 1 year before	0	Possible
Epilepsy	Any time before	12 (5.1%)	Possible
Glaucoma	Any time before	16 (6.8%)	Recruit with caution
Heart failure	Any time before	43 (18.1%)	Recruit with caution
Hypertension	Any time before	159 (67.1%)	Possible
Hypothyroidism	Any time before	30 (12.7%)	Possible
Intestinal anastomosis	In 6 months before	1 (0.4%)	Unlikely
Malignancy (excluding BCC)	In 5 years before	23 (9.7%)	Possible
Myasthenia gravis	Any time before	1 (0.4%)	Possible
Myocardial infarction	In 6 months before	3 (1.3%)	Recruit with caution
Myopathy	Any time before	1 (0.4%)	Recruit with caution
Ocular herpes simplex	Any time before	1 (0.4%)	Unlikely
Osteoporosis	Any time before	38 (16.0%)	Recruit with caution
Peptic ulcer	In 1 year before	2 (0.8%)	Recruit with caution
Septicaemia/sepsis	In 3 months before	3 (1.3%)	Unlikely
Severe mental illness	In 10 years before	1 (0.4%)	Recruit with caution
Systemic sclerosis	Any time before	0	Possible
Venous thromboembolic disorders	Any time before	19 (8.0%)	Possible
Tuberculosis	Any time before	3 (1.3%)	Unlikely
Ulcerative colitis	In 5 years before	2 (0.8%)	Possible
Varicella zoster vaccine	In 3 months before	4 (1.7%)	Recruit with caution

* Timeline relative to bullous pemphigoid index date

** Recruitment status was determined by the category chosen by > 50% of the consultant dermatologists in the online survey: Possible (> 50% classed the caution unlikely to lead to exclusion), recruit with caution (> 50% classed the caution very or somewhat likely to lead to exclusion, note that < 50% considered it very likely), or unlikely (> 50% classed the caution as very likely to lead to exclusion)

Using an online survey (Microsoft Office Forms), 13 consultant dermatologists experienced in managing bullous pemphigoid graded whether they were “very likely”, “somewhat likely”, or “unlikely” to exclude a person from a trial involving oral prednisolone if they had a history of any of the 22 conditions within the specified time frames. The dermatologists were all UK-based and identified based on their involvement with the British Association of Dermatologists guidelines for the management of pemphigus vulgaris and bullous pemphigoid or the Pemphigoid and Pemphigus Priority Setting Partnership. Responses were pooled and where there were discrepancies, the caution was classified in accordance with the category chosen by > 50% of dermatologists. This was felt to be a reasonable cut-off for determining consensus.

### Code lists for exclusion criteria and dementia

Code lists were developed for the 22 cautions/contraindications considered potential exclusion criteria for a trial involving oral prednisolone and for dementia. Since bullous pemphigoid generally affects older people, where the prevalence of dementia is increased, [12] it was felt important to identify pre-existing dementia, as well as prednisolone cautions, to provide an indication of the proportion of patients with capacity to consent.

Primary care and secondary care code lists were developed for each caution based on published Read and ICD-10 lists, respectively. The code lists were primarily drawn from the work of Kuan et al., [12] a study of 308 physical and mental health conditions, as this allowed a consistent approach to be applied across all conditions, and were modified and supplemented as required. For example, only codes from the CALIBER “Thyroid disease” category that indicated decreased thyroid function were used for hypothyroidism in the current work. Additionally, the code lists for cautions coupled with a limited time frame (e.g., six months before bullous pemphigoid) were manually reviewed and codes stating “history of” or similar were excluded.

The code lists were supplemented with Read, ICD-10, and OPCS codes generated through manual searching of the data dictionaries. Finally, varicella zoster code lists involving product and vaccination codes were drawn from the work of Jain et al., [13]. Code lists were reviewed in conjunction with a consultant dermatologist (KEH) to ensure suitability and clinical relevance. Full code lists are available in the Additional File 1.

### Identification of exclusion criteria and dementia

The code lists were applied to the CPRD clinical, referral, test, therapy, and immunisation data and HES admitted patient care and OPCS data. For cautions with a time frame of “any time before bullous pemphigoid”, all codes prior to the bullous pemphigoid index date were identified. For conditions with a limited time frame (e.g., in the “x months” before bullous pemphigoid) only codes that occurred in the “x months” before bullous pemphigoid, but at least 12 months after the patient’s current registration date were examined. The time limit of 12 months after the patient’s current registration date was used to exclude potential historic events entered on transfer to a new general practice.

### Statistical analysis

The incidence rate of bullous pemphigoid was determined per 100,000 person-years using previously published methods [1]. Age-specific incidence rates were calculated for five-year age bands. These were applied to the Office for National Statistics population estimate for England [14] over the same time period to estimate the total number of new cases that might be expected in England.

Of the incident cases, the number and proportion with a record for each of the cautions within the specified time frame was determined. The proportion of patients with conditions that were unlikely to allow recruitment (recruitment unlikely group), that would be possible to recruit with caution (recruit with caution group), and that would be possible to recruit (recruitment possible group) were determined. For each category, the proportion with pre-existing dementia were determined. The proportions were applied to the estimated number of new cases in England 2015–2017.

Analyses were conducted with Stata 16 (2019; StataCorp LLC, College Station, TX, USA).

### Ethical approval

The present study was approved by the Independent Scientific Advisory Committee for the CPRD (ISAC protocol no 18_224).

## Results

### Classification of recruitment status from survey

Of the 22 conditions considered to be contra-indications or cautions, four were categorised as recruitment unlikely (> 50% of dermatologists considered the disease “very likely” to lead to exclusion), nine were recruit with caution (> 50% of dermatologists considered the condition “very likely” or “somewhat likely” to lead to exclusion; but < 50% of dermatologists considered the disease “very likely”), and nine were recruitment possible (> 50% of dermatologists considered the condition “unlikely” to lead to exclusion).

### Study population

Between 2015 and 2017, 237 incident cases of bullous pemphigoid were identified in the CPRD and HES (Additional File 2). The median age when the diagnosis of bullous pemphigoid was first recorded was 78.7 years (IQR 70.9 to 87.2). 134 (56.5%) of those affected were women.

### Incidence of bullous pemphigoid 2015–2017

The incidence of bullous pemphigoid was 8.4 (95%CI 7.4 to 9.5) per 100,000 person-years. The age-specific incidence rates of bullous pemphigoid per age category are available in the Additional File 3. Extrapolated to the population of England there would be an estimated 10,800 (point estimate 10,747; 95% CI 7191 to 17,239) new cases in England in a three-year period.

### Cautions to oral prednisolone use

Of the incident cases, 44 (18.6%) had no recorded cautions to the use of oral prednisolone. Conversely, 193 (81.4%) had a record for at least one caution to oral prednisolone use. The commonest comorbidities considered cautions to the use of prednisolone and present at the time the diagnosis of bullous pemphigoid was first recorded were hypertension (67.1%), diabetes mellitus (23.2%), heart failure (18.1%), and osteoporosis (16.0%) (Table 1).

### Classification of population by recruitment status

Across the incident cases, 112 (47.3%) patients were considered possible to recruit (no caution or only cautions unlikely to lead to exclusion), 117 (49.4%) could be recruited with caution, and 8 (3.4%) would be unlikely to be recruited to a trial of oral prednisolone. Extrapolated to the population of England in 2015 to 2017, approximately 5104 would be possible to recruit and an additional 5332 could be recruited with caution. However, 365 of incident cases would be unlikely to be recruited due to cautions to the use of oral prednisolone. Of these, 911 (17.9%), 592 (11.1%), and 46 (12.5%), respectively, may have pre-existing dementia (Table 2).

**Table 2:** Number (proportion) of the incident cases of bullous pemphigoid with and without pre-existing dementia according to their recruitment status

Recruitment status	*N*	With dementia	Without dementia
All patients	237	34 (14.4%)	203 (85.7%)
Recruitment possible	112	20 (17.9%)	92 (82.1%)
Recruit with caution	117	13 (11.1%)	104 (88.9%)
Recruitment unlikely	8	1 (12.5%)	7 (87.5%)

## Discussion

We have described a novel use of EHRs, such as the CPRD and HES, for the planning of clinical trials in rare diseases. Although this approach has been on the agenda for years, few publications incorporate this process in trial planning. We have shown that data from EHRs may be useful for determining whether conducting a trial for a rare disease is feasible nationally, how many practices might need to be approached, and how long recruitment might take to meet recruitment targets. In addition, linked EHR data can be used to determine how the pool of eligible participants changes according to how trial eligibility criteria are implemented.

We suggest the following considerations when using EHR to plan the design and conduct of a trial. Firstly, it should be possible to reliably identify the disease of interest from the EHR available. Such validation work is essential to ensure that the work captures patients with the disease of interest. Secondly, consideration needs to be paid to the comorbidities examined. We chose a broad approach that might be useful for a variety of new treatments, assuming the trials use oral prednisolone as the comparator. In the future, researchers may choose to use criteria that relate specifically to their research question, treatments of interest, and sample size requirements. Finally, the benefits of conducting such a study may be beneficial also during the conduct of a trial. The characteristics of the population of potentially eligible participants identified via EHR can be compared with current recruitment patterns to determine the generalisability of the study population and identify areas for improvement in the recruitment strategy.

For bullous pemphigoid, we have shown that pre-existing comorbidities that are considered cautions to oral prednisolone use are common at the time of diagnosis of bullous pemphigoid. As many as 81.4% of patients have at least one caution that may make them ineligible for a clinical trial if all cautions were considered as exclusion criteria. This highlights that the balance between the safety of included patients and the impact on the generalisability of trial results needs to be considered. Finally, we have shown that trial planning should consider patients with dementia, as about one in six patients may have pre-existing dementia.

In keeping with the literature, cardiovascular comorbidities, and hypertension in particular, are common amongst people with bullous pemphigoid. Indeed, comorbidities in general were common amongst the population, which is reflective of the fact that bullous pemphigoid most often affects older people [1]. Previous work has shown that conditions such as hypertension, malignancies, diverticular disease, heart failure, and mental illnesses are common amongst the older population that is also most susceptible to bullous pemphigoid. However, not all comorbidities make a patient ineligible. In a recent clinical trial involving oral prednisolone in patients with bullous pemphigoid, approximately 9% of individuals assessed for eligibility were excluded on the grounds of “substantial comorbidities”. This significant proportion suggests that in practice, clinicians assessing patients for eligibility to a clinical trial may choose to exclude not only the 3.4% we deemed ineligible, but also a proportion of those we considered could be recruited with caution.

A limitation of our work is the inability to determine the severity of either the bullous pemphigoid or the caution, which are vital for clinical decision-making regarding trial eligibility. Furthermore, we have not been able to ascertain whether or not the cautions identified from the CPRD and HES relate to therapy. It is possible that oral prednisolone therapy was commenced in specialist dermatology clinics prior to the recording of bullous pemphigoid in the CPRD or HES and a small proportion of the cautions observed, such as diabetes and osteoporosis, may be a consequence of the initial therapy. Additionally, although we used both primary (CPRD) and secondary care (HES) records to identify comorbidities, the true number may be underestimated (resulting in a misclassification bias) if patients have not sought medical treatment for their condition or if the information has not been recorded. However, we believe this number is likely to be small and largely limited to conditions with minor symptoms. The 13 consultant dermatologists whose views shaped the classification of cautions were chosen based on their experience in the area, but may not be representative of all dermatologists that may be involved in trial recruitment in the UK. Also, the data drawn were from England and may not be representative of other populations. Finally, our findings are based on a relatively small number of patients identified in the CPRD and HES over the most recent three-year period for which the data were available. Expanding the observation period would have increased the numbers, but was felt to reduce the applicability of findings to future trials as disease recording and patient characteristics may have changed over time.

## Conclusions

In conclusion, we have used EHR to guide the design and implementation of a clinical trial in bullous pemphigoid. We have provided an estimation of the number of people that may be available to recruit to a trial involving oral prednisolone. Researchers may use our findings to determine the feasibility of conducting a clinical trial of bullous pemphigoid in England based on their specific sample size parameters. We suggest, however, that recruitment is likely to be challenging and eligibility criteria should be broad to ensure generalisable results.

## Supplementary Information

**Additional file 1:.** Code lists used for the 22 cautions to oral prednisolone use

**Additional file 2:.** Identification of incident cases of bullous pemphigoid in HES-linked CPRD practices and HES admitted patient care in England 2015–2017

**Additional file 3:.** Incidence rate of bullous pemphigoid per 100,000 person-years in England 2015–2017 by age category

## Data Availability

The data that support the findings of this study are available from the CPRD (www.cprd.com) but restrictions apply to the availability of these data, which were used under license for the current study, and so are not publicly available. Data are however available from the authors upon reasonable request and with permission of the CPRD.

## Abbreviations

CPRD: Clinical Practice Research Datalink
EHR: Electronic health records
HES: Hospital Episode Statistics
ICD: International Classification of Disease
OPCS: Office of Population Census Surveys
BNF: British National Formulary
