# Supplementary material for: Using electronic health records to inform trial feasibility in a rare autoimmune blistering skin disease in England
Source: BMC Med Res Methodol. 2021 Feb 4;21:22. doi: 10.1186/s12874-021-01212-1 (PMC7863423; doi:10.1186/s12874-021-01212-1)
Supplement: Supplementary file 1 — Additional file 1:. Code lists used for the 22 cautions to oral prednisolone use [file 12874_2021_1212_MOESM1_ESM.pdf]

Code lists for cautions

Code type	Code	Description	Disease	Source
Read	E112300	Single major depressive episode, severe, without psychosis	Severe mental illness	CALIBER
Read	E112400	Single major depressive episode, severe, with psychosis	Severe mental illness	CALIBER
Read	E113300	Recurrent major depressive episodes, severe, no psychosis	Severe mental illness	CALIBER
Read	E113400	Recurrent major depressive episodes, severe, with psychosis	Severe mental illness	CALIBER
Read	Eu32200	[X]Severe depressive episode without psychotic symptoms	Severe mental illness	CALIBER
Read	Eu32300	[X]Severe depressive episode with psychotic symptoms	Severe mental illness	CALIBER
Read	Eu32600	[X]Major depression, moderately severe	Severe mental illness	CALIBER
Read	Eu32700	[X]Major depression, severe without psychotic symptoms	Severe mental illness	CALIBER
Read	Eu32800	[X]Major depression, severe with psychotic symptoms	Severe mental illness	CALIBER
Read	Eu32900	[X]Single major depr ep, severe with psych, psych in remiss	Severe mental illness	CALIBER
Read	Eu32A00	[X]Recurr major depr ep, severe with psych, psych in remiss	Severe mental illness	CALIBER
Read	Eu33200	[X]Recurr depress disorder cur epi severe without psyc sympt	Severe mental illness	CALIBER
Read	Eu33300	[X]Recurrent depress disorder cur epi severe with psyc symp	Severe mental illness	CALIBER
Read	Eu33313	[X]Recurr severe episodes/major depression+psychotic symptom	Severe mental illness	CALIBER
Read	Eu33314	[X]Recurr severe episodes/psychogenic depressive psychosis	Severe mental illness	CALIBER
Read	Eu33315	[X]Recurrent severe episodes of psychotic depression	Severe mental illness	CALIBER
Read	Eu33316	[X]Recurrent severe episodes/reactive depressive psychosis	Severe mental illness	CALIBER
Read	1461	H/O: dementia	Dementia	CALIBER
Read	66h..00	Dementia monitoring	Dementia	CALIBER
Read	6AB..00	Dementia annual review	Dementia	CALIBER
Read	8CMZ.00	Dementia care plan	Dementia	CALIBER
Read	9hD0.00	Excepted from dementia quality indicators: Patient unsuitabl	Dementia	CALIBER
Read	9hD1.00	Excepted from dementia quality indicators: Informed dissent	Dementia	CALIBER
Read	9hD..00	Exception reporting: dementia quality indicators	Dementia	CALIBER
Read	9Ou1.00	Dementia monitoring first letter	Dementia	CALIBER

Read	9Ou2.00	Dementia monitoring second letter	Dementia	CALIBER
Read	9Ou3.00	Dementia monitoring third letter	Dementia	CALIBER
Read	9Ou4.00	Dementia monitoring verbal invite	Dementia	CALIBER
Read	9Ou5.00	Dementia monitoring telephone invite	Dementia	CALIBER
Read	9Ou..00	Dementia monitoring administration	Dementia	CALIBER
Read	E000.00	Uncomplicated senile dementia	Dementia	CALIBER
Read	E001000	Uncomplicated presenile dementia	Dementia	CALIBER
Read	E001100	Presenile dementia with delirium	Dementia	CALIBER
Read	E001200	Presenile dementia with paranoia	Dementia	CALIBER
Read	E001300	Presenile dementia with depression	Dementia	CALIBER
Read	E001.00	Presenile dementia	Dementia	CALIBER
Read	E001z00	Presenile dementia NOS	Dementia	CALIBER
Read	E002000	Senile dementia with paranoia	Dementia	CALIBER
Read	E002100	Senile dementia with depression	Dementia	CALIBER
Read	E002.00	Senile dementia with depressive or paranoid features	Dementia	CALIBER
Read	E002z00	Senile dementia with depressive or paranoid features NOS	Dementia	CALIBER
Read	E003.00	Senile dementia with delirium	Dementia	CALIBER
Read	E004000	Uncomplicated arteriosclerotic dementia	Dementia	CALIBER
Read	E004100	Arteriosclerotic dementia with delirium	Dementia	CALIBER
Read	E004200	Arteriosclerotic dementia with paranoia	Dementia	CALIBER
Read	E004300	Arteriosclerotic dementia with depression	Dementia	CALIBER
Read	E004.00	Arteriosclerotic dementia	Dementia	CALIBER
Read	E004.11	Multi infarct dementia	Dementia	CALIBER
Read	E004z00	Arteriosclerotic dementia NOS	Dementia	CALIBER
Read	E00..00	Senile and presenile organic psychotic conditions	Dementia	CALIBER
Read	E00..11	Senile dementia	Dementia	CALIBER
Read	E00..12	Senile/presenile dementia	Dementia	CALIBER
Read	E00y.00	Other senile and presenile organic psychoses	Dementia	CALIBER
Read	E00y.11	Presbyophrenic psychosis	Dementia	CALIBER
Read	E00z.00	Senile or presenile psychoses NOS	Dementia	CALIBER
Read	E041.00	Dementia in conditions EC	Dementia	CALIBER
Read	Eu00000	[X]Dementia in Alzheimer's disease with early onset	Dementia	CALIBER
Read	Eu00011	[X]Presenile dementia,Alzheimer's type	Dementia	CALIBER
Read	Eu00012	[X]Primary degen dementia, Alzheimer's type, presenile onset	Dementia	CALIBER
Read	Eu00013	[X]Alzheimer's disease type 2	Dementia	CALIBER
Read	Eu00100	[X]Dementia in Alzheimer's disease with late onset	Dementia	CALIBER
Read	Eu00111	[X]Alzheimer's disease type 1	Dementia	CALIBER
Read	Eu00112	[X]Senile dementia,Alzheimer's type	Dementia	CALIBER
Read	Eu00113	[X]Primary degen dementia of Alzheimer's type, senile onset	Dementia	CALIBER
Read	Eu00200	[X]Dementia in Alzheimer's dis, atypical or mixed type	Dementia	CALIBER

Read	Eu00.00	[X]Dementia in Alzheimer's disease	Dementia	CALIBER
Read	Eu00z00	[X]Dementia in Alzheimer's disease, unspecified	Dementia	CALIBER
Read	Eu00z11	[X]Alzheimer's dementia unspec	Dementia	CALIBER
Read	Eu01000	[X]Vascular dementia of acute onset	Dementia	CALIBER
Read	Eu01100	[X]Multi-infarct dementia	Dementia	CALIBER
Read	Eu01111	[X]Predominantly cortical dementia	Dementia	CALIBER
Read	Eu01200	[X]Subcortical vascular dementia	Dementia	CALIBER
Read	Eu01300	[X]Mixed cortical and subcortical vascular dementia	Dementia	CALIBER
Read	Eu01.00	[X]Vascular dementia	Dementia	CALIBER
Read	Eu01.11	[X]Arteriosclerotic dementia	Dementia	CALIBER
Read	Eu01y00	[X]Other vascular dementia	Dementia	CALIBER
Read	Eu01z00	[X]Vascular dementia, unspecified	Dementia	CALIBER
Read	Eu02z00	[X] Unspecified dementia	Dementia	CALIBER
Read	Eu02z11	[X] Presenile dementia NOS	Dementia	CALIBER
Read	Eu02z12	[X] Presenile psychosis NOS	Dementia	CALIBER
Read	Eu02z13	[X] Primary degenerative dementia NOS	Dementia	CALIBER
Read	Eu02z14	[X] Senile dementia NOS	Dementia	CALIBER
Read	Eu02z15	[X] Senile psychosis NOS	Dementia	CALIBER
Read	Eu02z16	[X] Senile dementia, depressed or paranoid type	Dementia	CALIBER
Read	Eu04100	[X]Delirium superimposed on dementia	Dementia	CALIBER
Read	F110000	Alzheimer's disease with early onset	Dementia	CALIBER
Read	F110100	Alzheimer's disease with late onset	Dementia	CALIBER
Read	F110.00	Alzheimer's disease	Dementia	CALIBER
Read	Fyu3000	[X]Other Alzheimer's disease	Dementia	CALIBER
Read	ZS7C500	Language disorder of dementia	Dementia	CALIBER
ICD10	F00	Dementia in Alzheimer's disease	Dementia	CALIBER
ICD10	F01	Vascular dementia	Dementia	CALIBER
ICD10	F03	Unspecified dementia	Dementia	CALIBER
ICD10	F05.1	Delirium superimposed on dementia	Dementia	CALIBER
ICD10	G30	Alzheimer's disease	Dementia	CALIBER
Read	C100112	Non-insulin dependent diabetes mellitus	Diabetes mellitus	CALIBER
Read	C10..00	Diabetes mellitus	Diabetes mellitus	CALIBER
Read	C10F.00	Type 2 diabetes mellitus	Diabetes mellitus	CALIBER
Read	C10F.00	Type 2 diabetes mellitus	Diabetes mellitus	CALIBER
Read	C100011	Insulin dependent diabetes mellitus	Diabetes mellitus	CALIBER
Read	F420.00	Diabetic retinopathy	Diabetes mellitus	CALIBER
Read	C10FJ00	Insulin treated Type 2 diabetes mellitus	Diabetes mellitus	CALIBER
Read	C10FJ00	Insulin treated Type 2 diabetes mellitus	Diabetes mellitus	CALIBER

Read	C10E.00	Type 1 diabetes mellitus	Diabetes mellitus	CALIBER
Read	C10E.00	Type 1 diabetes mellitus	Diabetes mellitus	CALIBER
Read	C108.00	Insulin dependent diabetes mellitus	Diabetes mellitus	CALIBER
Read	C101.00	Diabetes mellitus with ketoacidosis	Diabetes mellitus	CALIBER
Read	66A4.00	Diabetic on oral treatment	Diabetes mellitus	CALIBER
Read	F381311	Diabetic amyotrophy	Diabetes mellitus	CALIBER
Read	F372.12	Diabetic neuropathy	Diabetes mellitus	CALIBER
Read	66AJ.00	Diabetic - poor control	Diabetes mellitus	CALIBER
Read	K01x100	Nephrotic syndrome in diabetes mellitus	Diabetes mellitus	CALIBER
Read	C104.11	Diabetic nephropathy	Diabetes mellitus	CALIBER
Read	66AJ100	Brittle diabetes	Diabetes mellitus	CALIBER
Read	F420200	Preproliferative diabetic retinopathy	Diabetes mellitus	CALIBER
Read	F420100	Proliferative diabetic retinopathy	Diabetes mellitus	CALIBER
Read	F420400	Diabetic maculopathy	Diabetes mellitus	CALIBER
Read	C109.00	Non-insulin dependent diabetes mellitus	Diabetes mellitus	CALIBER
Read	F372.11	Diabetic polyneuropathy	Diabetes mellitus	CALIBER
Read	C109.11	NIDDM - Non-insulin dependent diabetes mellitus	Diabetes mellitus	CALIBER
Read	66AS.00	Diabetic annual review	Diabetes mellitus	CALIBER
Read	C108700	Insulin dependent diabetes mellitus with retinopathy	Diabetes mellitus	CALIBER
Read	C108800	Insulin dependent diabetes mellitus - poor control	Diabetes mellitus	CALIBER
Read	8H2J.00	Admit diabetic emergency	Diabetes mellitus	CALIBER
Read	F420000	Background diabetic retinopathy	Diabetes mellitus	CALIBER
Read	M037200	Cellulitis in diabetic foot	Diabetes mellitus	CALIBER
Read	66A3.00	Diabetic on diet only	Diabetes mellitus	CALIBER
Read	C106.12	Diabetes mellitus with neuropathy	Diabetes mellitus	CALIBER

Read	C109700	Non-insulin dependent diabetes mellitus - poor control	Diabetes mellitus	CALIBER
Read	8CA4100	Pt advised re diabetic diet	Diabetes mellitus	CALIBER
Read	66AR.00	Diabetes management plan given	Diabetes mellitus	CALIBER
Read	66A5.00	Diabetic on insulin	Diabetes mellitus	CALIBER
Read	66AJ.11	Unstable diabetes	Diabetes mellitus	CALIBER
Read	2BBL.00	O/E - diabetic maculopathy present both eyes	Diabetes mellitus	CALIBER
Read	M271200	Mixed diabetic ulcer - foot	Diabetes mellitus	CALIBER
Read	C10yy00	Other specified diabetes mellitus with other spec comps	Diabetes mellitus	CALIBER
Read	F420300	Advanced diabetic maculopathy	Diabetes mellitus	CALIBER
Read	C10ED00	Type 1 diabetes mellitus with nephropathy	Diabetes mellitus	CALIBER
Read	C10ED00	Type 1 diabetes mellitus with nephropathy	Diabetes mellitus	CALIBER
Read	ZC2C800	Dietary advice for diabetes mellitus	Diabetes mellitus	CALIBER
Read	F464000	Diabetic cataract	Diabetes mellitus	CALIBER
Read	C10EM00	Type 1 diabetes mellitus with ketoacidosis	Diabetes mellitus	CALIBER
Read	C10EM00	Type 1 diabetes mellitus with ketoacidosis	Diabetes mellitus	CALIBER
Read	F420600	Non proliferative diabetic retinopathy	Diabetes mellitus	CALIBER
Read	66Ac.00	Diabetic peripheral neuropathy screening	Diabetes mellitus	CALIBER
Read	8HBG.00	Diabetic retinopathy 12 month review	Diabetes mellitus	CALIBER
Read	2BBQ.00	O/E - left eye background diabetic retinopathy	Diabetes mellitus	CALIBER
Read	2BBP.00	O/E - right eye background diabetic retinopathy	Diabetes mellitus	CALIBER
Read	8B3l.00	Diabetes medication review	Diabetes mellitus	CALIBER
Read	C10B.00	Diabetes mellitus induced by steroids	Diabetes mellitus	CALIBER
Read	7276	Pan retinal photocoagulation for diabetes	Diabetes mellitus	CALIBER
Read	F420z00	Diabetic retinopathy NOS	Diabetes mellitus	CALIBER
Read	M271100	Neuropathic diabetic ulcer - foot	Diabetes mellitus	CALIBER

Read	8H7r.00	Refer to diabetic foot screener	Diabetes mellitus	CALIBER
Read	C314.11	Renal diabetes	Diabetes mellitus	CALIBER
Read	8BL2.00	Patient on maximal tolerated therapy for diabetes	Diabetes mellitus	CALIBER
Read	8I3X.00	Diabetic retinopathy screening refused	Diabetes mellitus	CALIBER
Read	66AU.00	Diabetes care by hospital only	Diabetes mellitus	CALIBER
Read	C10E.11	Type I diabetes mellitus	Diabetes mellitus	CALIBER
Read	C10E.11	Type I diabetes mellitus	Diabetes mellitus	CALIBER
Read	8CAQ.00	Advice about blood glucose control	Diabetes mellitus	CALIBER
Read	66AP.00	Diabetes: practice programme	Diabetes mellitus	CALIBER
Read	C10FC00	Type 2 diabetes mellitus with nephropathy	Diabetes mellitus	CALIBER
Read	C10FC00	Type 2 diabetes mellitus with nephropathy	Diabetes mellitus	CALIBER
Read	66AQ.00	Diabetes: shared care programme	Diabetes mellitus	CALIBER
Read	C10F500	Type 2 diabetes mellitus with gangrene	Diabetes mellitus	CALIBER
Read	C10F500	Type 2 diabetes mellitus with gangrene	Diabetes mellitus	CALIBER
Read	66A8.00	Has seen dietician - diabetes	Diabetes mellitus	CALIBER
Read	66AI.00	Diabetic - good control	Diabetes mellitus	CALIBER
Read	13B1.00	Diabetic diet	Diabetes mellitus	CALIBER
Read	13AC.00	Diabetic weight reducing diet	Diabetes mellitus	CALIBER
Read	2BBT.00	O/E - right eye proliferative diabetic retinopathy	Diabetes mellitus	CALIBER
Read	2BBR.00	O/E - right eye preproliferative diabetic retinopathy	Diabetes mellitus	CALIBER
Read	2BBV.00	O/E - left eye proliferative diabetic retinopathy	Diabetes mellitus	CALIBER
Read	2BBW.00	O/E - right eye diabetic maculopathy	Diabetes mellitus	CALIBER
Read	2BBS.00	O/E - left eye preproliferative diabetic retinopathy	Diabetes mellitus	CALIBER
Read	2BBX.00	O/E - left eye diabetic maculopathy	Diabetes mellitus	CALIBER
Read	66AD.00	Fundoscopy - diabetic check	Diabetes mellitus	CALIBER

Read	C104y00	Other specified diabetes mellitus with renal complications	Diabetes mellitus	CALIBER
Read	C100100	Diabetes mellitus, adult onset, no mention of complication	Diabetes mellitus	CALIBER
Read	C100111	Maturity onset diabetes	Diabetes mellitus	CALIBER
Read	C103.00	Diabetes mellitus with ketoacidotic coma	Diabetes mellitus	CALIBER
Read	C106.00	Diabetes mellitus with neurological manifestation	Diabetes mellitus	CALIBER
Read	66AH.00	Diabetic treatment changed	Diabetes mellitus	CALIBER
Read	C106.13	Diabetes mellitus with polyneuropathy	Diabetes mellitus	CALIBER
Read	C104.00	Diabetes mellitus with renal manifestation	Diabetes mellitus	CALIBER
Read	ZV65312	[V]Dietary counselling in diabetes mellitus	Diabetes mellitus	CALIBER
Read	F171100	Autonomic neuropathy due to diabetes	Diabetes mellitus	CALIBER
Read	2G5A.00	O/E - Right diabetic foot at risk	Diabetes mellitus	CALIBER
Read	F35z000	Diabetic mononeuritis NOS	Diabetes mellitus	CALIBER
Read	C109600	Non-insulin-dependent diabetes mellitus with retinopathy	Diabetes mellitus	CALIBER
Read	F440700	Diabetic iritis	Diabetes mellitus	CALIBER
Read	C108F11	Type I diabetes mellitus with diabetic cataract	Diabetes mellitus	CALIBER
Read	C108F11	Type I diabetes mellitus with diabetic cataract	Diabetes mellitus	CALIBER
Read	7L19800	Subcutaneous injection of insulin	Diabetes mellitus	CALIBER
Read	C108.12	Type 1 diabetes mellitus	Diabetes mellitus	CALIBER
Read	C108.12	Type 1 diabetes mellitus	Diabetes mellitus	CALIBER
Read	C109.12	Type 2 diabetes mellitus	Diabetes mellitus	CALIBER
Read	C109.12	Type 2 diabetes mellitus	Diabetes mellitus	CALIBER
Read	66AL.00	Diabetic-uncooperative patient	Diabetes mellitus	CALIBER
Read	2G5C.00	Foot abnormality - diabetes related	Diabetes mellitus	CALIBER
Read	N030000	Diabetic cheiroarthropathy	Diabetes mellitus	CALIBER
Read	C109G11	Type II diabetes mellitus with arthropathy	Diabetes mellitus	CALIBER

Read	C109G11	Type II diabetes mellitus with arthropathy	Diabetes mellitus	CALIBER
Read	66AT.00	Annual diabetic blood test	Diabetes mellitus	CALIBER
Read	C109012	Type 2 diabetes mellitus with renal complications	Diabetes mellitus	CALIBER
Read	C109012	Type 2 diabetes mellitus with renal complications	Diabetes mellitus	CALIBER
Read	C109.13	Type II diabetes mellitus	Diabetes mellitus	CALIBER
Read	C109.13	Type II diabetes mellitus	Diabetes mellitus	CALIBER
Read	C108J12	Type 1 diabetes mellitus with neuropathic arthropathy	Diabetes mellitus	CALIBER
Read	C108J12	Type 1 diabetes mellitus with neuropathic arthropathy	Diabetes mellitus	CALIBER
Read	C109J12	Insulin treated Type II diabetes mellitus	Diabetes mellitus	CALIBER
Read	C109J12	Insulin treated Type II diabetes mellitus	Diabetes mellitus	CALIBER
Read	C109J00	Insulin treated Type 2 diabetes mellitus	Diabetes mellitus	CALIBER
Read	C109J00	Insulin treated Type 2 diabetes mellitus	Diabetes mellitus	CALIBER
Read	68A7.00	Diabetic retinopathy screening	Diabetes mellitus	CALIBER
Read	C10E700	Type 1 diabetes mellitus with retinopathy	Diabetes mellitus	CALIBER
Read	C10E700	Type 1 diabetes mellitus with retinopathy	Diabetes mellitus	CALIBER
Read	C10FM00	Type 2 diabetes mellitus with persistent microalbuminuria	Diabetes mellitus	CALIBER
Read	C10FM00	Type 2 diabetes mellitus with persistent microalbuminuria	Diabetes mellitus	CALIBER
Read	C10FB00	Type 2 diabetes mellitus with polyneuropathy	Diabetes mellitus	CALIBER
Read	C10FB00	Type 2 diabetes mellitus with polyneuropathy	Diabetes mellitus	CALIBER
Read	C10F600	Type 2 diabetes mellitus with retinopathy	Diabetes mellitus	CALIBER
Read	C10F600	Type 2 diabetes mellitus with retinopathy	Diabetes mellitus	CALIBER
Read	C108.11	IDDM-Insulin dependent diabetes mellitus	Diabetes mellitus	CALIBER
Read	C10EH00	Type 1 diabetes mellitus with arthropathy	Diabetes mellitus	CALIBER
Read	C10EH00	Type 1 diabetes mellitus with arthropathy	Diabetes mellitus	CALIBER
Read	8HBH.00	Diabetic retinopathy 6 month review	Diabetes mellitus	CALIBER

Read	C10E500	Type 1 diabetes mellitus with ulcer	Diabetes mellitus	CALIBER
Read	C10E500	Type 1 diabetes mellitus with ulcer	Diabetes mellitus	CALIBER
Read	C10F000	Type 2 diabetes mellitus with renal complications	Diabetes mellitus	CALIBER
Read	C10F000	Type 2 diabetes mellitus with renal complications	Diabetes mellitus	CALIBER
Read	8I3W.00	Diabetic foot examination declined	Diabetes mellitus	CALIBER
Read	68A9.00	Diabetic retinopathy screening offered	Diabetes mellitus	CALIBER
Read	66AA.11	Injection sites - diabetic	Diabetes mellitus	CALIBER
Read	Q441.00	Neonatal diabetes mellitus	Diabetes mellitus	CALIBER
Read	C102.00	Diabetes mellitus with hyperosmolar coma	Diabetes mellitus	CALIBER
Read	13AB.00	Diabetic lipid lowering diet	Diabetes mellitus	CALIBER
Read	C108012	Type 1 diabetes mellitus with renal complications	Diabetes mellitus	CALIBER
Read	C108012	Type 1 diabetes mellitus with renal complications	Diabetes mellitus	CALIBER
Read	66AJz00	Diabetic - poor control NOS	Diabetes mellitus	CALIBER
Read	C10N.00	Secondary diabetes mellitus	Diabetes mellitus	CALIBER
Read	C106z00	Diabetes mellitus NOS with neurological manifestation	Diabetes mellitus	CALIBER
Read	66Ab.00	Diabetic foot examination	Diabetes mellitus	CALIBER
Read	C10EP00	Type 1 diabetes mellitus with exudative maculopathy	Diabetes mellitus	CALIBER
Read	C10EP00	Type 1 diabetes mellitus with exudative maculopathy	Diabetes mellitus	CALIBER
Read	C10F.11	Type II diabetes mellitus	Diabetes mellitus	CALIBER
Read	C10F.11	Type II diabetes mellitus	Diabetes mellitus	CALIBER
Read	2BBF.00	Retinal abnormality - diabetes related	Diabetes mellitus	CALIBER
Read	C350011	Bronzed diabetes	Diabetes mellitus	CALIBER
Read	M271000	Ischaemic ulcer diabetic foot	Diabetes mellitus	CALIBER
Read	8A13.00	Diabetic stabilisation	Diabetes mellitus	CALIBER
Read	C108.13	Type I diabetes mellitus	Diabetes mellitus	CALIBER

Read	C108.13	Type I diabetes mellitus	Diabetes mellitus	CALIBER
Read	C109711	Type II diabetes mellitus - poor control	Diabetes mellitus	CALIBER
Read	C109711	Type II diabetes mellitus - poor control	Diabetes mellitus	CALIBER
Read	C100000	Diabetes mellitus, juvenile type, no mention of complication	Diabetes mellitus	CALIBER
Read	F372200	Asymptomatic diabetic neuropathy	Diabetes mellitus	CALIBER
Read	C109G00	Non-insulin dependent diabetes mellitus with arthropathy	Diabetes mellitus	CALIBER
Read	C108B00	Insulin dependent diabetes mellitus with mononeuropathy	Diabetes mellitus	CALIBER
Read	C109C12	Type 2 diabetes mellitus with nephropathy	Diabetes mellitus	CALIBER
Read	C109C12	Type 2 diabetes mellitus with nephropathy	Diabetes mellitus	CALIBER
Read	ZC2CA00	Dietary advice for type II diabetes	Diabetes mellitus	CALIBER
Read	C10FQ00	Type 2 diabetes mellitus with exudative maculopathy	Diabetes mellitus	CALIBER
Read	C10FQ00	Type 2 diabetes mellitus with exudative maculopathy	Diabetes mellitus	CALIBER
Read	C10F700	Type 2 diabetes mellitus - poor control	Diabetes mellitus	CALIBER
Read	C10F700	Type 2 diabetes mellitus - poor control	Diabetes mellitus	CALIBER
Read	66Aa.00	Diabetic diet - poor compliance	Diabetes mellitus	CALIBER
Read	C10FL00	Type 2 diabetes mellitus with persistent proteinuria	Diabetes mellitus	CALIBER
Read	C10FL00	Type 2 diabetes mellitus with persistent proteinuria	Diabetes mellitus	CALIBER
Read	C10B000	Steroid induced diabetes mellitus without complication	Diabetes mellitus	CALIBER
Read	66AY.00	Diabetic diet - good compliance	Diabetes mellitus	CALIBER
Read	2G5B.00	O/E - Left diabetic foot at risk	Diabetes mellitus	CALIBER
Read	2G5E.00	O/E - Right diabetic foot at low risk	Diabetes mellitus	CALIBER
Read	2G5I.00	O/E - Left diabetic foot at low risk	Diabetes mellitus	CALIBER
Read	C108400	Unstable insulin dependent diabetes mellitus	Diabetes mellitus	CALIBER
Read	N030100	Diabetic Charcot arthropathy	Diabetes mellitus	CALIBER
Read	2G51000	Foot abnormality - diabetes related	Diabetes mellitus	CALIBER

Read	66AV.00	Diabetic on insulin and oral treatment	Diabetes mellitus	CALIBER
Read	8CP2.00	Transition of diabetes care options discussed	Diabetes mellitus	CALIBER
Read	66Ai.00	Diabetic 6 month review	Diabetes mellitus	CALIBER
Read	66AN.00	Date diabetic treatment start	Diabetes mellitus	CALIBER
Read	C109900	Non-insulin-dependent diabetes mellitus without complication	Diabetes mellitus	CALIBER
Read	C10EL00	Type 1 diabetes mellitus with persistent microalbuminuria	Diabetes mellitus	CALIBER
Read	C10EL00	Type 1 diabetes mellitus with persistent microalbuminuria	Diabetes mellitus	CALIBER
Read	C10EK00	Type 1 diabetes mellitus with persistent proteinuria	Diabetes mellitus	CALIBER
Read	C10EK00	Type 1 diabetes mellitus with persistent proteinuria	Diabetes mellitus	CALIBER
Read	F420700	High risk proliferative diabetic retinopathy	Diabetes mellitus	CALIBER
Read	R054300	[D]Widespread diabetic foot gangrene	Diabetes mellitus	CALIBER
Read	2G5J.00	O/E - Left diabetic foot at moderate risk	Diabetes mellitus	CALIBER
Read	2G5F.00	O/E - Right diabetic foot at moderate risk	Diabetes mellitus	CALIBER
Read	2G5G.00	O/E - Right diabetic foot at high risk	Diabetes mellitus	CALIBER
Read	2G5K.00	O/E - Left diabetic foot at high risk	Diabetes mellitus	CALIBER
Read	C108900	Insulin dependent diabetes maturity onset	Diabetes mellitus	CALIBER
Read	F372.00	Polyneuropathy in diabetes	Diabetes mellitus	CALIBER
Read	C11y000	Steroid induced diabetes	Diabetes mellitus	CALIBER
Read	ZRbH.00	Perceived control of insulin-dependent diabetes	Diabetes mellitus	CALIBER
Read	C107.11	Diabetes mellitus with gangrene	Diabetes mellitus	CALIBER
Read	C107.12	Diabetes with gangrene	Diabetes mellitus	CALIBER
Read	C10FN00	Type 2 diabetes mellitus with ketoacidosis	Diabetes mellitus	CALIBER
Read	C10FN00	Type 2 diabetes mellitus with ketoacidosis	Diabetes mellitus	CALIBER
Read	C105.00	Diabetes mellitus with ophthalmic manifestation	Diabetes mellitus	CALIBER
Read	C10y.00	Diabetes mellitus with other specified manifestation	Diabetes mellitus	CALIBER

Read	C107200	Diabetes mellitus, adult with gangrene	Diabetes mellitus	CALIBER
Read	C10A100	Malnutrition-related diabetes mellitus with ketoacidosis	Diabetes mellitus	CALIBER
Read	G73y000	Diabetic peripheral angiopathy	Diabetes mellitus	CALIBER
Read	C10F200	Type 2 diabetes mellitus with neurological complications	Diabetes mellitus	CALIBER
Read	C10F200	Type 2 diabetes mellitus with neurological complications	Diabetes mellitus	CALIBER
Read	C105z00	Diabetes mellitus NOS with ophthalmic manifestation	Diabetes mellitus	CALIBER
Read	C10FK00	Hyperosmolar non-ketotic state in type 2 diabetes mellitus	Diabetes mellitus	CALIBER
Read	C10FK00	Hyperosmolar non-ketotic state in type 2 diabetes mellitus	Diabetes mellitus	CALIBER
Read	C109400	Non-insulin dependent diabetes mellitus with ulcer	Diabetes mellitus	CALIBER
Read	C104100	Diabetes mellitus, adult onset, with renal manifestation	Diabetes mellitus	CALIBER
Read	C104z00	Diabetes mellitus with nephropathy NOS	Diabetes mellitus	CALIBER
Read	2G5L.00	O/E - Left diabetic foot - ulcerated	Diabetes mellitus	CALIBER
Read	C10E800	Type 1 diabetes mellitus - poor control	Diabetes mellitus	CALIBER
Read	C10E800	Type 1 diabetes mellitus - poor control	Diabetes mellitus	CALIBER
Read	2G5H.00	O/E - Right diabetic foot - ulcerated	Diabetes mellitus	CALIBER
Read	8H3O.00	Non-urgent diabetic admission	Diabetes mellitus	CALIBER
Read	9OLD.00	Diabetic patient unsuitable for digital retinal photography	Diabetes mellitus	CALIBER
Read	C10FH00	Type 2 diabetes mellitus with neuropathic arthropathy	Diabetes mellitus	CALIBER
Read	C10FH00	Type 2 diabetes mellitus with neuropathic arthropathy	Diabetes mellitus	CALIBER
Read	C107.00	Diabetes mellitus with peripheral circulatory disorder	Diabetes mellitus	CALIBER
Read	F372100	Chronic painful diabetic neuropathy	Diabetes mellitus	CALIBER
Read	C109K00	Hyperosmolar non-ketotic state in type 2 diabetes mellitus	Diabetes mellitus	CALIBER
Read	C109K00	Hyperosmolar non-ketotic state in type 2 diabetes mellitus	Diabetes mellitus	CALIBER
Read	C10D.00	Diabetes mellitus autosomal dominant type 2	Diabetes mellitus	CALIBER
Read	7L10000	Continuous subcutaneous infusion of insulin	Diabetes mellitus	CALIBER

Read	F3y0.00	Diabetic mononeuropathy	Diabetes mellitus	CALIBER
Read	C109J11	Insulin treated non-insulin dependent diabetes mellitus	Diabetes mellitus	CALIBER
Read	C10FF00	Type 2 diabetes mellitus with peripheral angiopathy	Diabetes mellitus	CALIBER
Read	C10FF00	Type 2 diabetes mellitus with peripheral angiopathy	Diabetes mellitus	CALIBER
Read	M21yC00	Insulin lipohypertrophy	Diabetes mellitus	CALIBER
Read	66A9.00	Understands diet - diabetes	Diabetes mellitus	CALIBER
Read	C108711	Type I diabetes mellitus with retinopathy	Diabetes mellitus	CALIBER
Read	C108711	Type I diabetes mellitus with retinopathy	Diabetes mellitus	CALIBER
Read	C101y00	Other specified diabetes mellitus with ketoacidosis	Diabetes mellitus	CALIBER
Read	C100.00	Diabetes mellitus with no mention of complication	Diabetes mellitus	CALIBER
Read	C10EE00	Type 1 diabetes mellitus with hypoglycaemic coma	Diabetes mellitus	CALIBER
Read	C10EE00	Type 1 diabetes mellitus with hypoglycaemic coma	Diabetes mellitus	CALIBER
Read	C106100	Diabetes mellitus, adult onset, + neurological manifestation	Diabetes mellitus	CALIBER
Read	F381300	Myasthenic syndrome due to diabetic amyotrophy	Diabetes mellitus	CALIBER
Read	C108J00	Insulin dependent diab mell with neuropathic arthropathy	Diabetes mellitus	CALIBER
Read	C102000	Diabetes mellitus, juvenile type, with hyperosmolar coma	Diabetes mellitus	CALIBER
Read	C109500	Non-insulin dependent diabetes mellitus with gangrene	Diabetes mellitus	CALIBER
Read	C10E900	Type 1 diabetes mellitus maturity onset	Diabetes mellitus	CALIBER
Read	C10E900	Type 1 diabetes mellitus maturity onset	Diabetes mellitus	CALIBER
Read	C10EN00	Type 1 diabetes mellitus with ketoacidotic coma	Diabetes mellitus	CALIBER
Read	C10EN00	Type 1 diabetes mellitus with ketoacidotic coma	Diabetes mellitus	CALIBER
Read	C109H00	Non-insulin dependent d m with neuropathic arthropathy	Diabetes mellitus	CALIBER
Read	C108712	Type 1 diabetes mellitus with retinopathy	Diabetes mellitus	CALIBER
Read	C108712	Type 1 diabetes mellitus with retinopathy	Diabetes mellitus	CALIBER
Read	C105100	Diabetes mellitus, adult onset, + ophthalmic manifestation	Diabetes mellitus	CALIBER

Read	Cyu2000	[X]Other specified diabetes mellitus	Diabetes mellitus	CALIBER
Read	C108C00	Insulin dependent diabetes mellitus with polyneuropathy	Diabetes mellitus	CALIBER
Read	C101z00	Diabetes mellitus NOS with ketoacidosis	Diabetes mellitus	CALIBER
Read	C103000	Diabetes mellitus, juvenile type, with ketoacidotic coma	Diabetes mellitus	CALIBER
Read	C108E11	Type I diabetes mellitus with hypoglycaemic coma	Diabetes mellitus	CALIBER
Read	C108E11	Type I diabetes mellitus with hypoglycaemic coma	Diabetes mellitus	CALIBER
Read	C109612	Type 2 diabetes mellitus with retinopathy	Diabetes mellitus	CALIBER
Read	C109612	Type 2 diabetes mellitus with retinopathy	Diabetes mellitus	CALIBER
Read	C10E200	Type 1 diabetes mellitus with neurological complications	Diabetes mellitus	CALIBER
Read	C10E200	Type 1 diabetes mellitus with neurological complications	Diabetes mellitus	CALIBER
Read	C102100	Diabetes mellitus, adult onset, with hyperosmolar coma	Diabetes mellitus	CALIBER
Read	C10F311	Type II diabetes mellitus with multiple complications	Diabetes mellitus	CALIBER
Read	C10F311	Type II diabetes mellitus with multiple complications	Diabetes mellitus	CALIBER
Read	C10C.00	Diabetes mellitus autosomal dominant	Diabetes mellitus	CALIBER
Read	M21yC11	Insulin site lipohypertrophy	Diabetes mellitus	CALIBER
Read	C109D00	Non-insulin dependent diabetes mellitus with hypoglyca coma	Diabetes mellitus	CALIBER
Read	C10M.00	Lipoatrophic diabetes mellitus	Diabetes mellitus	CALIBER
Read	C10E400	Unstable type 1 diabetes mellitus	Diabetes mellitus	CALIBER
Read	C10E400	Unstable type 1 diabetes mellitus	Diabetes mellitus	CALIBER
Read	66AK.00	Diabetic - cooperative patient	Diabetes mellitus	CALIBER
Read	F345000	Diabetic mononeuritis multiplex	Diabetes mellitus	CALIBER
Read	C108F00	Insulin dependent diabetes mellitus with diabetic cataract	Diabetes mellitus	CALIBER
Read	C108E00	Insulin dependent diabetes mellitus with hypoglycaemic coma	Diabetes mellitus	CALIBER
Read	C108500	Insulin dependent diabetes mellitus with ulcer	Diabetes mellitus	CALIBER
Read	C109E12	Type 2 diabetes mellitus with diabetic cataract	Diabetes mellitus	CALIBER

Read	C109E12	Type 2 diabetes mellitus with diabetic cataract	Diabetes mellitus	CALIBER
Read	C10FE00	Type 2 diabetes mellitus with diabetic cataract	Diabetes mellitus	CALIBER
Read	C10FE00	Type 2 diabetes mellitus with diabetic cataract	Diabetes mellitus	CALIBER
Read	C10E312	Insulin dependent diabetes mellitus with multiple complicat	Diabetes mellitus	CALIBER
Read	C109B00	Non-insulin dependent diabetes mellitus with polyneuropathy	Diabetes mellitus	CALIBER
Read	C10z.00	Diabetes mellitus with unspecified complication	Diabetes mellitus	CALIBER
Read	K01x111	Kimmelstiel - Wilson disease	Diabetes mellitus	CALIBER
Read	C109712	Type 2 diabetes mellitus - poor control	Diabetes mellitus	CALIBER
Read	C109712	Type 2 diabetes mellitus - poor control	Diabetes mellitus	CALIBER
Read	C108812	Type 1 diabetes mellitus - poor control	Diabetes mellitus	CALIBER
Read	C108812	Type 1 diabetes mellitus - poor control	Diabetes mellitus	CALIBER
Read	C109212	Type 2 diabetes mellitus with neurological complications	Diabetes mellitus	CALIBER
Read	C109212	Type 2 diabetes mellitus with neurological complications	Diabetes mellitus	CALIBER
Read	C109512	Type 2 diabetes mellitus with gangrene	Diabetes mellitus	CALIBER
Read	C109512	Type 2 diabetes mellitus with gangrene	Diabetes mellitus	CALIBER
Read	C108y00	Other specified diabetes mellitus with multiple comps	Diabetes mellitus	CALIBER
Read	C10EC00	Type 1 diabetes mellitus with polyneuropathy	Diabetes mellitus	CALIBER
Read	C10EC00	Type 1 diabetes mellitus with polyneuropathy	Diabetes mellitus	CALIBER
Read	66AX.00	Diabetes: shared care in pregnancy - diabetol and obstet	Diabetes mellitus	CALIBER
Read	C10C.11	Maturity onset diabetes in youth	Diabetes mellitus	CALIBER
Read	C108811	Type I diabetes mellitus - poor control	Diabetes mellitus	CALIBER
Read	C108811	Type I diabetes mellitus - poor control	Diabetes mellitus	CALIBER
Read	C10FD00	Type 2 diabetes mellitus with hypoglycaemic coma	Diabetes mellitus	CALIBER
Read	C10FD00	Type 2 diabetes mellitus with hypoglycaemic coma	Diabetes mellitus	CALIBER

Read	C108000	Insulin-dependent diabetes mellitus with renal complications	Diabetes mellitus	CALIBER
Read	2BBM.00	O/E - diabetic maculopathy absent both eyes	Diabetes mellitus	CALIBER
Read	C10F711	Type II diabetes mellitus - poor control	Diabetes mellitus	CALIBER
Read	C10F711	Type II diabetes mellitus - poor control	Diabetes mellitus	CALIBER
Read	C10F100	Type 2 diabetes mellitus with ophthalmic complications	Diabetes mellitus	CALIBER
Read	C10F100	Type 2 diabetes mellitus with ophthalmic complications	Diabetes mellitus	CALIBER
Read	2BBk.00	O/E - right eye stable treated prolif diabetic retinopathy	Diabetes mellitus	CALIBER
Read	8HLE.00	Diabetology D.V. done	Diabetes mellitus	CALIBER
Read	C105y00	Other specified diabetes mellitus with ophthalmic complicatn	Diabetes mellitus	CALIBER
Read	C109B11	Type II diabetes mellitus with polyneuropathy	Diabetes mellitus	CALIBER
Read	C109B11	Type II diabetes mellitus with polyneuropathy	Diabetes mellitus	CALIBER
Read	C10E000	Type 1 diabetes mellitus with renal complications	Diabetes mellitus	CALIBER
Read	C10E000	Type 1 diabetes mellitus with renal complications	Diabetes mellitus	CALIBER
Read	F420500	Advanced diabetic retinal disease	Diabetes mellitus	CALIBER
Read	C10E100	Type 1 diabetes mellitus with ophthalmic complications	Diabetes mellitus	CALIBER
Read	C10E100	Type 1 diabetes mellitus with ophthalmic complications	Diabetes mellitus	CALIBER
Read	C10E300	Type 1 diabetes mellitus with multiple complications	Diabetes mellitus	CALIBER
Read	C10E300	Type 1 diabetes mellitus with multiple complications	Diabetes mellitus	CALIBER
Read	C109H11	Type II diabetes mellitus with neuropathic arthropathy	Diabetes mellitus	CALIBER
Read	C109H11	Type II diabetes mellitus with neuropathic arthropathy	Diabetes mellitus	CALIBER
Read	C10F900	Type 2 diabetes mellitus without complication	Diabetes mellitus	CALIBER
Read	C10F900	Type 2 diabetes mellitus without complication	Diabetes mellitus	CALIBER
Read	F372000	Acute painful diabetic neuropathy	Diabetes mellitus	CALIBER
Read	C109E11	Type II diabetes mellitus with diabetic cataract	Diabetes mellitus	CALIBER

Read	C109E11	Type II diabetes mellitus with diabetic cataract	Diabetes mellitus	CALIBER
Read	C10F400	Type 2 diabetes mellitus with ulcer	Diabetes mellitus	CALIBER
Read	C10F400	Type 2 diabetes mellitus with ulcer	Diabetes mellitus	CALIBER
Read	C108211	Type I diabetes mellitus with neurological complications	Diabetes mellitus	CALIBER
Read	C108211	Type I diabetes mellitus with neurological complications	Diabetes mellitus	CALIBER
Read	C108100	Insulin-dependent diabetes mellitus with ophthalmic comps	Diabetes mellitus	CALIBER
Read	C10EF00	Type 1 diabetes mellitus with diabetic cataract	Diabetes mellitus	CALIBER
Read	C10EF00	Type 1 diabetes mellitus with diabetic cataract	Diabetes mellitus	CALIBER
Read	2G5W.00	O/E - left chronic diabetic foot ulcer	Diabetes mellitus	CALIBER
Read	C10F611	Type II diabetes mellitus with retinopathy	Diabetes mellitus	CALIBER
Read	C10F611	Type II diabetes mellitus with retinopathy	Diabetes mellitus	CALIBER
Read	C109G12	Type 2 diabetes mellitus with arthropathy	Diabetes mellitus	CALIBER
Read	C109G12	Type 2 diabetes mellitus with arthropathy	Diabetes mellitus	CALIBER
Read	6761	Diabetic pre-pregnancy counselling	Diabetes mellitus	CALIBER
Read	C10E411	Unstable type I diabetes mellitus	Diabetes mellitus	CALIBER
Read	C10E411	Unstable type I diabetes mellitus	Diabetes mellitus	CALIBER
Read	66AW.00	Diabetic foot risk assessment	Diabetes mellitus	CALIBER
Read	C109011	Type II diabetes mellitus with renal complications	Diabetes mellitus	CALIBER
Read	C109011	Type II diabetes mellitus with renal complications	Diabetes mellitus	CALIBER
Read	C109100	Non-insulin-dependent diabetes mellitus with ophthalm comps	Diabetes mellitus	CALIBER
Read	C10FB11	Type II diabetes mellitus with polyneuropathy	Diabetes mellitus	CALIBER
Read	C10FB11	Type II diabetes mellitus with polyneuropathy	Diabetes mellitus	CALIBER
Read	L180600	Pre-existing diabetes mellitus, non-insulin-dependent	Diabetes mellitus	CALIBER
Read	C109A11	Type II diabetes mellitus with mononeuropathy	Diabetes mellitus	CALIBER

Read	C109A11	Type II diabetes mellitus with mononeuropathy	Diabetes mellitus	CALIBER
Read	L180500	Pre-existing diabetes mellitus, insulin-dependent	Diabetes mellitus	CALIBER
Read	C100z00	Diabetes mellitus NOS with no mention of complication	Diabetes mellitus	CALIBER
Read	C10E.12	Insulin dependent diabetes mellitus	Diabetes mellitus	CALIBER
Read	C10G.00	Secondary pancreatic diabetes mellitus	Diabetes mellitus	CALIBER
Read	C10FP00	Type 2 diabetes mellitus with ketoacidotic coma	Diabetes mellitus	CALIBER
Read	C10FP00	Type 2 diabetes mellitus with ketoacidotic coma	Diabetes mellitus	CALIBER
Read	C108511	Type I diabetes mellitus with ulcer	Diabetes mellitus	CALIBER
Read	C108511	Type I diabetes mellitus with ulcer	Diabetes mellitus	CALIBER
Read	2BBl.00	O/E - left eye stable treated prolif diabetic retinopathy	Diabetes mellitus	CALIBER
Read	C108300	Insulin dependent diabetes mellitus with multiple complicatn	Diabetes mellitus	CALIBER
Read	Cyu2.00	[X]Diabetes mellitus	Diabetes mellitus	CALIBER
Read	C10A.00	Malnutrition-related diabetes mellitus	Diabetes mellitus	CALIBER
Read	9360	Patient held diabetic record issued	Diabetes mellitus	CALIBER
Read	C108200	Insulin-dependent diabetes mellitus with neurological comps	Diabetes mellitus	CALIBER
Read	C109000	Non-insulin-dependent diabetes mellitus with renal comps	Diabetes mellitus	CALIBER
Read	2BBo.00	O/E - sight threatening diabetic retinopathy	Diabetes mellitus	CALIBER
Read	C101000	Diabetes mellitus, juvenile type, with ketoacidosis	Diabetes mellitus	CALIBER
Read	66AG.00	Diabetic drug side effects	Diabetes mellitus	CALIBER
Read	C10F911	Type II diabetes mellitus without complication	Diabetes mellitus	CALIBER
Read	C10F911	Type II diabetes mellitus without complication	Diabetes mellitus	CALIBER
Read	R054200	[D]Gangrene of toe in diabetic	Diabetes mellitus	CALIBER
Read	C10EJ00	Type 1 diabetes mellitus with neuropathic arthropathy	Diabetes mellitus	CALIBER
Read	C10EJ00	Type 1 diabetes mellitus with neuropathic arthropathy	Diabetes mellitus	CALIBER

Read	C109F00	Non-insulin-dependent d m with peripheral angiopath	Diabetes mellitus	CALIBER
Read	C10E412	Unstable insulin dependent diabetes mellitus	Diabetes mellitus	CALIBER
Read	C101100	Diabetes mellitus, adult onset, with ketoacidosis	Diabetes mellitus	CALIBER
Read	C109F11	Type II diabetes mellitus with peripheral angiopathy	Diabetes mellitus	CALIBER
Read	C109F11	Type II diabetes mellitus with peripheral angiopathy	Diabetes mellitus	CALIBER
Read	C109411	Type II diabetes mellitus with ulcer	Diabetes mellitus	CALIBER
Read	C109411	Type II diabetes mellitus with ulcer	Diabetes mellitus	CALIBER
Read	66AO.00	Date diabetic treatment stopp.	Diabetes mellitus	CALIBER
Read	C10EQ00	Type 1 diabetes mellitus with gastroparesis	Diabetes mellitus	CALIBER
Read	C10EQ00	Type 1 diabetes mellitus with gastroparesis	Diabetes mellitus	CALIBER
Read	L180X00	Pre-existing diabetes mellitus, unspecified	Diabetes mellitus	CALIBER
Read	C109200	Non-insulin-dependent diabetes mellitus with neuro comps	Diabetes mellitus	CALIBER
Read	C109D11	Type II diabetes mellitus with hypoglycaemic coma	Diabetes mellitus	CALIBER
Read	C109D11	Type II diabetes mellitus with hypoglycaemic coma	Diabetes mellitus	CALIBER
Read	C108A00	Insulin-dependent diabetes without complication	Diabetes mellitus	CALIBER
Read	C107400	NIDDM with peripheral circulatory disorder	Diabetes mellitus	CALIBER
Read	C10F011	Type II diabetes mellitus with renal complications	Diabetes mellitus	CALIBER
Read	C10F011	Type II diabetes mellitus with renal complications	Diabetes mellitus	CALIBER
Read	N030011	Diabetic cheiropathy	Diabetes mellitus	CALIBER
Read	C108D00	Insulin dependent diabetes mellitus with nephropathy	Diabetes mellitus	CALIBER
Read	8I3k.00	Insulin therapy declined	Diabetes mellitus	CALIBER
Read	C109611	Type II diabetes mellitus with retinopathy	Diabetes mellitus	CALIBER
Read	C109611	Type II diabetes mellitus with retinopathy	Diabetes mellitus	CALIBER
Read	8I57.00	Patient held diabetic record declined	Diabetes mellitus	CALIBER

Read	C10FG00	Type 2 diabetes mellitus with arthropathy	Diabetes mellitus	CALIBER
Read	C10FG00	Type 2 diabetes mellitus with arthropathy	Diabetes mellitus	CALIBER
Read	C103y00	Other specified diabetes mellitus with coma	Diabetes mellitus	CALIBER
Read	C109C00	Non-insulin dependent diabetes mellitus with nephropathy	Diabetes mellitus	CALIBER
Read	C109111	Type II diabetes mellitus with ophthalmic complications	Diabetes mellitus	CALIBER
Read	C109111	Type II diabetes mellitus with ophthalmic complications	Diabetes mellitus	CALIBER
Read	C106.11	Diabetic amyotrophy	Diabetes mellitus	CALIBER
Read	C10D.11	Maturity onset diabetes in youth type 2	Diabetes mellitus	CALIBER
Read	C108411	Unstable type I diabetes mellitus	Diabetes mellitus	CALIBER
Read	C108411	Unstable type I diabetes mellitus	Diabetes mellitus	CALIBER
Read	C108J11	Type I diabetes mellitus with neuropathic arthropathy	Diabetes mellitus	CALIBER
Read	C108J11	Type I diabetes mellitus with neuropathic arthropathy	Diabetes mellitus	CALIBER
Read	C108600	Insulin dependent diabetes mellitus with gangrene	Diabetes mellitus	CALIBER
Read	C109F12	Type 2 diabetes mellitus with peripheral angiopathy	Diabetes mellitus	CALIBER
Read	C109F12	Type 2 diabetes mellitus with peripheral angiopathy	Diabetes mellitus	CALIBER
Read	C10FL11	Type II diabetes mellitus with persistent proteinuria	Diabetes mellitus	CALIBER
Read	C10FL11	Type II diabetes mellitus with persistent proteinuria	Diabetes mellitus	CALIBER
Read	68AB.00	Diabetic digital retinopathy screening offered	Diabetes mellitus	CALIBER
Read	C109D12	Type 2 diabetes mellitus with hypoglycaemic coma	Diabetes mellitus	CALIBER
Read	C109D12	Type 2 diabetes mellitus with hypoglycaemic coma	Diabetes mellitus	CALIBER
Read	C10H.00	Diabetes mellitus induced by non-steroid drugs	Diabetes mellitus	CALIBER
Read	TJ23z00	Adverse reaction to insulins and antidiabetic agents NOS	Diabetes mellitus	CALIBER
Read	C108011	Type I diabetes mellitus with renal complications	Diabetes mellitus	CALIBER
Read	C108011	Type I diabetes mellitus with renal complications	Diabetes mellitus	CALIBER

Read	C106y00	Other specified diabetes mellitus with neurological comps	Diabetes mellitus	CALIBER
Read	889A.00	Diab mellit insulin-glucose infus acute myocardial infarct	Diabetes mellitus	CALIBER
Read	C108212	Type 1 diabetes mellitus with neurological complications	Diabetes mellitus	CALIBER
Read	C108212	Type 1 diabetes mellitus with neurological complications	Diabetes mellitus	CALIBER
Read	C109511	Type II diabetes mellitus with gangrene	Diabetes mellitus	CALIBER
Read	C109511	Type II diabetes mellitus with gangrene	Diabetes mellitus	CALIBER
Read	C109300	Non-insulin-dependent diabetes mellitus with multiple comps	Diabetes mellitus	CALIBER
Read	C10EM11	Type I diabetes mellitus with ketoacidosis	Diabetes mellitus	CALIBER
Read	C10EM11	Type I diabetes mellitus with ketoacidosis	Diabetes mellitus	CALIBER
Read	C108H11	Type I diabetes mellitus with arthropathy	Diabetes mellitus	CALIBER
Read	C108H11	Type I diabetes mellitus with arthropathy	Diabetes mellitus	CALIBER
Read	2G5V.00	O/E - right chronic diabetic foot ulcer	Diabetes mellitus	CALIBER
Read	C10EA11	Type I diabetes mellitus without complication	Diabetes mellitus	CALIBER
Read	C10EA11	Type I diabetes mellitus without complication	Diabetes mellitus	CALIBER
Read	C10FA00	Type 2 diabetes mellitus with mononeuropathy	Diabetes mellitus	CALIBER
Read	C10FA00	Type 2 diabetes mellitus with mononeuropathy	Diabetes mellitus	CALIBER
Read	C108911	Type I diabetes mellitus maturity onset	Diabetes mellitus	CALIBER
Read	C108911	Type I diabetes mellitus maturity onset	Diabetes mellitus	CALIBER
Read	C107100	Diabetes mellitus, adult, + peripheral circulatory disorder	Diabetes mellitus	CALIBER
Read	C10y100	Diabetes mellitus, adult, + other specified manifestation	Diabetes mellitus	CALIBER
Read	C10FR00	Type 2 diabetes mellitus with gastroparesis	Diabetes mellitus	CALIBER
Read	C10FR00	Type 2 diabetes mellitus with gastroparesis	Diabetes mellitus	CALIBER
Read	C10z100	Diabetes mellitus, adult onset, + unspecified complication	Diabetes mellitus	CALIBER
Read	8Hl1.00	Referral for diabetic retinopathy screening	Diabetes mellitus	CALIBER

Read	C10zy00	Other specified diabetes mellitus with unspecified comps	Diabetes mellitus	CALIBER
Read	C10zz00	Diabetes mellitus NOS with unspecified complication	Diabetes mellitus	CALIBER
Read	C108G00	Insulin dependent diab mell with peripheral angiopathy	Diabetes mellitus	CALIBER
Read	C108z00	Unspecified diabetes mellitus with multiple complications	Diabetes mellitus	CALIBER
Read	C109C11	Type II diabetes mellitus with nephropathy	Diabetes mellitus	CALIBER
Read	C109C11	Type II diabetes mellitus with nephropathy	Diabetes mellitus	CALIBER
Read	C10FJ11	Insulin treated Type II diabetes mellitus	Diabetes mellitus	CALIBER
Read	C10FJ11	Insulin treated Type II diabetes mellitus	Diabetes mellitus	CALIBER
Read	C107z00	Diabetes mellitus NOS with peripheral circulatory disorder	Diabetes mellitus	CALIBER
Read	C103z00	Diabetes mellitus NOS with ketoacidotic coma	Diabetes mellitus	CALIBER
Read	C10F300	Type 2 diabetes mellitus with multiple complications	Diabetes mellitus	CALIBER
Read	C10F300	Type 2 diabetes mellitus with multiple complications	Diabetes mellitus	CALIBER
Read	F420800	High risk non proliferative diabetic retinopathy	Diabetes mellitus	CALIBER
Read	C108H00	Insulin dependent diabetes mellitus with arthropathy	Diabetes mellitus	CALIBER
Read	U602311	[X] Adverse reaction to insulins and antidiabetic agents	Diabetes mellitus	CALIBER
Read	C109412	Type 2 diabetes mellitus with ulcer	Diabetes mellitus	CALIBER
Read	C109412	Type 2 diabetes mellitus with ulcer	Diabetes mellitus	CALIBER
Read	C10EN11	Type I diabetes mellitus with ketoacidotic coma	Diabetes mellitus	CALIBER
Read	C10EN11	Type I diabetes mellitus with ketoacidotic coma	Diabetes mellitus	CALIBER
Read	66Ah.00	Insulin needles changed for each injection	Diabetes mellitus	CALIBER
Read	C10A000	Malnutrition-related diabetes mellitus with coma	Diabetes mellitus	CALIBER
Read	C108D11	Type I diabetes mellitus with nephropathy	Diabetes mellitus	CALIBER
Read	C108D11	Type I diabetes mellitus with nephropathy	Diabetes mellitus	CALIBER
Read	C109H12	Type 2 diabetes mellitus with neuropathic arthropathy	Diabetes mellitus	CALIBER

Read	C109H12	Type 2 diabetes mellitus with neuropathic arthropathy	Diabetes mellitus	CALIBER
Read	C10H000	DM induced by non-steroid drugs without complication	Diabetes mellitus	CALIBER
Read	C106000	Diabetes mellitus, juvenile, + neurological manifestation	Diabetes mellitus	CALIBER
Read	C109211	Type II diabetes mellitus with neurological complications	Diabetes mellitus	CALIBER
Read	C109211	Type II diabetes mellitus with neurological complications	Diabetes mellitus	CALIBER
Read	C10EB00	Type 1 diabetes mellitus with mononeuropathy	Diabetes mellitus	CALIBER
Read	C10EB00	Type 1 diabetes mellitus with mononeuropathy	Diabetes mellitus	CALIBER
Read	C108512	Type 1 diabetes mellitus with ulcer	Diabetes mellitus	CALIBER
Read	C108512	Type 1 diabetes mellitus with ulcer	Diabetes mellitus	CALIBER
Read	C10z000	Diabetes mellitus, juvenile type, + unspecified complication	Diabetes mellitus	CALIBER
Read	C103100	Diabetes mellitus, adult onset, with ketoacidotic coma	Diabetes mellitus	CALIBER
Read	TJ23.00	Adverse reaction to insulins and antidiabetic agents	Diabetes mellitus	CALIBER
Read	ZC2C900	Dietary advice for type I diabetes	Diabetes mellitus	CALIBER
Read	C107300	IDDM with peripheral circulatory disorder	Diabetes mellitus	CALIBER
Read	66Aj.00	Insulin needles changed less than once a day	Diabetes mellitus	CALIBER
Read	C109E00	Non-insulin depend diabetes mellitus with diabetic cataract	Diabetes mellitus	CALIBER
Read	C10EA00	Type 1 diabetes mellitus without complication	Diabetes mellitus	CALIBER
Read	C10EA00	Type 1 diabetes mellitus without complication	Diabetes mellitus	CALIBER
Read	C105000	Diabetes mellitus, juvenile type, + ophthalmic manifestation	Diabetes mellitus	CALIBER
Read	C10E600	Type 1 diabetes mellitus with gangrene	Diabetes mellitus	CALIBER
Read	C10E600	Type 1 diabetes mellitus with gangrene	Diabetes mellitus	CALIBER
Read	C109112	Type 2 diabetes mellitus with ophthalmic complications	Diabetes mellitus	CALIBER
Read	C109112	Type 2 diabetes mellitus with ophthalmic complications	Diabetes mellitus	CALIBER
Read	C107000	Diabetes mellitus, juvenile +peripheral circulatory disorder	Diabetes mellitus	CALIBER

Read	C108E12	Type 1 diabetes mellitus with hypoglycaemic coma	Diabetes mellitus	CALIBER
Read	C108E12	Type 1 diabetes mellitus with hypoglycaemic coma	Diabetes mellitus	CALIBER
Read	C10yz00	Diabetes mellitus NOS with other specified manifestation	Diabetes mellitus	CALIBER
Read	C109A00	Non-insulin dependent diabetes mellitus with mononeuropathy	Diabetes mellitus	CALIBER
Read	C102z00	Diabetes mellitus NOS with hyperosmolar coma	Diabetes mellitus	CALIBER
Read	C10E812	Insulin dependent diabetes mellitus - poor control	Diabetes mellitus	CALIBER
Read	66Am.00	Insulin dose changed	Diabetes mellitus	CALIBER
Read	66Ao.00	Diabetes type 2 review	Diabetes mellitus	CALIBER
Read	66An.00	Diabetes type 1 review	Diabetes mellitus	CALIBER
Read	C10FM11	Type II diabetes mellitus with persistent microalbuminuria	Diabetes mellitus	CALIBER
Read	C10FM11	Type II diabetes mellitus with persistent microalbuminuria	Diabetes mellitus	CALIBER
Read	66Ag.00	Insulin needles changed daily	Diabetes mellitus	CALIBER
Read	C10F411	Type II diabetes mellitus with ulcer	Diabetes mellitus	CALIBER
Read	C10F411	Type II diabetes mellitus with ulcer	Diabetes mellitus	CALIBER
Read	C10E311	Type I diabetes mellitus with multiple complications	Diabetes mellitus	CALIBER
Read	C10E311	Type I diabetes mellitus with multiple complications	Diabetes mellitus	CALIBER
Read	C10EC11	Type I diabetes mellitus with polyneuropathy	Diabetes mellitus	CALIBER
Read	C10EC11	Type I diabetes mellitus with polyneuropathy	Diabetes mellitus	CALIBER
Read	C10N100	Cystic fibrosis related diabetes mellitus	Diabetes mellitus	CALIBER
Read	C10EG00	Type 1 diabetes mellitus with peripheral angiopathy	Diabetes mellitus	CALIBER
Read	C10EG00	Type 1 diabetes mellitus with peripheral angiopathy	Diabetes mellitus	CALIBER
Read	C10FE11	Type II diabetes mellitus with diabetic cataract	Diabetes mellitus	CALIBER
Read	C10FE11	Type II diabetes mellitus with diabetic cataract	Diabetes mellitus	CALIBER
Read	C10E712	Insulin dependent diabetes mellitus with retinopathy	Diabetes mellitus	CALIBER

Read	C10E511	Type I diabetes mellitus with ulcer	Diabetes mellitus	CALIBER
Read	C10E511	Type I diabetes mellitus with ulcer	Diabetes mellitus	CALIBER
Read	C104000	Diabetes mellitus, juvenile type, with renal manifestation	Diabetes mellitus	CALIBER
Read	C10N000	Secondary diabetes mellitus without complication	Diabetes mellitus	CALIBER
Read	C10E711	Type I diabetes mellitus with retinopathy	Diabetes mellitus	CALIBER
Read	C10E711	Type I diabetes mellitus with retinopathy	Diabetes mellitus	CALIBER
Read	C10FA11	Type II diabetes mellitus with mononeuropathy	Diabetes mellitus	CALIBER
Read	C10FA11	Type II diabetes mellitus with mononeuropathy	Diabetes mellitus	CALIBER
Read	C10FS00	Maternally inherited diabetes mellitus	Diabetes mellitus	CALIBER
Read	C10ER00	Latent autoimmune diabetes mellitus in adult	Diabetes mellitus	CALIBER
Read	C108A11	Type I diabetes mellitus without complication	Diabetes mellitus	CALIBER
Read	C108A11	Type I diabetes mellitus without complication	Diabetes mellitus	CALIBER
Read	66Aq.00	Diabetic foot screen	Diabetes mellitus	CALIBER
Read	66Ap.00	Insulin treatment initiated	Diabetes mellitus	CALIBER
Read	C10E911	Type I diabetes mellitus maturity onset	Diabetes mellitus	CALIBER
Read	C10E911	Type I diabetes mellitus maturity onset	Diabetes mellitus	CALIBER
Read	C10G000	Secondary pancreatic diabetes mellitus without complication	Diabetes mellitus	CALIBER
Read	C108912	Type 1 diabetes mellitus maturity onset	Diabetes mellitus	CALIBER
Read	C108912	Type 1 diabetes mellitus maturity onset	Diabetes mellitus	CALIBER
Read	C108412	Unstable type 1 diabetes mellitus	Diabetes mellitus	CALIBER
Read	C108412	Unstable type 1 diabetes mellitus	Diabetes mellitus	CALIBER
Read	C10E912	Insulin dependent diabetes maturity onset	Diabetes mellitus	CALIBER
Read	C10EP11	Type I diabetes mellitus with exudative maculopathy	Diabetes mellitus	CALIBER
Read	C10EP11	Type I diabetes mellitus with exudative maculopathy	Diabetes mellitus	CALIBER

Read	C10E112	Insulin-dependent diabetes mellitus with ophthalmic comps	Diabetes mellitus	CALIBER
Read	C10C.12	Maturity onset diabetes in youth type 1	Diabetes mellitus	CALIBER
Read	C10F211	Type II diabetes mellitus with neurological complications	Diabetes mellitus	CALIBER
Read	C10F211	Type II diabetes mellitus with neurological complications	Diabetes mellitus	CALIBER
Read	C10E512	Insulin dependent diabetes mellitus with ulcer	Diabetes mellitus	CALIBER
Read	C10FD11	Type II diabetes mellitus with hypoglycaemic coma	Diabetes mellitus	CALIBER
Read	C10FD11	Type II diabetes mellitus with hypoglycaemic coma	Diabetes mellitus	CALIBER
ICD10	E10	Insulin-dependent diabetes mellitus	Diabetes mellitus	CALIBER
ICD10	E11	Non-insulin-dependent diabetes mellitus	Diabetes mellitus	CALIBER
ICD10	E12	Malnutrition-related diabetes mellitus	Diabetes mellitus	CALIBER
ICD10	O242	Diabetes mellitus in pregnancy: Pre-existing malnutrition-related diabetes mellitus	Diabetes mellitus	CALIBER
ICD10	E13	Other specified diabetes mellitus	Diabetes mellitus	CALIBER
ICD10	E14	Unspecified diabetes mellitus	Diabetes mellitus	CALIBER
ICD10	G590	Diabetic mononeuropathy	Diabetes mellitus	CALIBER
ICD10	G632	Diabetic polyneuropathy	Diabetes mellitus	CALIBER
ICD10	H280	Diabetic cataract	Diabetes mellitus	CALIBER
ICD10	H360	Diabetic retinopathy	Diabetes mellitus	CALIBER
ICD10	M142	Diabetic arthropathy	Diabetes mellitus	CALIBER
ICD10	N083	Glomerular disorders in diabetes mellitus	Diabetes mellitus	CALIBER
ICD10	O240	Diabetes mellitus in pregnancy: Pre-existing diabetes mellitus, insulin-dependent	Diabetes mellitus	CALIBER
ICD10	O241	Diabetes mellitus in pregnancy: Pre-existing diabetes mellitus, non-insulin-dependent	Diabetes mellitus	CALIBER
ICD10	O243	Diabetes mellitus in pregnancy: Pre-existing diabetes mellitus, unspecified	Diabetes mellitus	CALIBER
Read	J511000	Diverticulitis of the duodenum	Diverticulitis	CALIBER
Read	J511100	Diverticulitis of the jejunum	Diverticulitis	CALIBER
Read	J511200	Diverticulitis of the ileum	Diverticulitis	CALIBER

Read	J511300	Diverticulitis of the small intestine unspecified	Diverticulitis	CALIBER
Read	J511400	Diverticulitis of the small intestine NOS	Diverticulitis	CALIBER
Read	J511500	Diverticulitis of the colon	Diverticulitis	CALIBER
Read	J511600	Diverticulitis of the large intestine unspecified	Diverticulitis	CALIBER
Read	J511700	Diverticulitis of the large intestine NOS	Diverticulitis	CALIBER
Read	J511.00	Diverticulitis	Diverticulitis	CALIBER
Read	J511y00	Diverticulitis unspecified	Diverticulitis	CALIBER
Read	J511z00	Diverticulitis NOS	Diverticulitis	CALIBER
Read	1473	H/O: epilepsy	Epilepsy	CALIBER
Read	1B1W.00	Transient epileptic amnesia	Epilepsy	CALIBER
Read	1O30.00	Epilepsy confirmed	Epilepsy	CALIBER
Read	2126000	Epilepsy resolved	Epilepsy	CALIBER
Read	212J.00	Epilepsy resolved	Epilepsy	CALIBER
Read	6110	Contraceptive advice for patients with epilepsy	Epilepsy	CALIBER
Read	6674	Epilepsy associated problems	Epilepsy	CALIBER
Read	667..00	Epilepsy monitoring	Epilepsy	CALIBER
Read	6677	Epilepsy drug side effects	Epilepsy	CALIBER
Read	6678	Epilepsy treatment changed	Epilepsy	CALIBER
Read	6679	Epilepsy treatment started	Epilepsy	CALIBER
Read	667A.00	Epilepsy treatment stopped	Epilepsy	CALIBER
Read	667B.00	Nocturnal epilepsy	Epilepsy	CALIBER
Read	667C.00	Epilepsy control good	Epilepsy	CALIBER
Read	667D.00	Epilepsy control poor	Epilepsy	CALIBER
Read	667E.00	Epilepsy care arrangement	Epilepsy	CALIBER
Read	667F.00	Seizure free >12 months	Epilepsy	CALIBER
Read	667G.00	Epilepsy restricts employment	Epilepsy	CALIBER
Read	667H.00	Epilepsy prevents employment	Epilepsy	CALIBER
Read	667J.00	Epilepsy impairs education	Epilepsy	CALIBER
Read	667K.00	Epilepsy limits activities	Epilepsy	CALIBER
Read	667L.00	Epilepsy does not limit activities	Epilepsy	CALIBER
Read	667M.00	Epilepsy management plan given	Epilepsy	CALIBER
Read	667N.00	Epilepsy severity	Epilepsy	CALIBER
Read	667P.00	No seizures on treatment	Epilepsy	CALIBER
Read	667Q.00	1 to 12 seizures a year	Epilepsy	CALIBER
Read	667R.00	2 to 4 seizures a month	Epilepsy	CALIBER
Read	667S.00	1 to 7 seizures a week	Epilepsy	CALIBER
Read	667T.00	Daily seizures	Epilepsy	CALIBER
Read	667V.00	Many seizures a day	Epilepsy	CALIBER

Read	667W.00	Emergency epilepsy treatment since last appointment	Epilepsy	CALIBER
Read	667X.00	No epilepsy drug side effects	Epilepsy	CALIBER
Read	667Z.00	Epilepsy monitoring NOS	Epilepsy	CALIBER
Read	67AF.00	Pregnancy advice for patients with epilepsy	Epilepsy	CALIBER
Read	67IJ000	Pre-conception advice for patients with epilepsy	Epilepsy	CALIBER
Read	8BIF.00	Epilepsy medication review	Epilepsy	CALIBER
Read	9Of3.00	Epilepsy monitoring verbal invite	Epilepsy	CALIBER
Read	9Of4.00	Epilepsy monitoring telephone invite	Epilepsy	CALIBER
Read	9Of5.00	Epilepsy monitoring call first letter	Epilepsy	CALIBER
Read	9Of6.00	Epilepsy monitoring call second letter	Epilepsy	CALIBER
Read	9Of7.00	Epilepsy monitoring call third letter	Epilepsy	CALIBER
Read	Eu05212	[X]Schizophrenia-like psychosis in epilepsy	Epilepsy	CALIBER
Read	Eu05y11	[X]Epileptic psychosis NOS	Epilepsy	CALIBER
Read	Eu06013	[X]Limbic epilepsy personality	Epilepsy	CALIBER
Read	Eu80300	[X]Acquired aphasia with epilepsy [Landau - Kleffner]	Epilepsy	CALIBER
Read	F132100	Progressive myoclonic epilepsy	Epilepsy	CALIBER
Read	F132111	Unverricht - Lundborg disease	Epilepsy	CALIBER
Read	F132200	Myoclonic encephalopathy	Epilepsy	CALIBER
Read	F142200	Dyssynergia cerebellaris myoclonica	Epilepsy	CALIBER
Read	F250000	Petit mal (minor) epilepsy	Epilepsy	CALIBER
Read	F250011	Epileptic absences	Epilepsy	CALIBER
Read	F250100	Pykno-epilepsy	Epilepsy	CALIBER
Read	F250200	Epileptic seizures - atonic	Epilepsy	CALIBER
Read	F250300	Epileptic seizures - akinetic	Epilepsy	CALIBER
Read	F250400	Juvenile absence epilepsy	Epilepsy	CALIBER
Read	F250500	Lennox-Gastaut syndrome	Epilepsy	CALIBER
Read	F250.00	Generalised nonconvulsive epilepsy	Epilepsy	CALIBER
Read	F250y00	Other specified generalised nonconvulsive epilepsy	Epilepsy	CALIBER
Read	F250z00	Generalised nonconvulsive epilepsy NOS	Epilepsy	CALIBER
Read	F251000	Grand mal (major) epilepsy	Epilepsy	CALIBER
Read	F251011	Tonic-clonic epilepsy	Epilepsy	CALIBER
Read	F251100	Neonatal myoclonic epilepsy	Epilepsy	CALIBER
Read	F251111	Otohara syndrome	Epilepsy	CALIBER
Read	F251200	Epileptic seizures - clonic	Epilepsy	CALIBER
Read	F251300	Epileptic seizures - myoclonic	Epilepsy	CALIBER
Read	F251400	Epileptic seizures - tonic	Epilepsy	CALIBER
Read	F251500	Tonic-clonic epilepsy	Epilepsy	CALIBER
Read	F251.00	Generalised convulsive epilepsy	Epilepsy	CALIBER

Read	F251y00	Other specified generalised convulsive epilepsy	Epilepsy	CALIBER
Read	F251z00	Generalised convulsive epilepsy NOS	Epilepsy	CALIBER
Read	F252.00	Petit mal status	Epilepsy	CALIBER
Read	F253.00	Grand mal status	Epilepsy	CALIBER
Read	F253.11	Status epilepticus	Epilepsy	CALIBER
Read	F254000	Temporal lobe epilepsy	Epilepsy	CALIBER
Read	F254100	Psychomotor epilepsy	Epilepsy	CALIBER
Read	F254200	Psychosensory epilepsy	Epilepsy	CALIBER
Read	F254300	Limbic system epilepsy	Epilepsy	CALIBER
Read	F254400	Epileptic automatism	Epilepsy	CALIBER
Read	F254500	Complex partial epileptic seizure	Epilepsy	CALIBER
Read	F254.00	Partial epilepsy with impairment of consciousness	Epilepsy	CALIBER
Read	F254z00	Partial epilepsy with impairment of consciousness NOS	Epilepsy	CALIBER
Read	F255000	Jacksonian, focal or motor epilepsy	Epilepsy	CALIBER
Read	F255011	Focal epilepsy	Epilepsy	CALIBER
Read	F255012	Motor epilepsy	Epilepsy	CALIBER
Read	F255100	Sensory induced epilepsy	Epilepsy	CALIBER
Read	F255200	Somatosensory epilepsy	Epilepsy	CALIBER
Read	F255300	Visceral reflex epilepsy	Epilepsy	CALIBER
Read	F255311	Partial epilepsy with autonomic symptoms	Epilepsy	CALIBER
Read	F255400	Visual reflex epilepsy	Epilepsy	CALIBER
Read	F255500	Unilateral epilepsy	Epilepsy	CALIBER
Read	F255600	Simple partial epileptic seizure	Epilepsy	CALIBER
Read	F255.00	Partial epilepsy without impairment of consciousness	Epilepsy	CALIBER
Read	F255y00	Partial epilepsy without impairment of consciousness OS	Epilepsy	CALIBER
Read	F255z00	Partial epilepsy without impairment of consciousness NOS	Epilepsy	CALIBER
Read	F256000	Hypsarrhythmia	Epilepsy	CALIBER
Read	F256100	Salaam attacks	Epilepsy	CALIBER
Read	F256.00	Infantile spasms	Epilepsy	CALIBER
Read	F256.11	Lightning spasms	Epilepsy	CALIBER
Read	F256.12	West syndrome	Epilepsy	CALIBER
Read	F256z00	Infantile spasms NOS	Epilepsy	CALIBER
Read	F257.00	Kojevnikov's epilepsy	Epilepsy	CALIBER
Read	F258.00	Post-ictal state	Epilepsy	CALIBER
Read	F259.00	Early infant epileptic encephalopathy wth suppression bursts	Epilepsy	CALIBER
Read	F259.11	Ohtahara syndrome	Epilepsy	CALIBER

Read	F25A.00	Juvenile myoclonic epilepsy	Epilepsy	CALIBER
Read	F25B.00	Alcohol-induced epilepsy	Epilepsy	CALIBER
Read	F25C.00	Drug-induced epilepsy	Epilepsy	CALIBER
Read	F25D.00	Menstrual epilepsy	Epilepsy	CALIBER
Read	F25E.00	Stress-induced epilepsy	Epilepsy	CALIBER
Read	F25..00	Epilepsy	Epilepsy	CALIBER
Read	F25F.00	Photosensitive epilepsy	Epilepsy	CALIBER
Read	F25G.00	Severe myoclonic epilepsy in infancy	Epilepsy	CALIBER
Read	F25G.11	Dravet syndrome	Epilepsy	CALIBER
Read	F25X.00	Status epilepticus, unspecified	Epilepsy	CALIBER
Read	F25y000	Cursive (running) epilepsy	Epilepsy	CALIBER
Read	F25y100	Gelastic epilepsy	Epilepsy	CALIBER
Read	F25y200	Locl-rlt(foc)(part)idiop epilep&epilptic syn seiz locl onset	Epilepsy	CALIBER
Read	F25y300	Complex partial status epilepticus	Epilepsy	CALIBER
Read	F25y400	Benign Rolandic epilepsy	Epilepsy	CALIBER
Read	F25y500	Panayiotopoulos syndrome	Epilepsy	CALIBER
Read	F25y.00	Other forms of epilepsy	Epilepsy	CALIBER
Read	F25yz00	Other forms of epilepsy NOS	Epilepsy	CALIBER
Read	F25z.00	Epilepsy NOS	Epilepsy	CALIBER
Read	F25z.11	Fit (in known epileptic) NOS	Epilepsy	CALIBER
Read	Fyu5000	[X]Other generalized epilepsy and epileptic syndromes	Epilepsy	CALIBER
Read	Fyu5100	[X]Other epilepsy	Epilepsy	CALIBER
Read	Fyu5200	[X]Other status epilepticus	Epilepsy	CALIBER
Read	Fyu5900	[X]Status epilepticus, unspecified	Epilepsy	CALIBER
ICD10	G40	Epilepsy	Epilepsy	CALIBER
ICD10	G41	Status epilepticus	Epilepsy	CALIBER
Read	7259000	Needling of bleb following glaucoma surgery	Glaucoma	CALIBER
Read	7259100	Injection of bleb following glaucoma surgery	Glaucoma	CALIBER
Read	7259200	Revision of bleb NEC following glaucoma surgery	Glaucoma	CALIBER
Read	7259300	Removal of releasable suture following glaucoma surgery	Glaucoma	CALIBER
Read	7259400	Laser suture lysis following glaucoma surgery	Glaucoma	CALIBER
Read	7259	Operations following glaucoma surgery	Glaucoma	CALIBER
Read	7259y00	Other specified operations following glaucoma surgery	Glaucoma	CALIBER
Read	7259z00	Operations following glaucoma surgery NOS	Glaucoma	CALIBER
Read	7275	Pan retinal photocoagulation for glaucoma	Glaucoma	CALIBER
Read	F404211	Glaucoma - absolute	Glaucoma	CALIBER

Read	F450100	Open angle glaucoma with borderline intraocular pressure	Glaucoma	CALIBER
Read	F451000	Unspecified open-angle glaucoma	Glaucoma	CALIBER
Read	F451100	Primary open-angle glaucoma	Glaucoma	CALIBER
Read	F451111	Simple chronic glaucoma	Glaucoma	CALIBER
Read	F451200	Low tension glaucoma	Glaucoma	CALIBER
Read	F451211	Normal pressure glaucoma	Glaucoma	CALIBER
Read	F451500	Open-angle glaucoma residual stage	Glaucoma	CALIBER
Read	F451.00	Open-angle glaucoma	Glaucoma	CALIBER
Read	F451z00	Open-angle glaucoma NOS	Glaucoma	CALIBER
Read	F452000	Unspecified primary angle-closure glaucoma	Glaucoma	CALIBER
Read	F452100	Intermittent primary angle-closure glaucoma	Glaucoma	CALIBER
Read	F452200	Acute primary angle-closure glaucoma	Glaucoma	CALIBER
Read	F452300	Chronic primary angle-closure glaucoma	Glaucoma	CALIBER
Read	F452400	Primary angle-closure glaucoma residual stage	Glaucoma	CALIBER
Read	F452500	Plateau iris	Glaucoma	CALIBER
Read	F452.00	Primary angle-closure glaucoma	Glaucoma	CALIBER
Read	F452.11	Closed angle glaucoma	Glaucoma	CALIBER
Read	F452z00	Primary angle-closure glaucoma NOS	Glaucoma	CALIBER
Read	F45..00	Glaucoma	Glaucoma	CALIBER
Read	F45y200	Low tension glaucoma	Glaucoma	CALIBER
Read	F45y.00	Other specified forms of glaucoma	Glaucoma	CALIBER
Read	F45yz00	Other specified glaucoma NOS	Glaucoma	CALIBER
Read	F45z.00	Glaucoma NOS	Glaucoma	CALIBER
Read	F463100	Glaucomatous subcapsular flecks	Glaucoma	CALIBER
Read	F4H1400	Optic disc glaucomatous atrophy	Glaucoma	CALIBER
Read	FyuG.00	[X]Glaucoma	Glaucoma	CALIBER
ICD10	H40.1	Primary open-angle glaucoma	Glaucoma	CALIBER
ICD10	H40.2	Primary angle-closure glaucoma	Glaucoma	CALIBER
ICD10	H40.9	Glaucoma, unspecified	Glaucoma	CALIBER
OPCS	C60.1	Trabeculectomy	Glaucoma	CALIBER
OPCS	C60.2	Inclusion of iris	Glaucoma	CALIBER
OPCS	C60.3	Fixation of iris	Glaucoma	CALIBER
OPCS	C60.4	Iridoplasty NEC	Glaucoma	CALIBER
OPCS	C60.5	Insertion of tube into anterior chamber of eye to assist drainage of aqueous humour	Glaucoma	CALIBER
OPCS	C60.6	Viscocanulostomy	Glaucoma	CALIBER
OPCS	C60.8	Other specified filtering operations on iris	Glaucoma	CALIBER
OPCS	C60.9	Unspecified filtering operations on iris	Glaucoma	CALIBER
OPCS	C61.1	Laser trabeculoplasty	Glaucoma	CALIBER

OPCS	C61.2	Trabeculotomy	Glaucoma	CALIBER
OPCS	C61.3	Goniotomy	Glaucoma	CALIBER
OPCS	C61.4	Goniopuncture	Glaucoma	CALIBER
OPCS	C61.5	Viscogonioplasty	Glaucoma	CALIBER
OPCS	C61.8	Other specified other operations on trabecular meshwork of eye	Glaucoma	CALIBER
OPCS	C61.9	Unspecified other operations on trabecular meshwork of eye	Glaucoma	CALIBER
OPCS	C62.1	Iridosclerotomy	Glaucoma	CALIBER
OPCS	C62.2	Surgical iridotomy	Glaucoma	CALIBER
OPCS	C62.3	Laser iridotomy	Glaucoma	CALIBER
OPCS	C62.4	Correction iridodialysis NEC	Glaucoma	CALIBER
OPCS	C62.8	Other specified incision of iris	Glaucoma	CALIBER
OPCS	C62.9	Unspecified incision of iris	Glaucoma	CALIBER
Read	14A6.00	H/O: heart failure	Heart failure	CALIBER
Read	14AM.00	H/O: Heart failure in last year	Heart failure	CALIBER
Read	1O1..00	Heart failure confirmed	Heart failure	CALIBER
Read	388D.00	New York Heart Assoc classification heart failure symptoms	Heart failure	CALIBER
Read	661M500	Heart failure self-management plan agreed	Heart failure	CALIBER
Read	662f.00	New York Heart Association classification - class I	Heart failure	CALIBER
Read	662g.00	New York Heart Association classification - class II	Heart failure	CALIBER
Read	662h.00	New York Heart Association classification - class III	Heart failure	CALIBER
Read	662i.00	New York Heart Association classification - class IV	Heart failure	CALIBER
Read	662p.00	Heart failure 6 month review	Heart failure	CALIBER
Read	662T.00	Congestive heart failure monitoring	Heart failure	CALIBER
Read	662W.00	Heart failure annual review	Heart failure	CALIBER
Read	679W100	Education about deteriorating heart failure	Heart failure	CALIBER
Read	679X.00	Heart failure education	Heart failure	CALIBER
Read	8B29.00	Cardiac failure therapy	Heart failure	CALIBER
Read	8CeC.00	Preferred place of care for next exacerbation heart failure	Heart failure	CALIBER
Read	8CL3.00	Heart failure care plan discussed with patient	Heart failure	CALIBER
Read	8CMK.00	Has heart failure management plan	Heart failure	CALIBER
Read	8CMW800	Heart failure clinical pathway	Heart failure	CALIBER
Read	8H2S.00	Admit heart failure emergency	Heart failure	CALIBER
Read	8HBE.00	Heart failure follow-up	Heart failure	CALIBER
Read	8HHz.00	Referral to heart failure exercise programme	Heart failure	CALIBER

Read	8Hk0.00	Referred to heart failure education group	Heart failure	CALIBER
Read	9h11.00	Excepted from LVD quality indicators: Patient unsuitable	Heart failure	CALIBER
Read	9h12.00	Excepted from LVD quality indicators: Informed dissent	Heart failure	CALIBER
Read	9h1..00	Exception reporting: LVD quality indicators	Heart failure	CALIBER
Read	9hH0.00	Excepted heart failure quality indicators: Patient unsuitabl	Heart failure	CALIBER
Read	9hH1.00	Excepted heart failure quality indicators: Informed dissent	Heart failure	CALIBER
Read	9hH..00	Exception reporting: heart failure quality indicators	Heart failure	CALIBER
Read	9N2p.00	Seen by community heart failure nurse	Heart failure	CALIBER
Read	9N6T.00	Referred by heart failure nurse specialist	Heart failure	CALIBER
Read	9On0.00	Left ventricular dysfunction monitoring first letter	Heart failure	CALIBER
Read	9On1.00	Left ventricular dysfunction monitoring second letter	Heart failure	CALIBER
Read	9On2.00	Left ventricular dysfunction monitoring third letter	Heart failure	CALIBER
Read	9On3.00	Left ventricular dysfunction monitoring verbal invite	Heart failure	CALIBER
Read	9On4.00	Left ventricular dysfunction monitoring telephone invite	Heart failure	CALIBER
Read	9On..00	Left ventricular dysfunction monitoring administration	Heart failure	CALIBER
Read	9Or0.00	Heart failure review completed	Heart failure	CALIBER
Read	9Or1.00	Heart failure monitoring telephone invite	Heart failure	CALIBER
Read	9Or2.00	Heart failure monitoring verbal invite	Heart failure	CALIBER
Read	9Or3.00	Heart failure monitoring first letter	Heart failure	CALIBER
Read	9Or4.00	Heart failure monitoring second letter	Heart failure	CALIBER
Read	9Or5.00	Heart failure monitoring third letter	Heart failure	CALIBER
Read	9Or..00	Heart failure monitoring administration	Heart failure	CALIBER
Read	G1yz100	Rheumatic left ventricular failure	Heart failure	CALIBER
Read	G210100	Malignant hypertensive heart disease with CCF	Heart failure	CALIBER
Read	G211100	Benign hypertensive heart disease with CCF	Heart failure	CALIBER
Read	G21z100	Hypertensive heart disease NOS with CCF	Heart failure	CALIBER
Read	G232.00	Hypertensive heart&renal dis wth (congestive) heart failure	Heart failure	CALIBER
Read	G234.00	Hyperten heart&renal dis+both(congestv)heart and renal fail	Heart failure	CALIBER
Read	G400.00	Acute cor pulmonale	Heart failure	CALIBER
Read	G41z.11	Chronic cor pulmonale	Heart failure	CALIBER
Read	G554000	Congestive cardiomyopathy	Heart failure	CALIBER

Read	G554011	Congestive obstructive cardiomyopathy	Heart failure	CALIBER
Read	G580000	Acute congestive heart failure	Heart failure	CALIBER
Read	G580100	Chronic congestive heart failure	Heart failure	CALIBER
Read	G580200	Decompensated cardiac failure	Heart failure	CALIBER
Read	G580300	Compensated cardiac failure	Heart failure	CALIBER
Read	G580400	Congestive heart failure due to valvular disease	Heart failure	CALIBER
Read	G580.00	Congestive heart failure	Heart failure	CALIBER
Read	G580.11	Congestive cardiac failure	Heart failure	CALIBER
Read	G580.12	Right heart failure	Heart failure	CALIBER
Read	G580.13	Right ventricular failure	Heart failure	CALIBER
Read	G580.14	Biventricular failure	Heart failure	CALIBER
Read	G581000	Acute left ventricular failure	Heart failure	CALIBER
Read	G581.00	Left ventricular failure	Heart failure	CALIBER
Read	G581.11	Asthma - cardiac	Heart failure	CALIBER
Read	G581.13	Impaired left ventricular function	Heart failure	CALIBER
Read	G582.00	Acute heart failure	Heart failure	CALIBER
Read	G584.00	Right ventricular failure	Heart failure	CALIBER
Read	G58..00	Heart failure	Heart failure	CALIBER
Read	G58..11	Cardiac failure	Heart failure	CALIBER
Read	G58z.00	Heart failure NOS	Heart failure	CALIBER
Read	G58z.12	Cardiac failure NOS	Heart failure	CALIBER
Read	G5yy900	Left ventricular systolic dysfunction	Heart failure	CALIBER
Read	G5yyA00	Left ventricular diastolic dysfunction	Heart failure	CALIBER
Read	ZRad.00	New York Heart Assoc classification heart failure symptoms	Heart failure	CALIBER
ICD10	I11.0	Hypertensive heart disease with (congestive) heart failure	Heart failure	CALIBER
ICD10	I13.0	Hypertensive heart and renal disease with (congestive) heart failure	Heart failure	CALIBER
ICD10	I13.2	Hypertensive heart and renal disease with both (congestive) heart failure and renal failure	Heart failure	CALIBER
ICD10	I50	Heart failure	Heart failure	CALIBER
Read	14A2.00	H/O: hypertension	Hypertension	CALIBER
Read	2126100	Hypertension resolved	Hypertension	CALIBER
Read	212K.00	Hypertension resolved	Hypertension	CALIBER
Read	9OI9.00	Hypertens.monitor deleted	Hypertension	CALIBER
Read	6624	Borderline hyperten:yearly obs	Hypertension	CALIBER
Read	6627	Good hypertension control	Hypertension	CALIBER
Read	6628	Poor hypertension control	Hypertension	CALIBER
Read	662F.00	Hypertension treatm. started	Hypertension	CALIBER
Read	662G.00	Hypertensive treatm.changed	Hypertension	CALIBER
Read	662O.00	On treatment for hypertension	Hypertension	CALIBER

Read	662b.00	Moderate hypertension control	Hypertension	CALIBER
Read	662c.00	Hypertension six month review	Hypertension	CALIBER
Read	662d.00	Hypertension annual review	Hypertension	CALIBER
Read	662r.00	Trial withdrawal of antihypertensive therapy	Hypertension	CALIBER
Read	7Q01.00	High cost hypertension drugs	Hypertension	CALIBER
Read	8B26.00	Antihypertensive therapy	Hypertension	CALIBER
Read	8BL0.00	Patient on maximal tolerated antihypertensive therapy	Hypertension	CALIBER
Read	8I3N.00	Hypertension treatment refused	Hypertension	CALIBER
Read	F404200	Blind hypertensive eye	Hypertension	CALIBER
Read	F421300	Hypertensive retinopathy	Hypertension	CALIBER
Read	G2...00	Hypertensive disease	Hypertension	CALIBER
Read	G2...11	BP - hypertensive disease	Hypertension	CALIBER
Read	G20..00	Essential hypertension	Hypertension	CALIBER
Read	G200.00	Malignant essential hypertension	Hypertension	CALIBER
Read	G201.00	Benign essential hypertension	Hypertension	CALIBER
Read	G202.00	Systolic hypertension	Hypertension	CALIBER
Read	G203.00	Diastolic hypertension	Hypertension	CALIBER
Read	G20z.00	Essential hypertension NOS	Hypertension	CALIBER
Read	G20z.11	Hypertension NOS	Hypertension	CALIBER
Read	G21..00	Hypertensive heart disease	Hypertension	CALIBER
Read	G210.00	Malignant hypertensive heart disease	Hypertension	CALIBER
Read	G210000	Malignant hypertensive heart disease without CCF	Hypertension	CALIBER
Read	G210100	Malignant hypertensive heart disease with CCF	Hypertension	CALIBER
Read	G211.00	Benign hypertensive heart disease	Hypertension	CALIBER
Read	G211000	Benign hypertensive heart disease without CCF	Hypertension	CALIBER
Read	G211100	Benign hypertensive heart disease with CCF	Hypertension	CALIBER
Read	G21z.00	Hypertensive heart disease NOS	Hypertension	CALIBER
Read	G21z000	Hypertensive heart disease NOS without CCF	Hypertension	CALIBER
Read	G21z011	Cardiomegaly - hypertensive	Hypertension	CALIBER
Read	G21z100	Hypertensive heart disease NOS with CCF	Hypertension	CALIBER
Read	G21zz00	Hypertensive heart disease NOS	Hypertension	CALIBER
Read	G22..00	Hypertensive renal disease	Hypertension	CALIBER
Read	G220.00	Malignant hypertensive renal disease	Hypertension	CALIBER
Read	G221.00	Benign hypertensive renal disease	Hypertension	CALIBER
Read	G222.00	Hypertensive renal disease with renal failure	Hypertension	CALIBER
Read	G22z.00	Hypertensive renal disease NOS	Hypertension	CALIBER

Read	G22z.11	Renal hypertension	Hypertension	CALIBER
Read	G23..00	Hypertensive heart and renal disease	Hypertension	CALIBER
Read	G230.00	Malignant hypertensive heart and renal disease	Hypertension	CALIBER
Read	G231.00	Benign hypertensive heart and renal disease	Hypertension	CALIBER
Read	G232.00	Hypertensive heart&renal dis wth (congestive) heart failure	Hypertension	CALIBER
Read	G233.00	Hypertensive heart and renal disease with renal failure	Hypertension	CALIBER
Read	G234.00	Hyperten heart&renal dis+both(congestv)heart and renal fail	Hypertension	CALIBER
Read	G23z.00	Hypertensive heart and renal disease NOS	Hypertension	CALIBER
Read	G2y..00	Other specified hypertensive disease	Hypertension	CALIBER
Read	G2z..00	Hypertensive disease NOS	Hypertension	CALIBER
Read	G672.00	Hypertensive encephalopathy	Hypertension	CALIBER
Read	G672.11	Hypertensive crisis	Hypertension	CALIBER
Read	Gyu2.00	[X]Hypertensive diseases	Hypertension	CALIBER
Read	L122.00	Other pre-existing hypertension in preg/childbirth/puerp	Hypertension	CALIBER
Read	L122000	Other pre-existing hypertension in preg/childb/puerp unspec	Hypertension	CALIBER
Read	L122100	Other pre-existing hypertension in preg/childb/puerp - deliv	Hypertension	CALIBER
Read	L122300	Other pre-exist hypertension in preg/childb/puerp-not deliv	Hypertension	CALIBER
Read	L122z00	Other pre-existing hypertension in preg/childb/puerp NOS	Hypertension	CALIBER
Read	L127.00	Pre-eclampsia or eclampsia with pre-existing hypertension	Hypertension	CALIBER
Read	L127z00	Pre-eclampsia or eclampsia + pre-existing hypertension NOS	Hypertension	CALIBER
Read	L128.00	Pre-exist hypertension compl preg childbirth and puerperium	Hypertension	CALIBER
Read	L128000	Pre-exist hyperten heart dis compl preg childbth+puerperium	Hypertension	CALIBER
Read	L128200	Pre-exist 2ndry hypertens comp preg childbth and puerperium	Hypertension	CALIBER
Read	TJC7.00	Adverse reaction to other antihypertensives	Hypertension	CALIBER
Read	TJC7z00	Adverse reaction to antihypertensives NOS	Hypertension	CALIBER
Read	U60C500	[X]Oth antihyperten drug caus advers eff in therap use, NEC	Hypertension	CALIBER
Read	U60C511	[X] Adverse reaction to other antihypertensives	Hypertension	CALIBER
Read	U60C51A	[X] Adverse reaction to antihypertensives NOS	Hypertension	CALIBER
Read	6146200	Hypertension induced by oral contraceptive pill	Hypertension	CALIBER

Read	G24..00	Secondary hypertension	Hypertension	CALIBER
Read	G240.00	Secondary malignant hypertension	Hypertension	CALIBER
Read	G240000	Secondary malignant renovascular hypertension	Hypertension	CALIBER
Read	G240z00	Secondary malignant hypertension NOS	Hypertension	CALIBER
Read	G241.00	Secondary benign hypertension	Hypertension	CALIBER
Read	G241000	Secondary benign renovascular hypertension	Hypertension	CALIBER
Read	G241z00	Secondary benign hypertension NOS	Hypertension	CALIBER
Read	G244.00	Hypertension secondary to endocrine disorders	Hypertension	CALIBER
Read	G24z.00	Secondary hypertension NOS	Hypertension	CALIBER
Read	G24z000	Secondary renovascular hypertension NOS	Hypertension	CALIBER
Read	G24z100	Hypertension secondary to drug	Hypertension	CALIBER
Read	G24zz00	Secondary hypertension NOS	Hypertension	CALIBER
Read	Gyu2100	[X]Hypertension secondary to other renal disorders	Hypertension	CALIBER
ICD10	I10	Essential (primary) hypertension	Hypertension	CALIBER
ICD10	I11	Hypertensive heart disease	Hypertension	CALIBER
ICD10	I12	Hypertensive renal disease	Hypertension	CALIBER
ICD10	I13	Hypertensive heart and renal disease	Hypertension	CALIBER
ICD10	I15	Secondary hypertension	Hypertension	CALIBER
Read	1432	H/O: hypothyroidism	Hypothyroidism	CALIBER
Read	66BB.00	Hypothyroidism annual review	Hypothyroidism	CALIBER
Read	8CR5.00	Hypothyroidism clinical management plan	Hypothyroidism	CALIBER
Read	9Oj0.00	Hypothyroidism monitoring first letter	Hypothyroidism	CALIBER
Read	9Oj1.00	Hypothyroidism monitoring second letter	Hypothyroidism	CALIBER
Read	9Oj2.00	Hypothyroidism monitoring third letter	Hypothyroidism	CALIBER
Read	9Oj3.00	Hypothyroidism monitoring verbal invite	Hypothyroidism	CALIBER
Read	9Oj4.00	Hypothyroidism monitoring telephone invitation	Hypothyroidism	CALIBER
Read	9Oj..00	Hypothyroidism monitoring administration	Hypothyroidism	CALIBER
Read	C040.00	Postsurgical hypothyroidism	Hypothyroidism	CALIBER
Read	C040.11	Post ablative hypothyroidism	Hypothyroidism	CALIBER
Read	C041000	Irradiation hypothyroidism	Hypothyroidism	CALIBER
Read	C041.00	Other postablative hypothyroidism	Hypothyroidism	CALIBER
Read	C041z00	Postablative hypothyroidism NOS	Hypothyroidism	CALIBER
Read	C043.00	Other iatrogenic hypothyroidism	Hypothyroidism	CALIBER
Read	C043z00	Iatrogenic hypothyroidism NOS	Hypothyroidism	CALIBER
Read	C046.00	Autoimmune myxoedema	Hypothyroidism	CALIBER
Read	C04..00	Acquired hypothyroidism	Hypothyroidism	CALIBER
Read	C04..11	Myxoedema	Hypothyroidism	CALIBER
Read	C04..12	Thyroid deficiency	Hypothyroidism	CALIBER

Read	C04..13	Hypothyroidism	Hypothyroidism	CALIBER
Read	C04y.00	Other acquired hypothyroidism	Hypothyroidism	CALIBER
Read	C04z000	Premature puberty due to hypothyroidism	Hypothyroidism	CALIBER
Read	C04z100	Myxoedema coma	Hypothyroidism	CALIBER
Read	C04z.00	Hypothyroidism NOS	Hypothyroidism	CALIBER
Read	C04z.11	Pretibial myxoedema - hypothyroid	Hypothyroidism	CALIBER
Read	C04z.12	Thyroid insufficiency	Hypothyroidism	CALIBER
Read	C04z.13	Hypothyroid goitre, acquired	Hypothyroidism	CALIBER
Read	C06y100	Thyroid atrophy	Hypothyroidism	CALIBER
Read	C134300	TSH - thyroid-stimulating hormone deficiency	Hypothyroidism	CALIBER
Read	Cyu1100	[X]Other sp cified hypothyroidism	Hypothyroidism	CALIBER
Read	F11x500	Cerebral degeneration due to myxoedema	Hypothyroidism	CALIBER
Read	F381400	Myasthenic syndrome due to hypothyroidism	Hypothyroidism	CALIBER
Read	F395300	Myopathy due to myxoedema	Hypothyroidism	CALIBER
ICD10	E03.5	Myxoedema coma	Hypothyroidism	CALIBER
ICD10	E03.8	Other specified hypothyroidism	Hypothyroidism	CALIBER
ICD10	E03.9	Hypothyroidism, unspecified	Hypothyroidism	CALIBER
OPCS	G51.4	Bypass of duodenum by anastomosis of duodenum to colon (Clean-contaminated)	Intestinal anastomosis	OPCS Classification of Interventions and Procedures
OPCS	G58.3	Total jejunectomy and anastomosis of duodenum to colon*	Intestinal anastomosis	OPCS Classification of Interventions and Procedures
OPCS	G58.5	Partial jejunectomy and anastomosis of duodenum to colon*	Intestinal anastomosis	OPCS Classification of Interventions and Procedures
OPCS	G61.3	Bypass of jejunum by anastomosis of jejunum to colon (Clean-contaminated)	Intestinal anastomosis	OPCS Classification of Interventions and Procedures
OPCS	G69.4	Ileectomy and anastomosis of ileum to colon (Clean-contaminated)	Intestinal anastomosis	OPCS Classification of Interventions

				and Procedures
OPCS	G71.3	Bypass of ileum by anastomosis of ileum to caecum (Clean-contaminated)	Intestinal anastomosis	OPCS Classification of Interventions and Procedures
OPCS	G71.4	Bypass of ileum by anastomosis of ileum to transverse colon (Clean-contaminated)	Intestinal anastomosis	OPCS Classification of Interventions and Procedures
OPCS	G71.5	Bypass of ileum by anastomosis of ileum to colon not elsewhere classified (Cleancontaminated)	Intestinal anastomosis	OPCS Classification of Interventions and Procedures
OPCS	G72.1	Anastomosis of ileum to caecum	Intestinal anastomosis	OPCS Classification of Interventions and Procedures
OPCS	G72.2	Anastomosis of ileum to transverse colon	Intestinal anastomosis	OPCS Classification of Interventions and Procedures
OPCS	G72.3	Anastomosis of ileum to colon not elsewhere classified	Intestinal anastomosis	OPCS Classification of Interventions and Procedures
OPCS	G72.4	Anastomosis of ileum to rectum	Intestinal anastomosis	OPCS Classification of Interventions and Procedures
OPCS	G72.5	Anastomosis of ileum to anus and creation of pouch however further qualified	Intestinal anastomosis	OPCS Classification of Interventions and Procedures

OPCS	H04.2	Panproctocolectomy and anastomosis of ileum to anus and creation of pouch however further qualified	Intestinal anastomosis	OPCS Classification of Interventions and Procedures
OPCS	H04.3	Panproctocolectomy and anastomosis of ileum to anus not elsewhere classified	Intestinal anastomosis	OPCS Classification of Interventions and Procedures
OPCS	H05.1	Total colectomy and anastomosis of ileum to rectum	Intestinal anastomosis	OPCS Classification of Interventions and Procedures
OPCS	H06.1	Extended right hemicolectomy and end to end anastomosis	Intestinal anastomosis	OPCS Classification of Interventions and Procedures
OPCS	H06.2	Extended right hemicolectomy and anastomosis of ileum to colon	Intestinal anastomosis	OPCS Classification of Interventions and Procedures
OPCS	H06.3	Extended right hemicolectomy and anastomosis not elsewhere classified	Intestinal anastomosis	OPCS Classification of Interventions and Procedures
OPCS	H06.5	Extended right hemicolectomy and end to side anastomosis	Intestinal anastomosis	OPCS Classification of Interventions and Procedures
OPCS	H07.1	Right hemicolectomy and end to end anastomosis of ileum to colon	Intestinal anastomosis	OPCS Classification of Interventions and Procedures
OPCS	H07.2	Right hemicolectomy and side to side anastomosis of ileum to transverse colon	Intestinal anastomosis	OPCS Classification

				of Interventions and Procedures
OPCS	H07.3	Right hemicolectomy and anastomosis not elsewhere classified	Intestinal anastomosis	OPCS Classification of Interventions and Procedures
OPCS	H07.5	Right hemicolectomy and end to side anastomosis	Intestinal anastomosis	OPCS Classification of Interventions and Procedures
OPCS	H08.1	Transverse colectomy and end to end anastomosis	Intestinal anastomosis	OPCS Classification of Interventions and Procedures
OPCS	H08.2	Transverse colectomy and anastomosis of ileum to colon	Intestinal anastomosis	OPCS Classification of Interventions and Procedures
OPCS	H08.3	Transverse colectomy and anastomosis not elsewhere classified	Intestinal anastomosis	OPCS Classification of Interventions and Procedures
OPCS	H08.6	Transverse colectomy and end to side anastomosis	Intestinal anastomosis	OPCS Classification of Interventions and Procedures
OPCS	H09.1	Left hemicolectomy and end to end anastomosis of colon to rectum	Intestinal anastomosis	OPCS Classification of Interventions and Procedures
OPCS	H09.2	Left hemicolectomy and end to end anastomosis of colon to colon	Intestinal anastomosis	OPCS Classification of Interventions

				and Procedures
OPCS	H09.3	Left hemicolectomy and anastomosis not elsewhere classified	Intestinal anastomosis	OPCS Classification of Interventions and Procedures
OPCS	H09.6	Left hemicolectomy and end to side anastomosis	Intestinal anastomosis	OPCS Classification of Interventions and Procedures
OPCS	H10.1	Sigmoid colectomy and end to end anastomosis of ileum to rectum	Intestinal anastomosis	OPCS Classification of Interventions and Procedures
OPCS	H10.2	Sigmoid colectomy and anastomosis of colon to rectum	Intestinal anastomosis	OPCS Classification of Interventions and Procedures
OPCS	H10.3	Sigmoid colectomy and anastomosis not elsewhere classified	Intestinal anastomosis	OPCS Classification of Interventions and Procedures
OPCS	H10.6	Sigmoid colectomy and end to side anastomosis	Intestinal anastomosis	OPCS Classification of Interventions and Procedures
OPCS	H11.1	Colectomy and end to end anastomosis of colon to colon not elsewhere classified	Intestinal anastomosis	OPCS Classification of Interventions and Procedures
OPCS	H11.2	Colectomy and side to side anastomosis of ileum to colon not elsewhere classified	Intestinal anastomosis	OPCS Classification of Interventions and Procedures

OPCS	H11.3	Colectomy and anastomosis not elsewhere classified	Intestinal anastomosis	OPCS Classification of Interventions and Procedures
OPCS	H11.6	Colectomy and end to side anastomosis NEC	Intestinal anastomosis	OPCS Classification of Interventions and Procedures
OPCS	H13.1	Bypass of colon by anastomosis of ileum to colon	Intestinal anastomosis	OPCS Classification of Interventions and Procedures
OPCS	H13.2	Bypass of colon by anastomosis of caecum to sigmoid colon	Intestinal anastomosis	OPCS Classification of Interventions and Procedures
OPCS	H13.3	Bypass of colon by anastomosis of transverse colon to sigmoid colon	Intestinal anastomosis	OPCS Classification of Interventions and Procedures
OPCS	H13.4	Bypass of colon by anastomosis of transverse colon to rectum	Intestinal anastomosis	OPCS Classification of Interventions and Procedures
OPCS	H13.5	Bypass of colon by anastomosis of colon to rectum not elsewhere classified	Intestinal anastomosis	OPCS Classification of Interventions and Procedures
OPCS	H29.1	Subtotal excision of colon and rectum and creation of colonic pouch and anastomosis of colon to anus	Intestinal anastomosis	OPCS Classification of Interventions and Procedures

OPCS	H29.3	Subtotal excision of colon and creation of colonic pouch and anastomosis of colon to rectum	Intestinal anastomosis	OPCS Classification of Interventions and Procedures
OPCS	H33.2	Proctectomy and anastomosis of colon to anus	Intestinal anastomosis	OPCS Classification of Interventions and Procedures
OPCS	H33.3	Anterior resection of rectum and anastomosis of colon to rectum using staples	Intestinal anastomosis	OPCS Classification of Interventions and Procedures
OPCS	H33.4	Anterior resection of rectum and anastomosis not elsewhere classified	Intestinal anastomosis	OPCS Classification of Interventions and Procedures
Read	7622300	Bypass of duodenum by anastomosis of duodenum to colon	Intestinal anastomosis	Code Browser
Read	7630200	Total jejunectomy and anastomosis of duodenum to colon	Intestinal anastomosis	Code Browser
Read	7633200	Bypass of jejunum by anastomosis of jejunum to colon	Intestinal anastomosis	Code Browser
Read	7640300	Ileectomy and anastomosis of ileum to colon	Intestinal anastomosis	Code Browser
Read	7642200	Bypass of ileum by anastomosis of ileum to caecum	Intestinal anastomosis	Code Browser
Read	7642300	Bypass of ileum by anastomosis of ileum to transverse colon	Intestinal anastomosis	Code Browser
Read	7642400	Bypass of ileum by anastomosis of ileum to colon NEC	Intestinal anastomosis	Code Browser
Read	7643000	Anastomosis of ileum to caecum	Intestinal anastomosis	Code Browser
Read	7643100	Anastomosis of ileum to transverse colon	Intestinal anastomosis	Code Browser
Read	7643200	Anastomosis of ileum to colon NEC	Intestinal anastomosis	Code Browser
Read	7643300	Anastomosis of ileum to rectum	Intestinal anastomosis	Code Browser
Read	7643400	Anastomosis of ileum to anus and creation of pouch HFQ	Intestinal anastomosis	Code Browser
Read	7710200	Panproctocolectomy and anastomosis of ileum to anus NEC	Intestinal anastomosis	Code Browser

Read	7711000	Total colectomy and anastomosis of ileum to rectum	Intestinal anastomosis	Code Browser
Read	7711011	Hampton ileo-rectal anastomosis	Intestinal anastomosis	Code Browser
Read	7712000	Extended right hemicolectomy and end to end anastomosis	Intestinal anastomosis	Code Browser
Read	7712100	Extended right hemicolectomy and anastomosis ileum to colon	Intestinal anastomosis	Code Browser
Read	7712200	Extended right hemicolectomy and anastomosis NEC	Intestinal anastomosis	Code Browser
Read	7712400	Extended right hemicolectomy and end to side anastomosis	Intestinal anastomosis	Code Browser
Read	7713000	Right hemicolectomy+end to end anastomosis of ileum to colon	Intestinal anastomosis	Code Browser
Read	7713200	Right hemicolectomy and anastomosis NEC	Intestinal anastomosis	Code Browser
Read	7713500	Right hemicolectomy and end to side anastomosis	Intestinal anastomosis	Code Browser
Read	7714000	Transverse colectomy and end to end anastomosis	Intestinal anastomosis	Code Browser
Read	7714100	Transverse colectomy and anastomosis of ileum to colon	Intestinal anastomosis	Code Browser
Read	7714200	Transverse colectomy and anastomosis NEC	Intestinal anastomosis	Code Browser
Read	7715000	Left hemicolectomy+end to end anastomosis of colon to rectum	Intestinal anastomosis	Code Browser
Read	7715100	Left hemicolectomy+end to end anastomosis of colon to colon	Intestinal anastomosis	Code Browser
Read	7715200	Left hemicolectomy and anastomosis NEC	Intestinal anastomosis	Code Browser
Read	7716000	Sigmoid colectomy+end to end anastomosis of ileum to rectum	Intestinal anastomosis	Code Browser
Read	7716100	Sigmoid colectomy and anastomosis of colon to rectum	Intestinal anastomosis	Code Browser
Read	7716200	Sigmoid colectomy and anastomosis NEC	Intestinal anastomosis	Code Browser
Read	7716500	Sigmoid colectomy and end to side anastomosis	Intestinal anastomosis	Code Browser
Read	7717000	Colectomy and end to end anastomosis of colon to colon NEC	Intestinal anastomosis	Code Browser
Read	7717100	Colectomy and side to side anastomosis of ileum to colon NEC	Intestinal anastomosis	Code Browser
Read	7717200	Colectomy and anastomosis NEC	Intestinal anastomosis	Code Browser
Read	7717700	Colectomy and end to side anastomosis	Intestinal anastomosis	Code Browser
Read	7719000	Bypass of colon by anastomosis of ileum to colon	Intestinal anastomosis	Code Browser

Read	7719100	Bypass of colon by anastomosis of caecum to sigmoid colon	Intestinal anastomosis	Code Browser
Read	7719200	Bypass colon by anastomosis of transverse to sigmoid colon	Intestinal anastomosis	Code Browser
Read	7719300	Bypass of colon by anastomosis of transverse colon to rectum	Intestinal anastomosis	Code Browser
Read	7719400	Bypass of colon by anastomosis of colon to rectum NEC	Intestinal anastomosis	Code Browser
Read	7721100	Proctectomy and anastomosis of colon to anus	Intestinal anastomosis	Code Browser
Read	7721200	Anterior resection rectum + staple anastomosis colon-rectum	Intestinal anastomosis	Code Browser
Read	7721300	Anterior resection of rectum and anastomosis NEC	Intestinal anastomosis	Code Browser
Read	7725300	Transsphincteric anastomosis of colon to anus	Intestinal anastomosis	Code Browser
Read	7726000	Rectosigmoidectomy and peranal anastomosis	Intestinal anastomosis	Code Browser
Read	7726300	Peranal mucosal proctectomy and endoanal anastomosis	Intestinal anastomosis	Code Browser
Read	7726311	Delorme mucosal proctectomy and endoanal anastomosis	Intestinal anastomosis	Code Browser
Read	7733300	Reanastomosis rectum-anal canal correct cong rectal atresia	Intestinal anastomosis	Code Browser
Read	B630000	Malignant plasma cell neoplasm, extramedullary plasmacytoma	Malignancy	CALIBER
Read	B630100	Solitary myeloma	Malignancy	CALIBER
Read	B630200	Plasmacytoma NOS	Malignancy	CALIBER
Read	B630300	Lambda light chain myeloma	Malignancy	CALIBER
Read	B630400	Solitary plasmacytoma	Malignancy	CALIBER
Read	B630.00	Multiple myeloma	Malignancy	CALIBER
Read	B630.11	Kahler's disease	Malignancy	CALIBER
Read	B630.12	Myelomatosis	Malignancy	CALIBER
Read	B631.00	Plasma cell leukaemia	Malignancy	CALIBER
Read	B63..00	Multiple myeloma and immunoproliferative neoplasms	Malignancy	CALIBER
Read	B63z.00	Immunoproliferative neoplasm or myeloma NOS	Malignancy	CALIBER
Read	B936.11	Myeloma - solitary	Malignancy	CALIBER
Read	B936.12	Plasmacytoma NOS	Malignancy	CALIBER
Read	BBm6.00	[M] Alpha heavy chain disease	Malignancy	CALIBER
Read	BBmE.00	[M] Gamma heavy chain disease	Malignancy	CALIBER
Read	BBmK.00	[M]Waldenstrom's macroglobulinaemia	Malignancy	CALIBER
Read	BBn0.00	[M]Plasma cell myeloma	Malignancy	CALIBER
Read	BBn0.11	[M]Multiple myeloma	Malignancy	CALIBER
Read	BBn0.12	[M]Myeloma NOS	Malignancy	CALIBER

Read	BBn0.13	[M]Myelomatosis	Malignancy	CALIBER
Read	BBn0.14	[M]Plasmacytic myeloma	Malignancy	CALIBER
Read	BBn2.00	[M]Plasmacytoma NOS	Malignancy	CALIBER
Read	BBn2.11	[M]Monostotic myeloma	Malignancy	CALIBER
Read	BBn2.12	[M]Solitary myeloma	Malignancy	CALIBER
Read	BBn3.00	[M]Plasma cell tumour, malignant	Malignancy	CALIBER
Read	BBn..00	[M]Plasma cell tumours	Malignancy	CALIBER
Read	BBnz.00	[M]Plasma cell tumour NOS	Malignancy	CALIBER
Read	BBr3.00	[M]Plasma cell leukaemias	Malignancy	CALIBER
Read	BBr3z00	[M]Plasma cell leukaemia NOS	Malignancy	CALIBER
Read	C333000	Waldenstrom's macroglobulinaemia	Malignancy	CALIBER
Read	C333011	Waldenstrom macroglobulinaemia	Malignancy	CALIBER
Read	C333100	Alpha heavy chain disease	Malignancy	CALIBER
Read	C333200	Gamma heavy chain disease	Malignancy	CALIBER
Read	C333300	Heavy chain disease	Malignancy	CALIBER
Read	C333.00	Macroglobulinaemia	Malignancy	CALIBER
Read	C333z00	Macroglobulinaemia NOS	Malignancy	CALIBER
ICD10	C88.0	Waldenström macroglobulinaemia	Malignancy	CALIBER
ICD10	C88.2	Other heavy chain disease	Malignancy	CALIBER
ICD10	C90	Multiple myeloma and malignant plasma cell neoplasms	Malignancy	CALIBER
Read	B490.00	Malignant neoplasm of trigone of urinary bladder	Malignancy	CALIBER
Read	B491.00	Malignant neoplasm of dome of urinary bladder	Malignancy	CALIBER
Read	B492.00	Malignant neoplasm of lateral wall of urinary bladder	Malignancy	CALIBER
Read	B493.00	Malignant neoplasm of anterior wall of urinary bladder	Malignancy	CALIBER
Read	B494.00	Malignant neoplasm of posterior wall of urinary bladder	Malignancy	CALIBER
Read	B495.00	Malignant neoplasm of bladder neck	Malignancy	CALIBER
Read	B496.00	Malignant neoplasm of ureteric orifice	Malignancy	CALIBER
Read	B497.00	Malignant neoplasm of urachus	Malignancy	CALIBER
Read	B498.00	Local recurrence of malignant tumour of urinary bladder	Malignancy	CALIBER
Read	B49..00	Malignant neoplasm of urinary bladder	Malignancy	CALIBER
Read	B49y000	Malignant neoplasm, overlapping lesion of bladder	Malignancy	CALIBER
Read	B49y.00	Malignant neoplasm of other site of urinary bladder	Malignancy	CALIBER
Read	B49z.00	Malignant neoplasm of urinary bladder NOS	Malignancy	CALIBER
ICD10	C67	Malignant neoplasm of bladder	Malignancy	CALIBER
Read	B300000	Malignant neoplasm of ethmoid bone	Malignancy	CALIBER

Read	B300100	Malignant neoplasm of frontal bone	Malignancy	CALIBER
Read	B300200	Malignant neoplasm of malar bone	Malignancy	CALIBER
Read	B300300	Malignant neoplasm of nasal bone	Malignancy	CALIBER
Read	B300400	Malignant neoplasm of occipital bone	Malignancy	CALIBER
Read	B300500	Malignant neoplasm of orbital bone	Malignancy	CALIBER
Read	B300600	Malignant neoplasm of parietal bone	Malignancy	CALIBER
Read	B300700	Malignant neoplasm of sphenoid bone	Malignancy	CALIBER
Read	B300800	Malignant neoplasm of temporal bone	Malignancy	CALIBER
Read	B300900	Malignant neoplasm of zygomatic bone	Malignancy	CALIBER
Read	B300A00	Malignant neoplasm of maxilla	Malignancy	CALIBER
Read	B300.00	Malignant neoplasm of bones of skull and face	Malignancy	CALIBER
Read	B300B00	Malignant neoplasm of turbinate	Malignancy	CALIBER
Read	B300C00	Malignant neoplasm of vomer	Malignancy	CALIBER
Read	B300z00	Malignant neoplasm of bones of skull and face NOS	Malignancy	CALIBER
Read	B301.00	Malignant neoplasm of mandible	Malignancy	CALIBER
Read	B302000	Malignant neoplasm of cervical vertebra	Malignancy	CALIBER
Read	B302100	Malignant neoplasm of thoracic vertebra	Malignancy	CALIBER
Read	B302200	Malignant neoplasm of lumbar vertebra	Malignancy	CALIBER
Read	B302.00	Malignant neoplasm of vertebral column	Malignancy	CALIBER
Read	B302z00	Malignant neoplasm of vertebral column NOS	Malignancy	CALIBER
Read	B303000	Malignant neoplasm of rib	Malignancy	CALIBER
Read	B303100	Malignant neoplasm of sternum	Malignancy	CALIBER
Read	B303200	Malignant neoplasm of clavicle	Malignancy	CALIBER
Read	B303300	Malignant neoplasm of costal cartilage	Malignancy	CALIBER
Read	B303400	Malignant neoplasm of costo-vertebral joint	Malignancy	CALIBER
Read	B303500	Malignant neoplasm of xiphoid process	Malignancy	CALIBER
Read	B303.00	Malignant neoplasm of ribs, sternum and clavicle	Malignancy	CALIBER
Read	B303z00	Malignant neoplasm of rib, sternum and clavicle NOS	Malignancy	CALIBER
Read	B304000	Malignant neoplasm of scapula	Malignancy	CALIBER
Read	B304100	Malignant neoplasm of acromion	Malignancy	CALIBER
Read	B304200	Malignant neoplasm of humerus	Malignancy	CALIBER
Read	B304300	Malignant neoplasm of radius	Malignancy	CALIBER
Read	B304400	Malignant neoplasm of ulna	Malignancy	CALIBER
Read	B304.00	Malignant neoplasm of scapula and long bones of upper arm	Malignancy	CALIBER
Read	B304z00	Malig neop of scapula and long bones of upper arm NOS	Malignancy	CALIBER
Read	B305000	Malignant neoplasm of carpal bone - scaphoid	Malignancy	CALIBER

Read	B305100	Malignant neoplasm of carpal bone - lunate	Malignancy	CALIBER
Read	B305A00	Malignant neoplasm of third metacarpal bone	Malignancy	CALIBER
Read	B305.00	Malignant neoplasm of hand bones	Malignancy	CALIBER
Read	B305.11	Malignant neoplasm of carpal bones	Malignancy	CALIBER
Read	B305.12	Malignant neoplasm of metacarpal bones	Malignancy	CALIBER
Read	B305C00	Malignant neoplasm of fifth metacarpal bone	Malignancy	CALIBER
Read	B305D00	Malignant neoplasm of phalanges of hand	Malignancy	CALIBER
Read	B305z00	Malignant neoplasm of hand bones NOS	Malignancy	CALIBER
Read	B306000	Malignant neoplasm of ilium	Malignancy	CALIBER
Read	B306100	Malignant neoplasm of ischium	Malignancy	CALIBER
Read	B306200	Malignant neoplasm of pubis	Malignancy	CALIBER
Read	B306300	Malignant neoplasm of sacral vertebra	Malignancy	CALIBER
Read	B306400	Malignant neoplasm of coccygeal vertebra	Malignancy	CALIBER
Read	B306500	Malignant sacral teratoma	Malignancy	CALIBER
Read	B306.00	Malignant neoplasm of pelvic bones, sacrum and coccyx	Malignancy	CALIBER
Read	B306z00	Malignant neoplasm of pelvis, sacrum or coccyx NOS	Malignancy	CALIBER
Read	B307000	Malignant neoplasm of femur	Malignancy	CALIBER
Read	B307100	Malignant neoplasm of fibula	Malignancy	CALIBER
Read	B307200	Malignant neoplasm of tibia	Malignancy	CALIBER
Read	B307.00	Malignant neoplasm of long bones of leg	Malignancy	CALIBER
Read	B307z00	Malignant neoplasm of long bones of leg NOS	Malignancy	CALIBER
Read	B308100	Malignant neoplasm of talus	Malignancy	CALIBER
Read	B308200	Malignant neoplasm of calcaneum	Malignancy	CALIBER
Read	B308300	Malignant neoplasm of medial cuneiform	Malignancy	CALIBER
Read	B308800	Malignant neoplasm of first metatarsal bone	Malignancy	CALIBER
Read	B308.00	Malignant neoplasm of short bones of leg	Malignancy	CALIBER
Read	B308B00	Malignant neoplasm of fourth metatarsal bone	Malignancy	CALIBER
Read	B308D00	Malignant neoplasm of phalanges of foot	Malignancy	CALIBER
Read	B308z00	Malignant neoplasm of short bones of leg NOS	Malignancy	CALIBER
Read	B30..00	Malignant neoplasm of bone and articular cartilage	Malignancy	CALIBER
Read	B30W.00	Malignant neoplasm/overlap lesion/bone+articulr cartilage	Malignancy	CALIBER
Read	B30X.00	Malignant neoplasm/bones+articular cartilage/limb,unspfd	Malignancy	CALIBER
Read	B30z000	Osteosarcoma	Malignancy	CALIBER

Read	B30z.00	Malignant neoplasm of bone and articular cartilage NOS	Malignancy	CALIBER
Read	BBV1.00	[M]Osteosarcoma NOS	Malignancy	CALIBER
Read	BBV1.11	[M]Osteoblastic sarcoma	Malignancy	CALIBER
Read	BBV1.12	[M]Osteochondrosarcoma	Malignancy	CALIBER
Read	BBV1.13	[M]Osteogenic sarcoma NOS	Malignancy	CALIBER
Read	BBV2.00	[M]Chondroblastic osteosarcoma	Malignancy	CALIBER
Read	BBV3.00	[M]Fibroblastic osteosarcoma	Malignancy	CALIBER
Read	BBV4.00	[M]Telangiectatic osteosarcoma	Malignancy	CALIBER
Read	BBV5.00	[M]Osteosarcoma in Paget's disease of bone	Malignancy	CALIBER
Read	BBV9.00	[M]Myxoid chondrosarcoma	Malignancy	CALIBER
Read	BBVA.00	[M] Small cell osteosarcoma	Malignancy	CALIBER
Read	BBV..11	[M]Juxtacortical osteogenic sarcoma	Malignancy	CALIBER
Read	BBV..12	[M]Parosteal osteosarcoma	Malignancy	CALIBER
Read	BBV..13	[M]Periosteal osteogenic sarcoma	Malignancy	CALIBER
Read	BBW4.00	[M]Chondrosarcoma NOS	Malignancy	CALIBER
Read	BBW4.11	[M]Fibrochondrosarcoma	Malignancy	CALIBER
Read	BBW6.00	[M]Juxtacortical chondrosarcoma	Malignancy	CALIBER
Read	BBW8.00	[M]Chondroblastoma, malignant	Malignancy	CALIBER
Read	BBW9.00	[M]Mesenchymal chondrosarcoma	Malignancy	CALIBER
Read	BBX1.00	[M]Giant cell tumour of bone, malignant	Malignancy	CALIBER
Read	BBX1.11	[M]Giant cell bone sarcoma	Malignancy	CALIBER
Read	BBX1.12	[M]Osteoclastoma, malignant	Malignancy	CALIBER
Read	BBY0.00	[M]Ewing's sarcoma	Malignancy	CALIBER
Read	BBY0.11	[M]Endothelial bone sarcoma	Malignancy	CALIBER
Read	BBY1.00	[M]Adamantinoma of long bones	Malignancy	CALIBER
Read	BBY1.11	[M]Tibial adamantinoma	Malignancy	CALIBER
Read	BBZ2.00	[M]Odontogenic tumour, malignant	Malignancy	CALIBER
Read	BBZ2.11	[M]Intraosseous carcinoma	Malignancy	CALIBER
Read	BBZC.00	[M]Ameloblastic odontosarcoma	Malignancy	CALIBER
Read	BBZG.00	[M]Ameloblastoma, malignant	Malignancy	CALIBER
Read	BBZG.11	[M]Adamantinoma, malignant	Malignancy	CALIBER
Read	BBZN.00	[M]Ameloblastic fibrosarcoma	Malignancy	CALIBER
Read	BBZN.11	[M]Odontogenic fibrosarcoma	Malignancy	CALIBER
Read	Byu3100	[X]Malignant neoplasm/bones+articular cartilage/limb,unspfd	Malignancy	CALIBER
Read	Byu3200	[X]Malignant neoplasm/overlap lesion/bone+articulr cartilage	Malignancy	CALIBER
Read	Byu3300	[X]Malignant neoplasm/bone+articular cartilage, unspecified	Malignancy	CALIBER
Read	Byu3.00	[X]Malignant neoplasm of bone and articular cartilage	Malignancy	CALIBER

ICD10	C40	Malignant neoplasm of bone and articular cartilage of limbs	Malignancy	CALIBER
ICD10	C41	Malignant neoplasm of bone and articular cartilage of other and unspecified sites	Malignancy	CALIBER
Read	B510000	Malignant neoplasm of basal ganglia	Malignancy	CALIBER
Read	B510100	Malignant neoplasm of cerebral cortex	Malignancy	CALIBER
Read	B510300	Malignant neoplasm of globus pallidus	Malignancy	CALIBER
Read	B510400	Malignant neoplasm of hypothalamus	Malignancy	CALIBER
Read	B510500	Malignant neoplasm of thalamus	Malignancy	CALIBER
Read	B510.00	Malignant neoplasm cerebrum (excluding lobes and ventricles)	Malignancy	CALIBER
Read	B510z00	Malignant neoplasm of cerebrum NOS	Malignancy	CALIBER
Read	B511.00	Malignant neoplasm of frontal lobe	Malignancy	CALIBER
Read	B512000	Malignant neoplasm of hippocampus	Malignancy	CALIBER
Read	B512.00	Malignant neoplasm of temporal lobe	Malignancy	CALIBER
Read	B512z00	Malignant neoplasm of temporal lobe NOS	Malignancy	CALIBER
Read	B513.00	Malignant neoplasm of parietal lobe	Malignancy	CALIBER
Read	B514.00	Malignant neoplasm of occipital lobe	Malignancy	CALIBER
Read	B515000	Malignant neoplasm of choroid plexus	Malignancy	CALIBER
Read	B515.00	Malignant neoplasm of cerebral ventricles	Malignancy	CALIBER
Read	B516.00	Malignant neoplasm of cerebellum	Malignancy	CALIBER
Read	B517000	Malignant neoplasm of cerebral peduncle	Malignancy	CALIBER
Read	B517100	Malignant neoplasm of medulla oblongata	Malignancy	CALIBER
Read	B517200	Malignant neoplasm of midbrain	Malignancy	CALIBER
Read	B517300	Malignant neoplasm of pons	Malignancy	CALIBER
Read	B517.00	Malignant neoplasm of brain stem	Malignancy	CALIBER
Read	B517z00	Malignant neoplasm of brain stem NOS	Malignancy	CALIBER
Read	B51..00	Malignant neoplasm of brain	Malignancy	CALIBER
Read	B51..11	Cerebral tumour - malignant	Malignancy	CALIBER
Read	B51y000	Malignant neoplasm of corpus callosum	Malignancy	CALIBER
Read	B51y200	Malignant neoplasm, overlapping lesion of brain	Malignancy	CALIBER
Read	B51y.00	Malignant neoplasm of other parts of brain	Malignancy	CALIBER
Read	B51yz00	Malignant neoplasm of other part of brain NOS	Malignancy	CALIBER
Read	B51z.00	Malignant neoplasm of brain NOS	Malignancy	CALIBER
Read	B520000	Malignant neoplasm of olfactory bulb	Malignancy	CALIBER
Read	B520100	Malignant neoplasm of optic nerve	Malignancy	CALIBER
Read	B520200	Malignant neoplasm of acoustic nerve	Malignancy	CALIBER
Read	B520.00	Malignant neoplasm of cranial nerves	Malignancy	CALIBER
Read	B520z00	Malignant neoplasm of cranial nerves NOS	Malignancy	CALIBER
Read	B521200	Malignant neoplasm of cerebral pia mater	Malignancy	CALIBER
Read	B521.00	Malignant neoplasm of cerebral meninges	Malignancy	CALIBER

Read	B521z00	Malignant neoplasm of cerebral meninges NOS	Malignancy	CALIBER
Read	B522.00	Malignant neoplasm of spinal cord	Malignancy	CALIBER
Read	B523.00	Malignant neoplasm of spinal meninges	Malignancy	CALIBER
Read	B523z00	Malignant neoplasm of spinal meninges NOS	Malignancy	CALIBER
Read	B525.00	Malignant neoplasm of cauda equina	Malignancy	CALIBER
Read	B52W.00	Malig neopl, overlap lesion brain & other part of CNS	Malignancy	CALIBER
Read	B52X.00	Malignant neoplasm of meninges, unspecified	Malignancy	CALIBER
Read	B542000	Malignant neoplasm of pituitary gland	Malignancy	CALIBER
Read	B542100	Malignant neoplasm of craniopharyngeal duct	Malignancy	CALIBER
Read	B542.00	Malignant neoplasm pituitary gland and craniopharyngeal duct	Malignancy	CALIBER
Read	B542z00	Malig neop pituitary gland or craniopharyngeal duct NOS	Malignancy	CALIBER
Read	B543.00	Malignant neoplasm of pineal gland	Malignancy	CALIBER
Read	B544.00	Malignant neoplasm of carotid body	Malignancy	CALIBER
Read	B545000	Malignant neoplasm of glomus jugulare	Malignancy	CALIBER
Read	B545100	Malignant neoplasm of aortic body	Malignancy	CALIBER
Read	B545200	Malignant neoplasm of coccygeal body	Malignancy	CALIBER
Read	B545.00	Malignant neoplasm of aortic body and other paraganglia	Malignancy	CALIBER
Read	B545z00	Malignant neoplasm of aortic body or paraganglia NOS	Malignancy	CALIBER
Read	BBbB.00	[M]Astrocytoma NOS	Malignancy	CALIBER
Read	BBbB.11	[M]Astrocytic glioma	Malignancy	CALIBER
Read	BBbC.00	[M]Astrocytoma, anaplastic type	Malignancy	CALIBER
Read	BBbE.00	[M]Gemistocytic astrocytoma	Malignancy	CALIBER
Read	BBbF.00	[M]Fibrillary astrocytoma	Malignancy	CALIBER
Read	BBbG.00	[M]Pilocytic astrocytoma	Malignancy	CALIBER
Read	BBbG.11	[M]Juvenile astrocytoma	Malignancy	CALIBER
Read	BBbG.12	[M]Piloid astrocytoma	Malignancy	CALIBER
Read	BBbH.00	[M]Spongioblastoma NOS	Malignancy	CALIBER
Read	BBbK.00	[M]Astroblastoma	Malignancy	CALIBER
Read	BBbL.00	[M]Glioblastoma NOS	Malignancy	CALIBER
Read	BBbL.11	[M]Glioblastoma multiforme	Malignancy	CALIBER
Read	BBbM.00	[M]Giant cell glioblastoma	Malignancy	CALIBER
Read	BBbQ.00	[M]Oligodendroglioma NOS	Malignancy	CALIBER
Read	BBbR.00	[M]Oligodendroglioma, anaplastic type	Malignancy	CALIBER
Read	BBbS.00	[M]Oligodendroblastoma	Malignancy	CALIBER
Read	BBbT.00	[M]Medulloblastoma NOS	Malignancy	CALIBER

Read	BBbU.00	[M]Desmoplastic medulloblastoma	Malignancy	CALIBER
Read	BBbV.00	[M]Medullomyoblastoma	Malignancy	CALIBER
Read	BBbW.00	[M]Cerebellar sarcoma NOS	Malignancy	CALIBER
Read	BBbz.00	[M]Glioma NOS	Malignancy	CALIBER
Read	BBbZ.00	[M]Pleomorphic xanthoastrocytoma	Malignancy	CALIBER
Read	BBc2.00	[M]Medulloepithelioma NOS	Malignancy	CALIBER
Read	BBc6.11	[M]Glioneuroma	Malignancy	CALIBER
Read	BBc7.11	[M]Neuroastrocytoma	Malignancy	CALIBER
Read	BBcA.00	[M]Olfactory neurogenic tumour	Malignancy	CALIBER
Read	BBd2.00	[M]Meningioma, malignant	Malignancy	CALIBER
Read	BBd2.12	[M]Meningothelial sarcoma	Malignancy	CALIBER
Read	BBdA.00	[M]Papillary meningioma	Malignancy	CALIBER
Read	ByuA000	[X]Malignant neoplasm/other and unspecified cranial nerves	Malignancy	CALIBER
Read	ByuA100	[X]Malignant neoplasm/central nervous system, unspecified	Malignancy	CALIBER
Read	ByuA200	[X]Malignant neoplasm of meninges, unspecified	Malignancy	CALIBER
Read	ByuA300	[X]Malig neopl, overlap lesion brain & other part of CNS	Malignancy	CALIBER
	C70	Malignant neoplasm of meninges	Malignancy	CALIBER
	C71	Malignant neoplasm of brain	Malignancy	CALIBER
	C72	Malignant neoplasm of spinal cord, cranial nerves and other parts of central nervous system	Malignancy	CALIBER
	C75.1	Malignant neoplasm: Pituitary gland	Malignancy	CALIBER
	C75.2	Malignant neoplasm: Craniopharyngeal duct	Malignancy	CALIBER
	C75.3	Malignant neoplasm: Pineal gland	Malignancy	CALIBER
	C75.4	Malignant neoplasm: Carotid body	Malignancy	CALIBER
	C75.5	Malignant neoplasm: Aortic body and other paraganglia	Malignancy	CALIBER
Read	B340000	Malignant neoplasm of nipple of female breast	Malignancy	CALIBER
Read	B340100	Malignant neoplasm of areola of female breast	Malignancy	CALIBER
Read	B340.00	Malignant neoplasm of nipple and areola of female breast	Malignancy	CALIBER
Read	B340z00	Malignant neoplasm of nipple or areola of female breast NOS	Malignancy	CALIBER
Read	B341.00	Malignant neoplasm of central part of female breast	Malignancy	CALIBER
Read	B342.00	Malignant neoplasm of upper-inner quadrant of female breast	Malignancy	CALIBER
Read	B343.00	Malignant neoplasm of lower-inner quadrant of female breast	Malignancy	CALIBER

Read	B344.00	Malignant neoplasm of upper-outer quadrant of female breast	Malignancy	CALIBER
Read	B345.00	Malignant neoplasm of lower-outer quadrant of female breast	Malignancy	CALIBER
Read	B346.00	Malignant neoplasm of axillary tail of female breast	Malignancy	CALIBER
Read	B347.00	Malignant neoplasm, overlapping lesion of breast	Malignancy	CALIBER
Read	B34..00	Malignant neoplasm of female breast	Malignancy	CALIBER
Read	B34..11	Ca female breast	Malignancy	CALIBER
Read	B34y000	Malignant neoplasm of ectopic site of female breast	Malignancy	CALIBER
Read	B34y.00	Malignant neoplasm of other site of female breast	Malignancy	CALIBER
Read	B34yz00	Malignant neoplasm of other site of female breast NOS	Malignancy	CALIBER
Read	B34z.00	Malignant neoplasm of female breast NOS	Malignancy	CALIBER
Read	B350000	Malignant neoplasm of nipple of male breast	Malignancy	CALIBER
Read	B350100	Malignant neoplasm of areola of male breast	Malignancy	CALIBER
Read	B350.00	Malignant neoplasm of nipple and areola of male breast	Malignancy	CALIBER
Read	B35..00	Malignant neoplasm of male breast	Malignancy	CALIBER
Read	B35z000	Malignant neoplasm of ectopic site of male breast	Malignancy	CALIBER
Read	B35z.00	Malignant neoplasm of other site of male breast	Malignancy	CALIBER
Read	B35zz00	Malignant neoplasm of male breast NOS	Malignancy	CALIBER
Read	B36..00	Local recurrence of malignant tumour of breast	Malignancy	CALIBER
Read	B830000	Lobular carcinoma in situ of breast	Malignancy	CALIBER
Read	B830100	Intraductal carcinoma in situ of breast	Malignancy	CALIBER
Read	B830.00	Carcinoma in situ of breast	Malignancy	CALIBER
Read	BB91.00	[M]Infiltrating duct carcinoma	Malignancy	CALIBER
Read	BB91.11	[M]Duct carcinoma NOS	Malignancy	CALIBER
Read	BB91000	[M]Intraductal papillary adenocarcinoma with invasion	Malignancy	CALIBER
Read	BB96.00	[M]Noninfiltrating intraductal papillary adenocarcinoma	Malignancy	CALIBER
Read	BB91100	[M]Infiltrating duct and lobular carcinoma	Malignancy	CALIBER
Read	BB92.00	[M]Comedocarcinoma, noninfiltrating	Malignancy	CALIBER
Read	BB93.00	[M]Comedocarcinoma NOS	Malignancy	CALIBER
Read	BB94.00	[M]Juvenile breast carcinoma	Malignancy	CALIBER
Read	BB94.11	[M]Secretory breast carcinoma	Malignancy	CALIBER
Read	BB9J.00	[M]Paget's disease, mammary	Malignancy	CALIBER

Read	BB9J.11	[M]Paget's disease, breast	Malignancy	CALIBER
Read	BB9K000	[M]Paget's disease and intraductal carcinoma of breast	Malignancy	CALIBER
Read	BB9K.00	[M]Paget's disease and infiltrating breast duct carcinoma	Malignancy	CALIBER
Read	BB9M.00	[M]Intracystic carcinoma NOS	Malignancy	CALIBER
Read	Byu6.00	[X]Malignant neoplasm of breast	Malignancy	CALIBER
Read	ByuFG00	[X]Other carcinoma in situ of breast	Malignancy	CALIBER
ICD10	C50	Malignant neoplasm of breast	Malignancy	CALIBER
ICD10	D05	Carcinoma in situ of breast	Malignancy	CALIBER
Read	B410000	Malignant neoplasm of endocervical canal	Malignancy	CALIBER
Read	B410100	Malignant neoplasm of endocervical gland	Malignancy	CALIBER
Read	B410.00	Malignant neoplasm of endocervix	Malignancy	CALIBER
Read	B410z00	Malignant neoplasm of endocervix NOS	Malignancy	CALIBER
Read	B411.00	Malignant neoplasm of exocervix	Malignancy	CALIBER
Read	B412.00	Malignant neoplasm, overlapping lesion of cervix uteri	Malignancy	CALIBER
Read	B41..00	Malignant neoplasm of cervix uteri	Malignancy	CALIBER
Read	B41..11	Cervical carcinoma (uterus)	Malignancy	CALIBER
Read	B41y000	Malignant neoplasm of cervical stump	Malignancy	CALIBER
Read	B41y100	Malignant neoplasm of squamocolumnar junction of cervix	Malignancy	CALIBER
Read	B41y.00	Malignant neoplasm of other site of cervix	Malignancy	CALIBER
Read	B41yz00	Malignant neoplasm of other site of cervix NOS	Malignancy	CALIBER
Read	B41z.00	Malignant neoplasm of cervix uteri NOS	Malignancy	CALIBER
ICD10	C53	Malignant neoplasm of cervix uteri	Malignancy	CALIBER
Read	B4A0000	Hypernephroma	Malignancy	CALIBER
Read	B4A0.00	Malignant neoplasm of kidney parenchyma	Malignancy	CALIBER
Read	B4A1000	Malignant neoplasm of renal calyces	Malignancy	CALIBER
Read	B4A1100	Malignant neoplasm of ureteropelvic junction	Malignancy	CALIBER
Read	B4A1.00	Malignant neoplasm of renal pelvis	Malignancy	CALIBER
Read	B4A1z00	Malignant neoplasm of renal pelvis NOS	Malignancy	CALIBER
Read	B4A2.00	Malignant neoplasm of ureter	Malignancy	CALIBER
Read	B4A..11	Renal malignant neoplasm	Malignancy	CALIBER
Read	BB5a000	[M]Renal cell carcinoma	Malignancy	CALIBER
Read	BB5a011	[M]Grawitz tumour	Malignancy	CALIBER
Read	BB5a012	[M]Hypernephroma	Malignancy	CALIBER
ICD10	C64	Malignant neoplasm of kidney, except renal pelvis	Malignancy	CALIBER
ICD10	C65	Malignant neoplasm of renal pelvis	Malignancy	CALIBER
ICD10	C66	Malignant neoplasm of ureter	Malignancy	CALIBER

Read	B150000	Primary carcinoma of liver	Malignancy	CALIBER
Read	B150100	Hepatoblastoma of liver	Malignancy	CALIBER
Read	B150200	Primary angiosarcoma of liver	Malignancy	CALIBER
Read	B150300	Hepatocellular carcinoma	Malignancy	CALIBER
Read	B150.00	Primary malignant neoplasm of liver	Malignancy	CALIBER
Read	B150z00	Primary malignant neoplasm of liver NOS	Malignancy	CALIBER
Read	B152.00	Malignant neoplasm of liver unspecified	Malignancy	CALIBER
Read	BB5D500	[M]Hepatocellular carcinoma NOS	Malignancy	CALIBER
Read	BB5D511	[M]Hepatoma NOS	Malignancy	CALIBER
Read	BB5D512	[M]Hepatoma, malignant	Malignancy	CALIBER
Read	BB5D513	[M]Liver cell carcinoma	Malignancy	CALIBER
Read	BB5D700	[M]Combined hepatocellular carcinoma and cholangiocarcinoma	Malignancy	CALIBER
Read	BB5D711	[M]Hepatocholangiocarcinoma	Malignancy	CALIBER
Read	BB5D800	[M]Hepatocellular carcinoma, fibrolamellar	Malignancy	CALIBER
Read	Byu1100	[X]Other specified carcinomas of liver	Malignancy	CALIBER
ICD10	C22.0	Malignant neoplasm: Liver cell carcinoma	Malignancy	CALIBER
ICD10	C22.2	Malignant neoplasm: Hepatoblastoma	Malignancy	CALIBER
ICD10	C22.3	Malignant neoplasm: Angiosarcoma of liver	Malignancy	CALIBER
ICD10	C22.4	Malignant neoplasm: Other sarcomas of liver	Malignancy	CALIBER
ICD10	C22.7	Malignant neoplasm: Other specified carcinomas of liver	Malignancy	CALIBER
ICD10	C22.9	Malignant neoplasm: Liver, unspecified	Malignancy	CALIBER
Read	B220100	Malignant neoplasm of mucosa of trachea	Malignancy	CALIBER
Read	B220.00	Malignant neoplasm of trachea	Malignancy	CALIBER
Read	B220z00	Malignant neoplasm of trachea NOS	Malignancy	CALIBER
Read	B221000	Malignant neoplasm of carina of bronchus	Malignancy	CALIBER
Read	B221100	Malignant neoplasm of hilus of lung	Malignancy	CALIBER
Read	B221.00	Malignant neoplasm of main bronchus	Malignancy	CALIBER
Read	B221z00	Malignant neoplasm of main bronchus NOS	Malignancy	CALIBER
Read	B222000	Malignant neoplasm of upper lobe bronchus	Malignancy	CALIBER
Read	B222100	Malignant neoplasm of upper lobe of lung	Malignancy	CALIBER
Read	B222.00	Malignant neoplasm of upper lobe, bronchus or lung	Malignancy	CALIBER
Read	B222.11	Pancoast's syndrome	Malignancy	CALIBER
Read	B222z00	Malignant neoplasm of upper lobe, bronchus or lung NOS	Malignancy	CALIBER
Read	B223000	Malignant neoplasm of middle lobe bronchus	Malignancy	CALIBER
Read	B223100	Malignant neoplasm of middle lobe of lung	Malignancy	CALIBER
Read	B223.00	Malignant neoplasm of middle lobe, bronchus or lung	Malignancy	CALIBER

Read	B223z00	Malignant neoplasm of middle lobe, bronchus or lung NOS	Malignancy	CALIBER
Read	B224000	Malignant neoplasm of lower lobe bronchus	Malignancy	CALIBER
Read	B224100	Malignant neoplasm of lower lobe of lung	Malignancy	CALIBER
Read	B224.00	Malignant neoplasm of lower lobe, bronchus or lung	Malignancy	CALIBER
Read	B224z00	Malignant neoplasm of lower lobe, bronchus or lung NOS	Malignancy	CALIBER
Read	B225.00	Malignant neoplasm of overlapping lesion of bronchus & lung	Malignancy	CALIBER
Read	B22..00	Malignant neoplasm of trachea, bronchus and lung	Malignancy	CALIBER
Read	B22y.00	Malignant neoplasm of other sites of bronchus or lung	Malignancy	CALIBER
Read	B22z.00	Malignant neoplasm of bronchus or lung NOS	Malignancy	CALIBER
Read	B22z.11	Lung cancer	Malignancy	CALIBER
Read	BB5S200	[M]Bronchiolo-alveolar adenocarcinoma	Malignancy	CALIBER
Read	BB5S211	[M]Alveolar cell carcinoma	Malignancy	CALIBER
Read	BB5S212	[M]Bronchiolar carcinoma	Malignancy	CALIBER
Read	BB5S400	[M]Alveolar adenocarcinoma	Malignancy	CALIBER
Read	Byu2000	[X]Malignant neoplasm of bronchus or lung, unspecified	Malignancy	CALIBER
ICD10	C33	Malignant neoplasm of trachea	Malignancy	CALIBER
ICD10	C34	Malignant neoplasm of bronchus and lung	Malignancy	CALIBER
Read	4M3..00	Breslow depth staging for melanoma	Malignancy	CALIBER
Read	4M70.00	Clark melanoma level 1	Malignancy	CALIBER
Read	4M71.00	Clark melanoma level 2	Malignancy	CALIBER
Read	4M72.00	Clark melanoma level 3	Malignancy	CALIBER
Read	4M73.00	Clark melanoma level 4	Malignancy	CALIBER
Read	4M74.00	Clark melanoma level 5	Malignancy	CALIBER
Read	7G03J00	Excision of melanoma	Malignancy	CALIBER
Read	B320.00	Malignant melanoma of lip	Malignancy	CALIBER
Read	B321.00	Malignant melanoma of eyelid including canthus	Malignancy	CALIBER
Read	B322000	Malignant melanoma of auricle (ear)	Malignancy	CALIBER
Read	B322100	Malignant melanoma of external auditory meatus	Malignancy	CALIBER
Read	B322.00	Malignant melanoma of ear and external auricular canal	Malignancy	CALIBER
Read	B322z00	Malignant melanoma of ear and external auricular canal NOS	Malignancy	CALIBER
Read	B323000	Malignant melanoma of external surface of cheek	Malignancy	CALIBER
Read	B323100	Malignant melanoma of chin	Malignancy	CALIBER

Read	B323200	Malignant melanoma of eyebrow	Malignancy	CALIBER
Read	B323300	Malignant melanoma of forehead	Malignancy	CALIBER
Read	B323400	Malignant melanoma of external surface of nose	Malignancy	CALIBER
Read	B323500	Malignant melanoma of temple	Malignancy	CALIBER
Read	B323.00	Malignant melanoma of other and unspecified parts of face	Malignancy	CALIBER
Read	B323z00	Malignant melanoma of face NOS	Malignancy	CALIBER
Read	B324000	Malignant melanoma of scalp	Malignancy	CALIBER
Read	B324100	Malignant melanoma of neck	Malignancy	CALIBER
Read	B324.00	Malignant melanoma of scalp and neck	Malignancy	CALIBER
Read	B324z00	Malignant melanoma of scalp and neck NOS	Malignancy	CALIBER
Read	B325000	Malignant melanoma of axilla	Malignancy	CALIBER
Read	B325100	Malignant melanoma of breast	Malignancy	CALIBER
Read	B325200	Malignant melanoma of buttock	Malignancy	CALIBER
Read	B325300	Malignant melanoma of groin	Malignancy	CALIBER
Read	B325400	Malignant melanoma of perianal skin	Malignancy	CALIBER
Read	B325500	Malignant melanoma of perineum	Malignancy	CALIBER
Read	B325600	Malignant melanoma of umbilicus	Malignancy	CALIBER
Read	B325700	Malignant melanoma of back	Malignancy	CALIBER
Read	B325800	Malignant melanoma of chest wall	Malignancy	CALIBER
Read	B325.00	Malignant melanoma of trunk (excluding scrotum)	Malignancy	CALIBER
Read	B325z00	Malignant melanoma of trunk, excluding scrotum, NOS	Malignancy	CALIBER
Read	B326000	Malignant melanoma of shoulder	Malignancy	CALIBER
Read	B326100	Malignant melanoma of upper arm	Malignancy	CALIBER
Read	B326200	Malignant melanoma of fore-arm	Malignancy	CALIBER
Read	B326300	Malignant melanoma of hand	Malignancy	CALIBER
Read	B326400	Malignant melanoma of finger	Malignancy	CALIBER
Read	B326500	Malignant melanoma of thumb	Malignancy	CALIBER
Read	B326.00	Malignant melanoma of upper limb and shoulder	Malignancy	CALIBER
Read	B326z00	Malignant melanoma of upper limb or shoulder NOS	Malignancy	CALIBER
Read	B327000	Malignant melanoma of hip	Malignancy	CALIBER
Read	B327100	Malignant melanoma of thigh	Malignancy	CALIBER
Read	B327200	Malignant melanoma of knee	Malignancy	CALIBER
Read	B327300	Malignant melanoma of popliteal fossa area	Malignancy	CALIBER
Read	B327400	Malignant melanoma of lower leg	Malignancy	CALIBER
Read	B327500	Malignant melanoma of ankle	Malignancy	CALIBER
Read	B327600	Malignant melanoma of heel	Malignancy	CALIBER
Read	B327700	Malignant melanoma of foot	Malignancy	CALIBER

Read	B327800	Malignant melanoma of toe	Malignancy	CALIBER
Read	B327900	Malignant melanoma of great toe	Malignancy	CALIBER
Read	B327.00	Malignant melanoma of lower limb and hip	Malignancy	CALIBER
Read	B327z00	Malignant melanoma of lower limb or hip NOS	Malignancy	CALIBER
Read	B32..00	Malignant melanoma of skin	Malignancy	CALIBER
Read	B32y000	Overlapping malignant melanoma of skin	Malignancy	CALIBER
Read	B32y.00	Malignant melanoma of other specified skin site	Malignancy	CALIBER
Read	B32z.00	Malignant melanoma of skin NOS	Malignancy	CALIBER
Read	BBE1000	[M]Malignant melanoma, regressing	Malignancy	CALIBER
Read	BBE1100	[M]Desmoplastic melanoma, malignant	Malignancy	CALIBER
Read	BBE1.00	[M]Malignant melanoma NOS	Malignancy	CALIBER
Read	BBE1.11	[M]Melanocarcinoma	Malignancy	CALIBER
Read	BBE1.12	[M]Melanoma NOS	Malignancy	CALIBER
Read	BBE1.13	[M]Melanosarcoma NOS	Malignancy	CALIBER
Read	BBE2.00	[M]Nodular melanoma	Malignancy	CALIBER
Read	BBE4.00	[M]Balloon cell melanoma	Malignancy	CALIBER
Read	BBEA.00	[M]Amelanotic melanoma	Malignancy	CALIBER
Read	BBEC.00	[M]Malignant melanoma in junctional naevus	Malignancy	CALIBER
Read	BBEF.00	[M]Hutchinson's melanotic freckle	Malignancy	CALIBER
Read	BBEF.11	[M]Lentigo maligna	Malignancy	CALIBER
Read	BBEG000	[M]Acral lentiginous melanoma, malignant	Malignancy	CALIBER
Read	BBEG.00	[M]Malignant melanoma in Hutchinson's melanotic freckle	Malignancy	CALIBER
Read	BBEG.11	[M]Lentigo maligna melanoma	Malignancy	CALIBER
Read	BBEH.00	[M]Superficial spreading melanoma	Malignancy	CALIBER
Read	BBEM.00	[M]Malignant melanoma in giant pigmented naevus	Malignancy	CALIBER
Read	BBEN.11	[M]Juvenila melanoma	Malignancy	CALIBER
Read	BBEP.00	[M]Epithelioid cell melanoma	Malignancy	CALIBER
Read	BBEQ.00	[M]Spindle cell melanoma NOS	Malignancy	CALIBER
Read	BBES.00	[M]Spindle cell melanoma, type B	Malignancy	CALIBER
Read	BBET.00	[M]Mixed epithelioid and spindle melanoma	Malignancy	CALIBER
Read	Byu4000	[X]Malignant melanoma of other+unspecified parts of face	Malignancy	CALIBER
Read	Byu4100	[X]Malignant melanoma of skin, unspecified	Malignancy	CALIBER
ICD10	C43	Malignant melanoma of skin	Malignancy	CALIBER
Read	BBP1.00	[M]Mesothelioma, malignant	Malignancy	CALIBER
Read	BBP3.11	[M]Sarcomatoid mesothelioma	Malignancy	CALIBER
Read	BBP5.00	[M]Epithelioid mesothelioma, malignant	Malignancy	CALIBER

Read	BBP7.00	[M]Mesothelioma, biphasic type, malignant	Malignancy	CALIBER
ICD10	C45	Mesothelioma	Malignancy	CALIBER
Read	B592.00	Malignant neoplasms of independent (primary) multiple sites	Malignancy	CALIBER
Read	ByuE000	[X]Malignant neoplasms/independent(primary)multiple sites	Malignancy	CALIBER
Read	ByuE.00	[X]Malignant neoplasms/independent (primary) multiple sites	Malignancy	CALIBER
ICD10	C97	Malignant neoplasms of independent (primary) multiple sites	Malignancy	CALIBER
Read	B100.00	Malignant neoplasm of cervical oesophagus	Malignancy	CALIBER
Read	B101.00	Malignant neoplasm of thoracic oesophagus	Malignancy	CALIBER
Read	B102.00	Malignant neoplasm of abdominal oesophagus	Malignancy	CALIBER
Read	B103.00	Malignant neoplasm of upper third of oesophagus	Malignancy	CALIBER
Read	B104.00	Malignant neoplasm of middle third of oesophagus	Malignancy	CALIBER
Read	B105.00	Malignant neoplasm of lower third of oesophagus	Malignancy	CALIBER
Read	B106.00	Malignant neoplasm, overlapping lesion of oesophagus	Malignancy	CALIBER
Read	B107.00	Siewert type I adenocarcinoma	Malignancy	CALIBER
Read	B10..00	Malignant neoplasm of oesophagus	Malignancy	CALIBER
Read	B10y.00	Malignant neoplasm of other specified part of oesophagus	Malignancy	CALIBER
Read	B10z.00	Malignant neoplasm of oesophagus NOS	Malignancy	CALIBER
Read	B10z.11	Oesophageal cancer	Malignancy	CALIBER
ICD10	C15	Malignant neoplasm of oesophagus	Malignancy	CALIBER
Read	B000000	Malignant neoplasm of upper lip, external	Malignancy	CALIBER
Read	B000100	Malignant neoplasm of upper lip, lipstick area	Malignancy	CALIBER
Read	B000.00	Malignant neoplasm of upper lip, vermilion border	Malignancy	CALIBER
Read	B000z00	Malignant neoplasm of upper lip, vermilion border NOS	Malignancy	CALIBER
Read	B001000	Malignant neoplasm of lower lip, external	Malignancy	CALIBER
Read	B001100	Malignant neoplasm of lower lip, lipstick area	Malignancy	CALIBER
Read	B001.00	Malignant neoplasm of lower lip, vermilion border	Malignancy	CALIBER
Read	B001z00	Malignant neoplasm of lower lip, vermilion border NOS	Malignancy	CALIBER
Read	B002100	Malignant neoplasm of upper lip, frenulum	Malignancy	CALIBER
Read	B002200	Malignant neoplasm of upper lip, mucosa	Malignancy	CALIBER

Read	B002300	Malignant neoplasm of upper lip, oral aspect	Malignancy	CALIBER
Read	B002.00	Malignant neoplasm of upper lip, inner aspect	Malignancy	CALIBER
Read	B002z00	Malignant neoplasm of upper lip, inner aspect NOS	Malignancy	CALIBER
Read	B003000	Malignant neoplasm of lower lip, buccal aspect	Malignancy	CALIBER
Read	B003100	Malignant neoplasm of lower lip, frenulum	Malignancy	CALIBER
Read	B003200	Malignant neoplasm of lower lip, mucosa	Malignancy	CALIBER
Read	B003300	Malignant neoplasm of lower lip, oral aspect	Malignancy	CALIBER
Read	B003.00	Malignant neoplasm of lower lip, inner aspect	Malignancy	CALIBER
Read	B003z00	Malignant neoplasm of lower lip, inner aspect NOS	Malignancy	CALIBER
Read	B004000	Malignant neoplasm of lip unspecified, buccal aspect	Malignancy	CALIBER
Read	B004200	Malignant neoplasm of lip unspecified, mucosa	Malignancy	CALIBER
Read	B004300	Malignant neoplasm of lip, oral aspect	Malignancy	CALIBER
Read	B004.00	Malignant neoplasm of lip unspecified, inner aspect	Malignancy	CALIBER
Read	B005.00	Malignant neoplasm of commissure of lip	Malignancy	CALIBER
Read	B006.00	Malignant neoplasm of overlapping lesion of lip	Malignancy	CALIBER
Read	B007.00	Malignant neoplasm of lip, unspecified	Malignancy	CALIBER
Read	B00..00	Malignant neoplasm of lip	Malignancy	CALIBER
Read	B00..11	Carcinoma of lip	Malignancy	CALIBER
Read	B00z000	Malignant neoplasm of lip, unspecified, external	Malignancy	CALIBER
Read	B00z100	Malignant neoplasm of lip, unspecified, lipstick area	Malignancy	CALIBER
Read	B00zz00	Malignant neoplasm of lip, vermilion border NOS	Malignancy	CALIBER
Read	B010000	Malignant neoplasm of base of tongue dorsal surface	Malignancy	CALIBER
Read	B010.00	Malignant neoplasm of base of tongue	Malignancy	CALIBER
Read	B010.11	Malignant neoplasm of posterior third of tongue	Malignancy	CALIBER
Read	B010z00	Malignant neoplasm of fixed part of tongue NOS	Malignancy	CALIBER
Read	B011100	Malignant neoplasm of midline of tongue	Malignancy	CALIBER
Read	B011.00	Malignant neoplasm of dorsal surface of tongue	Malignancy	CALIBER
Read	B011z00	Malignant neoplasm of dorsum of tongue NOS	Malignancy	CALIBER

Read	B012.00	Malignant neoplasm of tongue, tip and lateral border	Malignancy	CALIBER
Read	B013000	Malignant neoplasm of anterior 2/3 of tongue ventral surface	Malignancy	CALIBER
Read	B013100	Malignant neoplasm of frenulum linguae	Malignancy	CALIBER
Read	B013.00	Malignant neoplasm of ventral surface of tongue	Malignancy	CALIBER
Read	B013z00	Malignant neoplasm of ventral tongue surface NOS	Malignancy	CALIBER
Read	B014.00	Malignant neoplasm of anterior 2/3 of tongue unspecified	Malignancy	CALIBER
Read	B015.00	Malignant neoplasm of tongue, junctional zone	Malignancy	CALIBER
Read	B016.00	Malignant neoplasm of lingual tonsil	Malignancy	CALIBER
Read	B017.00	Malignant overlapping lesion of tongue	Malignancy	CALIBER
Read	B01..00	Malignant neoplasm of tongue	Malignancy	CALIBER
Read	B01y.00	Malignant neoplasm of other sites of tongue	Malignancy	CALIBER
Read	B01z.00	Malignant neoplasm of tongue NOS	Malignancy	CALIBER
Read	B020.00	Malignant neoplasm of parotid gland	Malignancy	CALIBER
Read	B021.00	Malignant neoplasm of submandibular gland	Malignancy	CALIBER
Read	B022.00	Malignant neoplasm of sublingual gland	Malignancy	CALIBER
Read	B02..00	Malignant neoplasm of major salivary glands	Malignancy	CALIBER
Read	B02y.00	Malignant neoplasm of other major salivary glands	Malignancy	CALIBER
Read	B02z.00	Malignant neoplasm of major salivary gland NOS	Malignancy	CALIBER
Read	B030.00	Malignant neoplasm of upper gum	Malignancy	CALIBER
Read	B031.00	Malignant neoplasm of lower gum	Malignancy	CALIBER
Read	B03..00	Malignant neoplasm of gum	Malignancy	CALIBER
Read	B03y.00	Malignant neoplasm of other sites of gum	Malignancy	CALIBER
Read	B03z.00	Malignant neoplasm of gum NOS	Malignancy	CALIBER
Read	B040.00	Malignant neoplasm of anterior portion of floor of mouth	Malignancy	CALIBER
Read	B041.00	Malignant neoplasm of lateral portion of floor of mouth	Malignancy	CALIBER
Read	B042.00	Malignant neoplasm, overlapping lesion of floor of mouth	Malignancy	CALIBER
Read	B04..00	Malignant neoplasm of floor of mouth	Malignancy	CALIBER
Read	B04y.00	Malignant neoplasm of other sites of floor of mouth	Malignancy	CALIBER
Read	B04z.00	Malignant neoplasm of floor of mouth NOS	Malignancy	CALIBER
Read	B050.00	Malignant neoplasm of cheek mucosa	Malignancy	CALIBER
Read	B050.11	Malignant neoplasm of buccal mucosa	Malignancy	CALIBER

Read	B051000	Malignant neoplasm of upper buccal sulcus	Malignancy	CALIBER
Read	B051100	Malignant neoplasm of lower buccal sulcus	Malignancy	CALIBER
Read	B051.00	Malignant neoplasm of vestibule of mouth	Malignancy	CALIBER
Read	B052.00	Malignant neoplasm of hard palate	Malignancy	CALIBER
Read	B053.00	Malignant neoplasm of soft palate	Malignancy	CALIBER
Read	B054.00	Malignant neoplasm of uvula	Malignancy	CALIBER
Read	B055000	Malignant neoplasm of junction of hard and soft palate	Malignancy	CALIBER
Read	B055100	Malignant neoplasm of roof of mouth	Malignancy	CALIBER
Read	B055.00	Malignant neoplasm of palate unspecified	Malignancy	CALIBER
Read	B055z00	Malignant neoplasm of palate NOS	Malignancy	CALIBER
Read	B056.00	Malignant neoplasm of retromolar area	Malignancy	CALIBER
Read	B05..00	Malignant neoplasm of other and unspecified parts of mouth	Malignancy	CALIBER
Read	B05y.00	Malignant neoplasm of other specified mouth parts	Malignancy	CALIBER
Read	B05z.00	Malignant neoplasm of mouth NOS	Malignancy	CALIBER
Read	B060000	Malignant neoplasm of faucial tonsil	Malignancy	CALIBER
Read	B060100	Malignant neoplasm of palatine tonsil	Malignancy	CALIBER
Read	B060200	Malignant neoplasm of overlapping lesion of tonsil	Malignancy	CALIBER
Read	B060.00	Malignant neoplasm of tonsil	Malignancy	CALIBER
Read	B060z00	Malignant neoplasm tonsil NOS	Malignancy	CALIBER
Read	B061.00	Malignant neoplasm of tonsillar fossa	Malignancy	CALIBER
Read	B062000	Malignant neoplasm of faucial pillar	Malignancy	CALIBER
Read	B062100	Malignant neoplasm of glossopalatine fold	Malignancy	CALIBER
Read	B062200	Malignant neoplasm of palatoglossal arch	Malignancy	CALIBER
Read	B062300	Malignant neoplasm of palatopharyngeal arch	Malignancy	CALIBER
Read	B062.00	Malignant neoplasm of tonsillar pillar	Malignancy	CALIBER
Read	B062z00	Malignant neoplasm of tonsillar fossa NOS	Malignancy	CALIBER
Read	B063.00	Malignant neoplasm of vallecula	Malignancy	CALIBER
Read	B064000	Malignant neoplasm of epiglottis, free border	Malignancy	CALIBER
Read	B064100	Malignant neoplasm of glossoepiglottic fold	Malignancy	CALIBER
Read	B064.00	Malignant neoplasm of anterior epiglottis	Malignancy	CALIBER
Read	B064z00	Malignant neoplasm of anterior epiglottis NOS	Malignancy	CALIBER
Read	B065.00	Malignant neoplasm of junctional region of epiglottis	Malignancy	CALIBER
Read	B066.00	Malignant neoplasm of lateral wall of oropharynx	Malignancy	CALIBER
Read	B067.00	Malignant neoplasm of posterior wall of oropharynx	Malignancy	CALIBER

Read	B06..00	Malignant neoplasm of oropharynx	Malignancy	CALIBER
Read	B06y.00	Malignant neoplasm of oropharynx, other specified sites	Malignancy	CALIBER
Read	B06yz00	Malignant neoplasm of other specified site of oropharynx NOS	Malignancy	CALIBER
Read	B06z.00	Malignant neoplasm of oropharynx NOS	Malignancy	CALIBER
Read	B070.00	Malignant neoplasm of roof of nasopharynx	Malignancy	CALIBER
Read	B071000	Malignant neoplasm of adenoid	Malignancy	CALIBER
Read	B071100	Malignant neoplasm of pharyngeal tonsil	Malignancy	CALIBER
Read	B071.00	Malignant neoplasm of posterior wall of nasopharynx	Malignancy	CALIBER
Read	B071z00	Malignant neoplasm of posterior wall of nasopharynx NOS	Malignancy	CALIBER
Read	B072000	Malignant neoplasm of pharyngeal recess	Malignancy	CALIBER
Read	B072.00	Malignant neoplasm of lateral wall of nasopharynx	Malignancy	CALIBER
Read	B072z00	Malignant neoplasm of lateral wall of nasopharynx NOS	Malignancy	CALIBER
Read	B073100	Malignant neoplasm of nasopharyngeal soft palate surface	Malignancy	CALIBER
Read	B073200	Malignant neoplasm posterior margin nasal septum and choanae	Malignancy	CALIBER
Read	B073.00	Malignant neoplasm of anterior wall of nasopharynx	Malignancy	CALIBER
Read	B073z00	Malignant neoplasm of anterior wall of nasopharynx NOS	Malignancy	CALIBER
Read	B074.00	Malignant neoplasm, overlapping lesion of nasopharynx	Malignancy	CALIBER
Read	B07..00	Malignant neoplasm of nasopharynx	Malignancy	CALIBER
Read	B07y.00	Malignant neoplasm of other specified site of nasopharynx	Malignancy	CALIBER
Read	B07z.00	Malignant neoplasm of nasopharynx NOS	Malignancy	CALIBER
Read	B080.00	Malignant neoplasm of postcricoid region	Malignancy	CALIBER
Read	B081.00	Malignant neoplasm of pyriform sinus	Malignancy	CALIBER
Read	B082.00	Malignant neoplasm aryepiglottic fold, hypopharyngeal aspect	Malignancy	CALIBER
Read	B083.00	Malignant neoplasm of posterior pharynx	Malignancy	CALIBER
Read	B08..00	Malignant neoplasm of hypopharynx	Malignancy	CALIBER
Read	B08y.00	Malignant neoplasm of other specified hypopharyngeal site	Malignancy	CALIBER
Read	B08z.00	Malignant neoplasm of hypopharynx NOS	Malignancy	CALIBER
Read	B0...00	Malignant neoplasm of lip, oral cavity and pharynx	Malignancy	CALIBER
Read	B0...11	Carcinoma of lip, oral cavity and pharynx	Malignancy	CALIBER
Read	B0z0.00	Malignant neoplasm of pharynx unspecified	Malignancy	CALIBER
Read	B0z1.00	Malignant neoplasm of Waldeyer's ring	Malignancy	CALIBER

Read	B0z2.00	Malignant neoplasm of laryngopharynx	Malignancy	CALIBER
Read	B0z..00	Malig neop other/ill-defined sites lip, oral cavity, pharynx	Malignancy	CALIBER
Read	B0zy.00	Malignant neoplasm of other sites lip, oral cavity, pharynx	Malignancy	CALIBER
Read	B0zz.00	Malignant neoplasm of lip, oral cavity and pharynx NOS	Malignancy	CALIBER
Read	BB5y000	[M]Basal cell adenocarcinoma	Malignancy	CALIBER
Read	Byu0.00	[X]Malignant neoplasm of lip, oral cavity and pharynx	Malignancy	CALIBER
ICD10	C00	Malignant neoplasm of lip	Malignancy	CALIBER
ICD10	C01	Malignant neoplasm of base of tongue	Malignancy	CALIBER
ICD10	C02	Malignant neoplasm of other and unspecified parts of tongue	Malignancy	CALIBER
ICD10	C03	Malignant neoplasm of gum	Malignancy	CALIBER
ICD10	C04	Malignant neoplasm of floor of mouth	Malignancy	CALIBER
ICD10	C05	Malignant neoplasm of palate	Malignancy	CALIBER
ICD10	C06	Malignant neoplasm of other and unspecified parts of mouth	Malignancy	CALIBER
ICD10	C07	Malignant neoplasm of parotid gland	Malignancy	CALIBER
ICD10	C08	Malignant neoplasm of other and unspecified major salivary glands	Malignancy	CALIBER
ICD10	C09	Malignant neoplasm of tonsil	Malignancy	CALIBER
ICD10	C10	Malignant neoplasm of oropharynx	Malignancy	CALIBER
ICD10	C11	Malignant neoplasm of nasopharynx	Malignancy	CALIBER
ICD10	C12	Malignant neoplasm of piriform sinus	Malignancy	CALIBER
ICD10	C13	Malignant neoplasm of hypopharynx	Malignancy	CALIBER
ICD10	C14	Malignant neoplasm of other and ill-defined sites in the lip, oral cavity and pharynx	Malignancy	CALIBER
Read	4M20.00	Lymphoma stage I	Malignancy	CALIBER
Read	4M21.00	Lymphoma stage II	Malignancy	CALIBER
Read	4M22.00	Lymphoma stage III	Malignancy	CALIBER
Read	4M23.00	Lymphoma stage IV	Malignancy	CALIBER
Read	7G03K00	Excision malignant skin tumour	Malignancy	CALIBER
Read	A789500	HIV disease resulting in Kaposi's sarcoma	Malignancy	CALIBER
Read	A789511	HIV disease resulting in Kaposi sarcoma	Malignancy	CALIBER
Read	B120.00	Malignant neoplasm of duodenum	Malignancy	CALIBER
Read	B121.00	Malignant neoplasm of jejunum	Malignancy	CALIBER
Read	B122.00	Malignant neoplasm of ileum	Malignancy	CALIBER
Read	B123.00	Malignant neoplasm of Meckel's diverticulum	Malignancy	CALIBER
Read	B124.00	Malignant neoplasm, overlapping lesion of small intestine	Malignancy	CALIBER

Read	B12..00	Malignant neoplasm of small intestine and duodenum	Malignancy	CALIBER
Read	B12y.00	Malignant neoplasm of other specified site small intestine	Malignancy	CALIBER
Read	B12z.00	Malignant neoplasm of small intestine NOS	Malignancy	CALIBER
Read	B15..00	Malignant neoplasm of liver and intrahepatic bile ducts	Malignancy	CALIBER
Read	B15z.00	Malignant neoplasm of liver and intrahepatic bile ducts NOS	Malignancy	CALIBER
Read	B160.00	Malignant neoplasm of gallbladder	Malignancy	CALIBER
Read	B160.11	Carcinoma gallbladder	Malignancy	CALIBER
Read	B16..00	Malignant neoplasm gallbladder and extrahepatic bile ducts	Malignancy	CALIBER
Read	B16y.00	Malignant neoplasm other gallbladder/extrahepatic bile duct	Malignancy	CALIBER
Read	B16z.00	Malignant neoplasm gallbladder/extrahepatic bile ducts NOS	Malignancy	CALIBER
Read	B180100	Malignant neoplasm of perinephric tissue	Malignancy	CALIBER
Read	B180200	Malignant neoplasm of retrocaecal tissue	Malignancy	CALIBER
Read	B180.00	Malignant neoplasm of retroperitoneum	Malignancy	CALIBER
Read	B180z00	Malignant neoplasm of retroperitoneum NOS	Malignancy	CALIBER
Read	B182.00	Overlapping malign lesion of retroperitoneum and peritoneum	Malignancy	CALIBER
Read	B18..00	Malignant neoplasm of retroperitoneum and peritoneum	Malignancy	CALIBER
Read	B18y100	Malignant neoplasm of mesocaecum	Malignancy	CALIBER
Read	B18y200	Malignant neoplasm of mesorectum	Malignancy	CALIBER
Read	B18y300	Malignant neoplasm of omentum	Malignancy	CALIBER
Read	B18y400	Malignant neoplasm of parietal peritoneum	Malignancy	CALIBER
Read	B18y500	Malignant neoplasm of pelvic peritoneum	Malignancy	CALIBER
Read	B18y600	Malignant neoplasm of the pouch of Douglas	Malignancy	CALIBER
Read	B18y700	Malignant neoplasm of mesentery	Malignancy	CALIBER
Read	B18y.00	Malignant neoplasm of specified parts of peritoneum	Malignancy	CALIBER
Read	B18yz00	Malignant neoplasm of specified parts of peritoneum NOS	Malignancy	CALIBER
Read	B18z.00	Malignant neoplasm of retroperitoneum and peritoneum NOS	Malignancy	CALIBER
Read	B1z0.00	Malignant neoplasm of intestinal tract, part unspecified	Malignancy	CALIBER
Read	B1z1000	Angiosarcoma of spleen	Malignancy	CALIBER
Read	B1z1100	Fibrosarcoma of spleen	Malignancy	CALIBER
Read	B1z1.00	Malignant neoplasm of spleen NEC	Malignancy	CALIBER
Read	B1z1z00	Malignant neoplasm of spleen NOS	Malignancy	CALIBER

Read	B1z2.00	Malignant neoplasm, overlapping lesion of digestive system	Malignancy	CALIBER
Read	B1z..00	Malig neop oth/ill-defined sites digestive tract/peritoneum	Malignancy	CALIBER
Read	B1zy.00	Malignant neoplasm other spec digestive tract and peritoneum	Malignancy	CALIBER
Read	B1zz.00	Malignant neoplasm of digestive tract and peritoneum NOS	Malignancy	CALIBER
Read	B200000	Malignant neoplasm of cartilage of nose	Malignancy	CALIBER
Read	B200100	Malignant neoplasm of nasal conchae	Malignancy	CALIBER
Read	B200200	Malignant neoplasm of septum of nose	Malignancy	CALIBER
Read	B200300	Malignant neoplasm of vestibule of nose	Malignancy	CALIBER
Read	B200.00	Malignant neoplasm of nasal cavities	Malignancy	CALIBER
Read	B200z00	Malignant neoplasm of nasal cavities NOS	Malignancy	CALIBER
Read	B201000	Malignant neoplasm of auditory (Eustachian) tube	Malignancy	CALIBER
Read	B201100	Malignant neoplasm of tympanic cavity	Malignancy	CALIBER
Read	B201200	Malignant neoplasm of tympanic antrum	Malignancy	CALIBER
Read	B201300	Malignant neoplasm of mastoid air cells	Malignancy	CALIBER
Read	B201.00	Malig neop auditory tube, middle ear and mastoid air cells	Malignancy	CALIBER
Read	B201z00	Malig neop auditory tube, middle ear, mastoid air cells NOS	Malignancy	CALIBER
Read	B202.00	Malignant neoplasm of maxillary sinus	Malignancy	CALIBER
Read	B203.00	Malignant neoplasm of ethmoid sinus	Malignancy	CALIBER
Read	B204.00	Malignant neoplasm of frontal sinus	Malignancy	CALIBER
Read	B205.00	Malignant neoplasm of sphenoidal sinus	Malignancy	CALIBER
Read	B206.00	Malignant neoplasm, overlapping lesion of accessory sinuses	Malignancy	CALIBER
Read	B20..00	Malig neop nasal cavities, middle ear and accessory sinuses	Malignancy	CALIBER
Read	B20y.00	Malig neop other site nasal cavity, middle ear and sinuses	Malignancy	CALIBER
Read	B20z.00	Malignant neoplasm of accessory sinus NOS	Malignancy	CALIBER
Read	B210.00	Malignant neoplasm of glottis	Malignancy	CALIBER
Read	B211.00	Malignant neoplasm of supraglottis	Malignancy	CALIBER
Read	B212.00	Malignant neoplasm of subglottis	Malignancy	CALIBER
Read	B213000	Malignant neoplasm of arytenoid cartilage	Malignancy	CALIBER
Read	B213100	Malignant neoplasm of cricoid cartilage	Malignancy	CALIBER
Read	B213200	Malignant neoplasm of cuneiform cartilage	Malignancy	CALIBER
Read	B213300	Malignant neoplasm of thyroid cartilage	Malignancy	CALIBER
Read	B213.00	Malignant neoplasm of laryngeal cartilage	Malignancy	CALIBER
Read	B213z00	Malignant neoplasm of laryngeal cartilage NOS	Malignancy	CALIBER

Read	B214.00	Malignant neoplasm, overlapping lesion of larynx	Malignancy	CALIBER
Read	B215.00	Malignant neoplasm of epiglottis NOS	Malignancy	CALIBER
Read	B21..00	Malignant neoplasm of larynx	Malignancy	CALIBER
Read	B21y.00	Malignant neoplasm of larynx, other specified site	Malignancy	CALIBER
Read	B21z.00	Malignant neoplasm of larynx NOS	Malignancy	CALIBER
Read	B230.00	Malignant neoplasm of parietal pleura	Malignancy	CALIBER
Read	B231.00	Malignant neoplasm of visceral pleura	Malignancy	CALIBER
Read	B23..00	Malignant neoplasm of pleura	Malignancy	CALIBER
Read	B23y.00	Malignant neoplasm of other specified pleura	Malignancy	CALIBER
Read	B23z.00	Malignant neoplasm of pleura NOS	Malignancy	CALIBER
Read	B240.00	Malignant neoplasm of thymus	Malignancy	CALIBER
Read	B241000	Malignant neoplasm of endocardium	Malignancy	CALIBER
Read	B241200	Malignant neoplasm of myocardium	Malignancy	CALIBER
Read	B241300	Malignant neoplasm of pericardium	Malignancy	CALIBER
Read	B241.00	Malignant neoplasm of heart	Malignancy	CALIBER
Read	B241z00	Malignant neoplasm of heart NOS	Malignancy	CALIBER
Read	B242.00	Malignant neoplasm of anterior mediastinum	Malignancy	CALIBER
Read	B243.00	Malignant neoplasm of posterior mediastinum	Malignancy	CALIBER
Read	B24..00	Malignant neoplasm of thymus, heart and mediastinum	Malignancy	CALIBER
Read	B24X.00	Malignant neoplasm of mediastinum, part unspecified	Malignancy	CALIBER
Read	B24y.00	Malig neop of other site of heart, thymus and mediastinum	Malignancy	CALIBER
Read	B24z.00	Malignant neoplasm of heart, thymus and mediastinum NOS	Malignancy	CALIBER
Read	B25..00	Malig neo, overlapping lesion of heart, mediastinum & pleura	Malignancy	CALIBER
Read	B26..00	Malignant neoplasm, overlap lesion of resp & intrathor orgs	Malignancy	CALIBER
Read	B2z0.00	Malig neop of upper respiratory tract, part unspecified	Malignancy	CALIBER
Read	B2z..00	Malig neop other/ill-defined sites resp/intrathoracic organs	Malignancy	CALIBER
Read	B2zy.00	Malignant neoplasm of other site of respiratory tract	Malignancy	CALIBER
Read	B2zz.00	Malignant neoplasm of respiratory tract NOS	Malignancy	CALIBER
Read	B310000	Malignant neoplasm of soft tissue of head	Malignancy	CALIBER
Read	B310100	Malignant neoplasm of soft tissue of face	Malignancy	CALIBER
Read	B310200	Malignant neoplasm of soft tissue of neck	Malignancy	CALIBER

Read	B310300	Malignant neoplasm of cartilage of ear	Malignancy	CALIBER
Read	B310400	Malignant neoplasm of tarsus of eyelid	Malignancy	CALIBER
Read	B310500	Malignant neoplasm soft tissues of cervical spine	Malignancy	CALIBER
Read	B310.00	Malig neop of connective and soft tissue head, face and neck	Malignancy	CALIBER
Read	B310z00	Malig neop connective and soft tissue head, face, neck NOS	Malignancy	CALIBER
Read	B311000	Malignant neoplasm of connective and soft tissue of shoulder	Malignancy	CALIBER
Read	B311100	Malignant neoplasm of connective and soft tissue, upper arm	Malignancy	CALIBER
Read	B311200	Malignant neoplasm of connective and soft tissue of fore-arm	Malignancy	CALIBER
Read	B311300	Malignant neoplasm of connective and soft tissue of hand	Malignancy	CALIBER
Read	B311400	Malignant neoplasm of connective and soft tissue of finger	Malignancy	CALIBER
Read	B311500	Malignant neoplasm of connective and soft tissue of thumb	Malignancy	CALIBER
Read	B311.00	Malig neop connective and soft tissue upper limb/shoulder	Malignancy	CALIBER
Read	B311z00	Malig neop connective soft tissue upper limb/shoulder NOS	Malignancy	CALIBER
Read	B312000	Malignant neoplasm of connective and soft tissue of hip	Malignancy	CALIBER
Read	B312100	Malig neop of connective and soft tissue thigh and upper leg	Malignancy	CALIBER
Read	B312200	Malig neop connective and soft tissue of popliteal space	Malignancy	CALIBER
Read	B312300	Malig neop of connective and soft tissue of lower leg	Malignancy	CALIBER
Read	B312400	Malignant neoplasm of connective and soft tissue of foot	Malignancy	CALIBER
Read	B312500	Malignant neoplasm of connective and soft tissue of toe	Malignancy	CALIBER
Read	B312.00	Malig neop of connective and soft tissue of hip and leg	Malignancy	CALIBER
Read	B312z00	Malig neop connective and soft tissue hip and leg NOS	Malignancy	CALIBER
Read	B313000	Malignant neoplasm of connective and soft tissue of axilla	Malignancy	CALIBER
Read	B313100	Malignant neoplasm of diaphragm	Malignancy	CALIBER
Read	B313200	Malignant neoplasm of great vessels	Malignancy	CALIBER
Read	B313300	Malig neoplasm of connective and soft tissues of thor spine	Malignancy	CALIBER
Read	B313.00	Malignant neoplasm of connective and soft tissue of thorax	Malignancy	CALIBER

Read	B313z00	Malig neop of connective and soft tissue of thorax NOS	Malignancy	CALIBER
Read	B314000	Malig neop of connective and soft tissue of abdominal wall	Malignancy	CALIBER
Read	B314100	Malig neoplasm of connective and soft tissues of lumb spine	Malignancy	CALIBER
Read	B314.00	Malignant neoplasm of connective and soft tissue of abdomen	Malignancy	CALIBER
Read	B314z00	Malig neop of connective and soft tissue of abdomen NOS	Malignancy	CALIBER
Read	B315000	Malignant neoplasm of connective and soft tissue of buttock	Malignancy	CALIBER
Read	B315100	Malig neop of connective and soft tissue of inguinal region	Malignancy	CALIBER
Read	B315200	Malignant neoplasm of connective and soft tissue of perineum	Malignancy	CALIBER
Read	B315300	Malig neopl of connective and soft tissue - sacrum or coccyx	Malignancy	CALIBER
Read	B315.00	Malignant neoplasm of connective and soft tissue of pelvis	Malignancy	CALIBER
Read	B315z00	Malig neop of connective and soft tissue of pelvis NOS	Malignancy	CALIBER
Read	B316.00	Malig neop of connective and soft tissue trunk unspecified	Malignancy	CALIBER
Read	B31y.00	Malig neop connective and soft tissue other specified site	Malignancy	CALIBER
Read	B31z000	Kaposi's sarcoma of soft tissue	Malignancy	CALIBER
Read	B31z.00	Malignant neoplasm of connective and soft tissue, site NOS	Malignancy	CALIBER
Read	B420.00	Choriocarcinoma	Malignancy	CALIBER
Read	B42..00	Malignant neoplasm of placenta	Malignancy	CALIBER
Read	B441.00	Malignant neoplasm of fallopian tube	Malignancy	CALIBER
Read	B442.00	Malignant neoplasm of broad ligament	Malignancy	CALIBER
Read	B443.00	Malignant neoplasm of parametrium	Malignancy	CALIBER
Read	B44..00	Malignant neoplasm of ovary and other uterine adnexa	Malignancy	CALIBER
Read	B44y.00	Malignant neoplasm of other site of uterine adnexa	Malignancy	CALIBER
Read	B44z.00	Malignant neoplasm of uterine adnexa NOS	Malignancy	CALIBER
Read	B450100	Malignant neoplasm of vaginal vault	Malignancy	CALIBER
Read	B450.00	Malignant neoplasm of vagina	Malignancy	CALIBER
Read	B450z00	Malignant neoplasm of vagina NOS	Malignancy	CALIBER
Read	B451000	Malignant neoplasm of greater vestibular (Bartholin's) gland	Malignancy	CALIBER
Read	B451.00	Malignant neoplasm of labia majora	Malignancy	CALIBER
Read	B451z00	Malignant neoplasm of labia majora NOS	Malignancy	CALIBER

Read	B452.00	Malignant neoplasm of labia minora	Malignancy	CALIBER
Read	B453.00	Malignant neoplasm of clitoris	Malignancy	CALIBER
Read	B454.00	Malignant neoplasm of vulva unspecified	Malignancy	CALIBER
Read	B454.11	Primary vulval cancer	Malignancy	CALIBER
Read	B45..00	Malig neop of other and unspecified female genital organs	Malignancy	CALIBER
Read	B45X.00	Malignant neoplasm/overlapping lesion/feml genital organs	Malignancy	CALIBER
Read	B45y000	Malignant neoplasm of overlapping lesion of vulva	Malignancy	CALIBER
Read	B45y.00	Malignant neoplasm of other specified female genital organ	Malignancy	CALIBER
Read	B45z.00	Malignant neoplasm of female genital organ NOS	Malignancy	CALIBER
Read	B480.00	Malignant neoplasm of prepuce (foreskin)	Malignancy	CALIBER
Read	B481.00	Malignant neoplasm of glans penis	Malignancy	CALIBER
Read	B482.00	Malignant neoplasm of body of penis	Malignancy	CALIBER
Read	B483.00	Malignant neoplasm of penis, part unspecified	Malignancy	CALIBER
Read	B484.00	Malignant neoplasm of epididymis	Malignancy	CALIBER
Read	B485.00	Malignant neoplasm of spermatic cord	Malignancy	CALIBER
Read	B486.00	Malignant neoplasm of scrotum	Malignancy	CALIBER
Read	B487.00	Malignant neoplasm, overlapping lesion of penis	Malignancy	CALIBER
Read	B48y000	Malignant neoplasm of seminal vesicle	Malignancy	CALIBER
Read	B48y100	Malignant neoplasm of tunica vaginalis	Malignancy	CALIBER
Read	B48y200	Malignant neoplasm, overlapping lesion male genital orgs	Malignancy	CALIBER
Read	B48y.00	Malignant neoplasm of other male genital organ	Malignancy	CALIBER
Read	B48yz00	Malignant neoplasm of other male genital organ NOS	Malignancy	CALIBER
Read	B48z.00	Malignant neoplasm of penis and other male genital organ NOS	Malignancy	CALIBER
Read	B4A3.00	Malignant neoplasm of urethra	Malignancy	CALIBER
Read	B4A4.00	Malignant neoplasm of paraurethral glands	Malignancy	CALIBER
Read	B4A..00	Malig neop of kidney and other unspecified urinary organs	Malignancy	CALIBER
Read	B4Ay000	Malignant neoplasm of overlapping lesion of urinary organs	Malignancy	CALIBER
Read	B4Ay.00	Malignant neoplasm of other urinary organs	Malignancy	CALIBER
Read	B4Az.00	Malignant neoplasm of kidney or urinary organs NOS	Malignancy	CALIBER
Read	B500000	Malignant neoplasm of ciliary body	Malignancy	CALIBER
Read	B500100	Malignant neoplasm of iris	Malignancy	CALIBER

Read	B500200	Malignant neoplasm of crystalline lens	Malignancy	CALIBER
Read	B500.00	Malig neop eyeball excl conjunctiva, cornea, retina, choroid	Malignancy	CALIBER
Read	B500z00	Malignant neoplasm of eyeball NOS	Malignancy	CALIBER
Read	B501000	Malignant neoplasm of connective tissue of orbit	Malignancy	CALIBER
Read	B501.00	Malignant neoplasm of orbit	Malignancy	CALIBER
Read	B501z00	Malignant neoplasm of orbit NOS	Malignancy	CALIBER
Read	B502.00	Malignant neoplasm of lacrimal gland	Malignancy	CALIBER
Read	B503.00	Malignant neoplasm of conjunctiva	Malignancy	CALIBER
Read	B504.00	Malignant neoplasm of cornea	Malignancy	CALIBER
Read	B505.00	Malignant neoplasm of retina	Malignancy	CALIBER
Read	B506.00	Malignant neoplasm of choroid	Malignancy	CALIBER
Read	B507000	Malignant neoplasm of lacrimal sac	Malignancy	CALIBER
Read	B507100	Malignant neoplasm of nasolacrimal duct	Malignancy	CALIBER
Read	B507.00	Malignant neoplasm of lacrimal duct	Malignancy	CALIBER
Read	B508.00	Malignant neoplasm, overlapping lesion of eye and adnexa	Malignancy	CALIBER
Read	B50..00	Malignant neoplasm of eye	Malignancy	CALIBER
Read	B50y.00	Malignant neoplasm of other specified site of eye	Malignancy	CALIBER
Read	B50z.00	Malignant neoplasm of eye NOS	Malignancy	CALIBER
Read	B524000	Malignant neoplasm of peripheral nerves of head, face & neck	Malignancy	CALIBER
Read	B524100	Malignant neoplasm of peripheral nerve,upp limb,incl should	Malignancy	CALIBER
Read	B524200	Malignant neoplasm of peripheral nerve of low limb, incl hip	Malignancy	CALIBER
Read	B524300	Malignant neoplasm of peripheral nerve of thorax	Malignancy	CALIBER
Read	B524400	Malignant neoplasm of peripheral nerve of abdomen	Malignancy	CALIBER
Read	B524500	Malignant neoplasm of peripheral nerve of pelvis	Malignancy	CALIBER
Read	B524600	Malignant neoplasm,overlap lesion periph nerve & auton ns	Malignancy	CALIBER
Read	B524.00	Malig neopl peripheral nerves and autonomic nervous system	Malignancy	CALIBER
Read	B524W00	Mal neoplasm/periph nerves+autonomic nervous system,unspc	Malignancy	CALIBER
Read	B52y.00	Malignant neoplasm of other specified part of nervous system	Malignancy	CALIBER
Read	B52z.00	Malignant neoplasm of nervous system NOS	Malignancy	CALIBER
Read	B52..00	Malig neop of other and unspecified parts of nervous system	Malignancy	CALIBER
Read	B540000	Malignant neoplasm of adrenal cortex	Malignancy	CALIBER

Read	B540100	Malignant neoplasm of adrenal medulla	Malignancy	CALIBER
Read	B540.00	Malignant neoplasm of adrenal gland	Malignancy	CALIBER
Read	B540.11	Phaeochromocytoma	Malignancy	CALIBER
Read	B540z00	Malignant neoplasm of adrenal gland NOS	Malignancy	CALIBER
Read	B541.00	Malignant neoplasm of parathyroid gland	Malignancy	CALIBER
Read	B54X.00	Malignant neoplasm-pluriglandular involvement,unspecified	Malignancy	CALIBER
Read	B54y.00	Malignant neoplasm of other specified endocrine gland	Malignancy	CALIBER
Read	B54z.00	Malig neop of endocrine gland or related structure NOS	Malignancy	CALIBER
Read	B550000	Malignant neoplasm of head NOS	Malignancy	CALIBER
Read	B550100	Malignant neoplasm of cheek NOS	Malignancy	CALIBER
Read	B550200	Malignant neoplasm of nose NOS	Malignancy	CALIBER
Read	B550300	Malignant neoplasm of jaw NOS	Malignancy	CALIBER
Read	B550400	Malignant neoplasm of neck NOS	Malignancy	CALIBER
Read	B550500	Malignant neoplasm of supraclavicular fossa NOS	Malignancy	CALIBER
Read	B550.00	Malignant neoplasm of head, neck and face	Malignancy	CALIBER
Read	B550z00	Malignant neoplasm of head, neck and face NOS	Malignancy	CALIBER
Read	B551000	Malignant neoplasm of axilla NOS	Malignancy	CALIBER
Read	B551100	Malignant neoplasm of chest wall NOS	Malignancy	CALIBER
Read	B551200	Malignant neoplasm of intrathoracic site NOS	Malignancy	CALIBER
Read	B551.00	Malignant neoplasm of thorax	Malignancy	CALIBER
Read	B551z00	Malignant neoplasm of thorax NOS	Malignancy	CALIBER
Read	B552.00	Malignant neoplasm of abdomen	Malignancy	CALIBER
Read	B553000	Malignant neoplasm of inguinal region NOS	Malignancy	CALIBER
Read	B553100	Malignant neoplasm of presacral region	Malignancy	CALIBER
Read	B553200	Malignant neoplasm of sacrococcygeal region	Malignancy	CALIBER
Read	B553.00	Malignant neoplasm of pelvis	Malignancy	CALIBER
Read	B553z00	Malignant neoplasm of pelvis NOS	Malignancy	CALIBER
Read	B554.00	Malignant neoplasm of upper limb NOS	Malignancy	CALIBER
Read	B555.00	Malignant neoplasm of lower limb NOS	Malignancy	CALIBER
Read	B55y000	Malignant neoplasm of back NOS	Malignancy	CALIBER
Read	B55y100	Malignant neoplasm of trunk NOS	Malignancy	CALIBER
Read	B55y200	Malignant neoplasm of flank NOS	Malignancy	CALIBER
Read	B55y.00	Malignant neoplasm of other specified sites	Malignancy	CALIBER
Read	B55yz00	Malignant neoplasm of specified site NOS	Malignancy	CALIBER
Read	B55z.00	Malignant neoplasm of other and ill defined site NOS	Malignancy	CALIBER
Read	B591.00	Other malignant neoplasm NOS	Malignancy	CALIBER

Read	B592X00	Kaposi's sarcoma of multiple organs	Malignancy	CALIBER
Read	B593.00	Primary malignant neoplasm of unknown site	Malignancy	CALIBER
Read	B595.00	Malignant tumour of unknown origin	Malignancy	CALIBER
Read	B59..00	Malignant neoplasm of unspecified site	Malignancy	CALIBER
Read	B59z.00	Malignant neoplasm of unspecified site NOS	Malignancy	CALIBER
Read	B59zX00	Kaposi's sarcoma, unspecified	Malignancy	CALIBER
Read	B5...00	Malignant neoplasm of other and unspecified sites	Malignancy	CALIBER
Read	B5...11	Carcinoma of other and unspecified sites	Malignancy	CALIBER
Read	B5y..00	Malignant neoplasm of other and unspecified site OS	Malignancy	CALIBER
Read	B5z..00	Malignant neoplasm of other and unspecified site NOS	Malignancy	CALIBER
Read	B623000	Malignant histiocytosis of unspecified site	Malignancy	CALIBER
Read	B623100	Malignant histiocytosis of lymph nodes head, face and neck	Malignancy	CALIBER
Read	B623300	Malignant histiocytosis of intra-abdominal lymph nodes	Malignancy	CALIBER
Read	B623.00	Malignant histiocytosis	Malignancy	CALIBER
Read	B623z00	Malignant histiocytosis NOS	Malignancy	CALIBER
Read	B625000	Letterer-Siwe disease of unspecified sites	Malignancy	CALIBER
Read	B625200	Letterer-Siwe disease of intrathoracic lymph nodes	Malignancy	CALIBER
Read	B625800	Letterer-Siwe disease of lymph nodes of multiple sites	Malignancy	CALIBER
Read	B625.00	Letterer-Siwe disease	Malignancy	CALIBER
Read	B625.11	Histiocytosis X (acute, progressive)	Malignancy	CALIBER
Read	B625z00	Letterer-Siwe disease NOS	Malignancy	CALIBER
Read	B626000	Mast cell malignancy of unspecified site	Malignancy	CALIBER
Read	B626500	Mast cell malignancy of lymph nodes inguinal region and leg	Malignancy	CALIBER
Read	B626800	Mast cell malignancy of lymph nodes of multiple sites	Malignancy	CALIBER
Read	B626.00	Malignant mast cell tumours	Malignancy	CALIBER
Read	B626z00	Malignant mast cell tumour NOS	Malignancy	CALIBER
Read	B62x500	Malignant immunoproliferative small intestinal disease	Malignancy	CALIBER
Read	B62x600	True histiocytic lymphoma	Malignancy	CALIBER
Read	B62z000	Unspec malig neop lymphoid/histiocytic of unspecified site	Malignancy	CALIBER
Read	B62z100	Unspec malig neop lymphoid/histiocytic lymph node head/neck	Malignancy	CALIBER
Read	B62z200	Unspec malig neop lymphoid/histiocytic of intrathoracic node	Malignancy	CALIBER

Read	B62z300	Unspec malig neop lymphoid/histiocytic intra-abdominal nodes	Malignancy	CALIBER
Read	B62z400	Unspec malig neop lymphoid/histiocytic lymph node axilla/arm	Malignancy	CALIBER
Read	B62z500	Unspec malig neop lymphoid/histiocytic nodes inguinal/leg	Malignancy	CALIBER
Read	B62z600	Unspec malig neop lymphoid/histiocytic of intrapelvic nodes	Malignancy	CALIBER
Read	B62z800	Unspec malig neop lymphoid/histiocytic of multiple sites	Malignancy	CALIBER
Read	B62z.00	Malignant neoplasms of lymphoid and histiocytic tissue NOS	Malignancy	CALIBER
Read	B62zz00	Lymphoid and histiocytic malignancy NOS	Malignancy	CALIBER
Read	B62zz11	Immunoproliferative neoplasm	Malignancy	CALIBER
Read	B63y.00	Other immunoproliferative neoplasms	Malignancy	CALIBER
Read	B63z.00	Immunoproliferative neoplasm or myeloma NOS	Malignancy	CALIBER
Read	B6y..00	Malignant neoplasm lymphatic or haematopoietic tissue OS	Malignancy	CALIBER
Read	B6z0.00	Kaposi's sarcoma of lymph nodes	Malignancy	CALIBER
Read	B6z..00	Malignant neoplasm lymphatic or haematopoietic tissue NOS	Malignancy	CALIBER
Read	BB5h100	[M]Adrenal cortical carcinoma	Malignancy	CALIBER
Read	BB57.00	[M]Adenocarcinoma, intestinal type	Malignancy	CALIBER
Read	BBcC.00	[M]Aesthesioneuroblastoma	Malignancy	CALIBER
Read	BBcC.11	[M]Olfactory neuroblastoma	Malignancy	CALIBER
Read	BBcD.11	[M]Olfactory neuroepithelioma	Malignancy	CALIBER
Read	BBm4.00	[M]True histiocytic lymphoma	Malignancy	CALIBER
Read	BBT1.00	[M]Haemangiosarcoma	Malignancy	CALIBER
Read	BBT7100	[M]Haemangioendothelioma, malignant	Malignancy	CALIBER
Read	Byu1200	[X]Malignant neoplasm of intestinal tract, part unspecified	Malignancy	CALIBER
Read	Byu1300	[X]Malignant neoplsm/ill-defin sites within digestive system	Malignancy	CALIBER
Read	Byu2400	[X]Malignant neoplasm/ill-defined sites within resp system	Malignancy	CALIBER
Read	Byu2500	[X]Malignant neoplasm of mediastinum, part unspecified	Malignancy	CALIBER
Read	Byu4.00	[X]Melanoma and other malignant neoplasms of skin	Malignancy	CALIBER
Read	Byu5300	[X]Kaposi's sarcoma, unspecified	Malignancy	CALIBER
Read	Byu5400	[X]Malignant neoplasm/peripheral nerves of trunk,unspecified	Malignancy	CALIBER
Read	Byu5500	[X]Mal neoplasm/overlap les/periph nerv+autonomic nerv systm	Malignancy	CALIBER

Read	Byu5700	[X]Malignant neoplasm of peritoneum, unspecified	Malignancy	CALIBER
Read	Byu5800	[X]Mal neoplasm/connective+soft tissue of trunk,unspecified	Malignancy	CALIBER
Read	Byu5900	[X]Malignant neoplasm/connective + soft tissue,unspecified	Malignancy	CALIBER
Read	Byu5B00	[X]Kaposi's sarcoma of other sites	Malignancy	CALIBER
Read	Byu7000	[X]Malignant neoplasm of uterine adnexa, unspecified	Malignancy	CALIBER
Read	Byu7100	[X]Malignant neoplasm/other specified female genital organs	Malignancy	CALIBER
Read	Byu7300	[X]Malignant neoplasm of female genital organ, unspecified	Malignancy	CALIBER
Read	Byu8000	[X]Malignant neoplasm/other specified male genital organs	Malignancy	CALIBER
Read	Byu8200	[X]Malignant neoplasm of male genital organ, unspecified	Malignancy	CALIBER
Read	Byu9000	[X]Malignant neoplasm of urinary organ, unspecified	Malignancy	CALIBER
Read	ByuB100	[X]Malignant neoplasm of endocrine gland, unspecified	Malignancy	CALIBER
Read	ByuB.00	[X]Malignant neoplasm of thyroid and other endocrine glands	Malignancy	CALIBER
Read	ByuC000	[X]Malignant neoplasm of other specified sites	Malignancy	CALIBER
Read	ByuC100	[X]Malignant neoplasm/overlap lesion/other+ill-defined sites	Malignancy	CALIBER
Read	ByuC600	[X]2ndry malignant neoplasm/oth+unspec parts/nervous system	Malignancy	CALIBER
Read	ByuC800	[X]Malignant neoplasm without specification of site	Malignancy	CALIBER
Read	ByuC.00	[X]Malignant neoplasm of ill-defined, secondary and unspeci	Malignancy	CALIBER
Read	ByuD400	[X]Other malignant immunoproliferative diseases	Malignancy	CALIBER
Read	ByuDA00	[X]Oth spcf mal neoplsm/lymphoid,haematopoietic+rltd tissue	Malignancy	CALIBER
Read	ByuDB00	[X]Mal neoplasm/lymphoid,haematopoietic+related tissu,unspcf	Malignancy	CALIBER
Read	C37y000	Hand - Schuller - Christian disease	Malignancy	CALIBER
Read	C37y100	Eosinophilic granuloma	Malignancy	CALIBER
Read	C37y500	Histiocytosis X , chronic	Malignancy	CALIBER
Read	C37y600	Histiocytosis X , unspecified	Malignancy	CALIBER
ICD10	C17	Malignant neoplasm of small intestine	Malignancy	CALIBER
ICD10	C23	Malignant neoplasm of gallbladder	Malignancy	CALIBER

ICD10	C26.0	Malignant neoplasm: Intestinal tract, part unspecified	Malignancy	CALIBER
ICD10	C26.1	Malignant neoplasm: Spleen	Malignancy	CALIBER
ICD10	C26.8	Malignant neoplasm: Overlapping lesion of digestive system	Malignancy	CALIBER
ICD10	C26.9	Malignant neoplasm: Ill-defined sites within the digestive system	Malignancy	CALIBER
ICD10	C30.0	Malignant neoplasm: Nasal cavity	Malignancy	CALIBER
ICD10	C30.1	Malignant neoplasm: Middle ear	Malignancy	CALIBER
ICD10	C31	Malignant neoplasm of accessory sinuses	Malignancy	CALIBER
ICD10	C32	Malignant neoplasm of larynx	Malignancy	CALIBER
ICD10	C37	Malignant neoplasm of thymus	Malignancy	CALIBER
ICD10	C38.0	Malignant neoplasm: Heart	Malignancy	CALIBER
ICD10	C38.1	Malignant neoplasm: Anterior mediastinum	Malignancy	CALIBER
ICD10	C38.2	Malignant neoplasm: Posterior mediastinum	Malignancy	CALIBER
ICD10	C38.3	Malignant neoplasm: Mediastinum, part unspecified	Malignancy	CALIBER
ICD10	C38.4	Malignant neoplasm: Pleura	Malignancy	CALIBER
ICD10	C38.8	Overlapping lesion of heart, mediastinum and pleura	Malignancy	CALIBER
ICD10	C39.0	Malignant neoplasm: Upper respiratory tract, part unspecified	Malignancy	CALIBER
ICD10	C39.8	Malignant neoplasm: Overlapping lesion of respiratory and intrathoracic organs	Malignancy	CALIBER
ICD10	C39.9	Malignant neoplasm: Ill-defined sites within the respiratory system	Malignancy	CALIBER
ICD10	C46	Kaposi sarcoma	Malignancy	CALIBER
ICD10	C47	Malignant neoplasm of peripheral nerves and autonomic nervous system	Malignancy	CALIBER
ICD10	C48	Malignant neoplasm of retroperitoneum and peritoneum	Malignancy	CALIBER
ICD10	C49	Malignant neoplasm of other connective and soft tissue	Malignancy	CALIBER
ICD10	C51	Malignant neoplasm of vulva	Malignancy	CALIBER
ICD10	C52	Malignant neoplasm of vagina	Malignancy	CALIBER
ICD10	C57	Malignant neoplasm of other and unspecified female genital organs	Malignancy	CALIBER
ICD10	C58	Malignant neoplasm of placenta	Malignancy	CALIBER
ICD10	C60	Malignant neoplasm of penis	Malignancy	CALIBER
ICD10	C63	Malignant neoplasm of other and unspecified male genital organs	Malignancy	CALIBER
ICD10	C68	Malignant neoplasm of other and unspecified urinary organs	Malignancy	CALIBER
ICD10	C69	Malignant neoplasm of eye and adnexa	Malignancy	CALIBER
ICD10	C74	Malignant neoplasm of adrenal gland	Malignancy	CALIBER

ICD10	C75.0	Malignant neoplasm: Parathyroid gland	Malignancy	CALIBER
ICD10	C75.8	Malignant neoplasm: Pluriglandular involvement, unspecified	Malignancy	CALIBER
ICD10	C75.9	Malignant neoplasm: Endocrine gland, unspecified	Malignancy	CALIBER
ICD10	C76	Malignant neoplasm of other and ill-defined sites	Malignancy	CALIBER
ICD10	C80	Malignant neoplasm without specification of site	Malignancy	CALIBER
ICD10	C88.3	Immunoproliferative small intestinal disease	Malignancy	CALIBER
ICD10	C88.4	Extranodal marginal zone B-cell lymphoma of mucosa-associated lymphoid tissue [MALT-lymphoma]	Malignancy	CALIBER
ICD10	C88.7	Other malignant immunoproliferative diseases	Malignancy	CALIBER
ICD10	C88.9	Malignant immunoproliferative disease, unspecified	Malignancy	CALIBER
ICD10	C96	Other and unspecified malignant neoplasms of lymphoid, haematopoietic and related tissue	Malignancy	CALIBER
Read	B440.00	Malignant neoplasm of ovary	Malignancy	CALIBER
Read	B440.11	Cancer of ovary	Malignancy	CALIBER
Read	BB5j200	[M]Endometrioid carcinoma	Malignancy	CALIBER
Read	BB5j500	[M]Endometrioid adenofibroma, malignant	Malignancy	CALIBER
Read	BBQA100	[M]Struma ovarii, malignant	Malignancy	CALIBER
Read	D212000	Anaemia in ovarian carcinoma	Malignancy	CALIBER
ICD10	C56	Malignant neoplasm of ovary	Malignancy	CALIBER
Read	B170.00	Malignant neoplasm of head of pancreas	Malignancy	CALIBER
Read	B171.00	Malignant neoplasm of body of pancreas	Malignancy	CALIBER
Read	B172.00	Malignant neoplasm of tail of pancreas	Malignancy	CALIBER
Read	B173.00	Malignant neoplasm of pancreatic duct	Malignancy	CALIBER
Read	B174.00	Malignant neoplasm of Islets of Langerhans	Malignancy	CALIBER
Read	B175.00	Malignant neoplasm, overlapping lesion of pancreas	Malignancy	CALIBER
Read	B17..00	Malignant neoplasm of pancreas	Malignancy	CALIBER
Read	B17y000	Malignant neoplasm of ectopic pancreatic tissue	Malignancy	CALIBER
Read	B17y.00	Malignant neoplasm of other specified sites of pancreas	Malignancy	CALIBER
Read	B17yz00	Malignant neoplasm of specified site of pancreas NOS	Malignancy	CALIBER
Read	B17z.00	Malignant neoplasm of pancreas NOS	Malignancy	CALIBER
Read	BB5B100	[M]Islet cell carcinoma	Malignancy	CALIBER
Read	BB5B300	[M]Insulinoma, malignant	Malignancy	CALIBER

Read	BB5B500	[M]Glucagonoma, malignant	Malignancy	CALIBER
Read	BB5B600	[M]Mixed islet cell and exocrine adenocarcinoma	Malignancy	CALIBER
Read	BB5C100	[M]Gastrinoma, malignant	Malignancy	CALIBER
ICD10	C25	Malignant neoplasm of pancreas	Malignancy	CALIBER
Read	4M00.00	Gleason prostate grade 2-4 (low)	Malignancy	CALIBER
Read	4M01.00	Gleason prostate grade 5-7 (medium)	Malignancy	CALIBER
Read	4M02.00	Gleason prostate grade 8-10 (high)	Malignancy	CALIBER
Read	4M0..00	Gleason grading of prostate cancer	Malignancy	CALIBER
Read	7B20000	Radical cystoprostatourethrectomy	Malignancy	CALIBER
Read	7B20200	Radical cystoprostatectomy	Malignancy	CALIBER
Read	7B36000	Radical prostatectomy - unspecified excision of pelvic nodes	Malignancy	CALIBER
Read	7B36500	Radical prostatectomy without pelvic node excision	Malignancy	CALIBER
Read	7B36600	Radical prostatectomy with pelvic node sampling	Malignancy	CALIBER
Read	7B36700	Radical prostatectomy with pelvic lymphadenectomy	Malignancy	CALIBER
Read	B46..00	Malignant neoplasm of prostate	Malignancy	CALIBER
ICD10	C61	Malignant neoplasm of prostate	Malignancy	CALIBER
Read	B110000	Malignant neoplasm of cardiac orifice of stomach	Malignancy	CALIBER
Read	B110100	Malignant neoplasm of cardio-oesophageal junction of stomach	Malignancy	CALIBER
Read	B110111	Malignant neoplasm of gastro-oesophageal junction	Malignancy	CALIBER
Read	B110.00	Malignant neoplasm of cardia of stomach	Malignancy	CALIBER
Read	B110z00	Malignant neoplasm of cardia of stomach NOS	Malignancy	CALIBER
Read	B111000	Malignant neoplasm of prepylorus of stomach	Malignancy	CALIBER
Read	B111100	Malignant neoplasm of pyloric canal of stomach	Malignancy	CALIBER
Read	B111.00	Malignant neoplasm of pylorus of stomach	Malignancy	CALIBER
Read	B111z00	Malignant neoplasm of pylorus of stomach NOS	Malignancy	CALIBER
Read	B112.00	Malignant neoplasm of pyloric antrum of stomach	Malignancy	CALIBER
Read	B113.00	Malignant neoplasm of fundus of stomach	Malignancy	CALIBER
Read	B114.00	Malignant neoplasm of body of stomach	Malignancy	CALIBER
Read	B115.00	Malignant neoplasm of lesser curve of stomach unspecified	Malignancy	CALIBER
Read	B116.00	Malignant neoplasm of greater curve of stomach unspecified	Malignancy	CALIBER

Read	B117.00	Malignant neoplasm, overlapping lesion of stomach	Malignancy	CALIBER
Read	B118.00	Siewert type II adenocarcinoma	Malignancy	CALIBER
Read	B119.00	Siewert type III adenocarcinoma	Malignancy	CALIBER
Read	B11..00	Malignant neoplasm of stomach	Malignancy	CALIBER
Read	B11y000	Malignant neoplasm of anterior wall of stomach NEC	Malignancy	CALIBER
Read	B11y100	Malignant neoplasm of posterior wall of stomach NEC	Malignancy	CALIBER
Read	B11y.00	Malignant neoplasm of other specified site of stomach	Malignancy	CALIBER
Read	B11yz00	Malignant neoplasm of other specified site of stomach NOS	Malignancy	CALIBER
Read	B11z.00	Malignant neoplasm of stomach NOS	Malignancy	CALIBER
ICD10	C16	Malignant neoplasm of stomach	Malignancy	CALIBER
Read	B470200	Seminoma of undescended testis	Malignancy	CALIBER
Read	B470300	Teratoma of undescended testis	Malignancy	CALIBER
Read	B470.00	Malignant neoplasm of undescended testis	Malignancy	CALIBER
Read	B470z00	Malignant neoplasm of undescended testis NOS	Malignancy	CALIBER
Read	B471000	Seminoma of descended testis	Malignancy	CALIBER
Read	B471100	Teratoma of descended testis	Malignancy	CALIBER
Read	B471.00	Malignant neoplasm of descended testis	Malignancy	CALIBER
Read	B471z00	Malignant neoplasm of descended testis NOS	Malignancy	CALIBER
Read	B47..00	Malignant neoplasm of testis	Malignancy	CALIBER
Read	B47z.00	Malignant neoplasm of testis NOS	Malignancy	CALIBER
Read	B47z.11	Seminoma of testis	Malignancy	CALIBER
Read	B47z.12	Teratoma of testis	Malignancy	CALIBER
ICD10	C62	Malignant neoplasm of testis	Malignancy	CALIBER
Read	B53..00	Malignant neoplasm of thyroid gland	Malignancy	CALIBER
ICD10	C73	Malignant neoplasm of thyroid gland	Malignancy	CALIBER
Read	B40..00	Malignant neoplasm of uterus, part unspecified	Malignancy	CALIBER
Read	B430000	Malignant neoplasm of cornu of corpus uteri	Malignancy	CALIBER
Read	B430100	Malignant neoplasm of fundus of corpus uteri	Malignancy	CALIBER
Read	B430200	Malignant neoplasm of endometrium of corpus uteri	Malignancy	CALIBER
Read	B430211	Malignant neoplasm of endometrium	Malignancy	CALIBER
Read	B430300	Malignant neoplasm of myometrium of corpus uteri	Malignancy	CALIBER
Read	B430.00	Malignant neoplasm of corpus uteri, excluding isthmus	Malignancy	CALIBER

Read	B430z00	Malignant neoplasm of corpus uteri NOS	Malignancy	CALIBER
Read	B431000	Malignant neoplasm of lower uterine segment	Malignancy	CALIBER
Read	B431.00	Malignant neoplasm of isthmus of uterine body	Malignancy	CALIBER
Read	B431z00	Malignant neoplasm of isthmus of uterine body NOS	Malignancy	CALIBER
Read	B432.00	Malignant neoplasm of overlapping lesion of corpus uteri	Malignancy	CALIBER
Read	B43..00	Malignant neoplasm of body of uterus	Malignancy	CALIBER
Read	B43y.00	Malignant neoplasm of other site of uterine body	Malignancy	CALIBER
Read	B43z.00	Malignant neoplasm of body of uterus NOS	Malignancy	CALIBER
Read	ZV10417	[V]Personal history of malignant neoplasm of uterine body	Malignancy	CALIBER
ICD10	C54	Malignant neoplasm of corpus uteri	Malignancy	CALIBER
ICD10	C55	Malignant neoplasm of uterus, part unspecified	Malignancy	CALIBER
Read	B151000	Malignant neoplasm of interlobular bile ducts	Malignancy	CALIBER
Read	B151200	Malignant neoplasm of intrahepatic biliary passages	Malignancy	CALIBER
Read	B151400	Malignant neoplasm of intrahepatic gall duct	Malignancy	CALIBER
Read	B151.00	Malignant neoplasm of intrahepatic bile ducts	Malignancy	CALIBER
Read	B151z00	Malignant neoplasm of intrahepatic bile ducts NOS	Malignancy	CALIBER
Read	B161000	Malignant neoplasm of cystic duct	Malignancy	CALIBER
Read	B161100	Malignant neoplasm of hepatic duct	Malignancy	CALIBER
Read	B161200	Malignant neoplasm of common bile duct	Malignancy	CALIBER
Read	B161211	Carcinoma common bile duct	Malignancy	CALIBER
Read	B161300	Malignant neoplasm of sphincter of Oddi	Malignancy	CALIBER
Read	B161.00	Malignant neoplasm of extrahepatic bile ducts	Malignancy	CALIBER
Read	B161z00	Malignant neoplasm of extrahepatic bile ducts NOS	Malignancy	CALIBER
Read	B162.00	Malignant neoplasm of ampulla of Vater	Malignancy	CALIBER
Read	B163.00	Malignant neoplasm, overlapping lesion of biliary tract	Malignancy	CALIBER
Read	BB5D100	[M]Cholangiocarcinoma	Malignancy	CALIBER
Read	BB5D111	[M]Bile duct carcinoma	Malignancy	CALIBER
Read	BB5D300	[M]Bile duct cystadenocarcinoma	Malignancy	CALIBER
Read	BB5D700	[M]Combined hepatocellular carcinoma and cholangiocarcinoma	Malignancy	CALIBER
Read	BB5D711	[M]Hepatocholangiocarcinoma	Malignancy	CALIBER

ICD10	C22.1	Malignant neoplasm: Intrahepatic bile duct carcinoma	Malignancy	CALIBER
ICD10	C24	Malignant neoplasm of other and unspecified parts of biliary tract	Malignancy	CALIBER
Read	B130.00	Malignant neoplasm of hepatic flexure of colon	Malignancy	CALIBER
Read	B131.00	Malignant neoplasm of transverse colon	Malignancy	CALIBER
Read	B132.00	Malignant neoplasm of descending colon	Malignancy	CALIBER
Read	B133.00	Malignant neoplasm of sigmoid colon	Malignancy	CALIBER
Read	B134.00	Malignant neoplasm of caecum	Malignancy	CALIBER
Read	B134.11	Carcinoma of caecum	Malignancy	CALIBER
Read	B135.00	Malignant neoplasm of appendix	Malignancy	CALIBER
Read	B136.00	Malignant neoplasm of ascending colon	Malignancy	CALIBER
Read	B137.00	Malignant neoplasm of splenic flexure of colon	Malignancy	CALIBER
Read	B138.00	Malignant neoplasm, overlapping lesion of colon	Malignancy	CALIBER
Read	B139.00	Hereditary nonpolyposis colon cancer	Malignancy	CALIBER
Read	B13..00	Malignant neoplasm of colon	Malignancy	CALIBER
Read	B13y.00	Malignant neoplasm of other specified sites of colon	Malignancy	CALIBER
Read	B13z.00	Malignant neoplasm of colon NOS	Malignancy	CALIBER
Read	B13z.11	Colonic cancer	Malignancy	CALIBER
Read	B140.00	Malignant neoplasm of rectosigmoid junction	Malignancy	CALIBER
Read	B141.00	Malignant neoplasm of rectum	Malignancy	CALIBER
Read	B141.11	Carcinoma of rectum	Malignancy	CALIBER
Read	B141.12	Rectal carcinoma	Malignancy	CALIBER
Read	B142000	Malignant neoplasm of cloacogenic zone	Malignancy	CALIBER
Read	B142.00	Malignant neoplasm of anal canal	Malignancy	CALIBER
Read	B142.11	Anal carcinoma	Malignancy	CALIBER
Read	B143.00	Malignant neoplasm of anus unspecified	Malignancy	CALIBER
Read	B14..00	Malignant neoplasm of rectum, rectosigmoid junction and anus	Malignancy	CALIBER
Read	B14y.00	Malig neop other site rectum, rectosigmoid junction and anus	Malignancy	CALIBER
Read	B14z.00	Malignant neoplasm rectum,rectosigmoid junction and anus NOS	Malignancy	CALIBER
Read	B1z0.11	Cancer of bowel	Malignancy	CALIBER
ICD10	C18	Malignant neoplasm of colon	Malignancy	CALIBER
ICD10	C19	Malignant neoplasm of rectosigmoid junction	Malignancy	CALIBER
ICD10	C20	Malignant neoplasm of rectum	Malignancy	CALIBER
ICD10	C21	Malignant neoplasm of anus and anal canal	Malignancy	CALIBER

Read	B587.00	Secondary malignant neoplasm of adrenal gland	Malignancy	CALIBER
ICD10	C79.7	Secondary malignant neoplasm of adrenal gland	Malignancy	CALIBER
Read	B585000	Pathological fracture due to metastatic bone disease	Malignancy	CALIBER
Read	B585.00	Secondary malignant neoplasm of bone and bone marrow	Malignancy	CALIBER
ICD10	C79.5	Secondary malignant neoplasm of bone and bone marrow	Malignancy	CALIBER
Read	B574000	Secondary malignant neoplasm of duodenum	Malignancy	CALIBER
Read	B574100	Secondary malignant neoplasm of jejunum	Malignancy	CALIBER
Read	B574200	Secondary malignant neoplasm of ileum	Malignancy	CALIBER
Read	B574.00	Secondary malignant neoplasm of small intestine and duodenum	Malignancy	CALIBER
Read	B574z00	Secondary malig neop of small intestine or duodenum NOS	Malignancy	CALIBER
Read	B575000	Secondary malignant neoplasm of colon	Malignancy	CALIBER
Read	B575100	Secondary malignant neoplasm of rectum	Malignancy	CALIBER
Read	B575.00	Secondary malignant neoplasm of large intestine and rectum	Malignancy	CALIBER
Read	B575z00	Secondary malig neop of large intestine or rectum NOS	Malignancy	CALIBER
ICD10	C78.4	Secondary malignant neoplasm of small intestine	Malignancy	CALIBER
ICD10	C78.5	Secondary malignant neoplasm of large intestine and rectum	Malignancy	CALIBER
Read	B583000	Secondary malignant neoplasm of brain	Malignancy	CALIBER
Read	B583100	Secondary malignant neoplasm of spinal cord	Malignancy	CALIBER
Read	B583200	Cerebral metastasis	Malignancy	CALIBER
Read	B583.00	Secondary malignant neoplasm of brain and spinal cord	Malignancy	CALIBER
Read	B583z00	Secondary malignant neoplasm of brain or spinal cord NOS	Malignancy	CALIBER
ICD10	C79.3	Secondary malignant neoplasm of brain and cerebral meninges	Malignancy	CALIBER
ICD10	C79.4	Secondary malignant neoplasm of other and unspecified parts of nervous system	Malignancy	CALIBER
Read	B570.00	Secondary malignant neoplasm of lung	Malignancy	CALIBER
ICD10	C78.0	Secondary malignant neoplasm of lung	Malignancy	CALIBER
Read	B560000	Secondary and unspec malig neop of superficial parotid LN	Malignancy	CALIBER
Read	B560100	Secondary and unspec malignant neoplasm mastoid lymph nodes	Malignancy	CALIBER

Read	B560200	Secondary and unspec malig neop superficial cervical LN	Malignancy	CALIBER
Read	B560300	Secondary and unspec malignant neoplasm occipital lymph node	Malignancy	CALIBER
Read	B560400	Secondary and unspec malig neop deep parotid lymph nodes	Malignancy	CALIBER
Read	B560500	Secondary and unspec malig neop submandibular lymph nodes	Malignancy	CALIBER
Read	B560600	Secondary and unspec malig neop of facial lymph nodes	Malignancy	CALIBER
Read	B560700	Secondary and unspec malig neop submental lymph nodes	Malignancy	CALIBER
Read	B560800	Secondary and unspec malig neop anterior cervical LN	Malignancy	CALIBER
Read	B560900	Secondary and unspec malig neop deep cervical LN	Malignancy	CALIBER
Read	B560.00	Secondary and unspec malig neop lymph nodes head/face/neck	Malignancy	CALIBER
Read	B560z00	Secondary unspec malig neop lymph nodes head/face/neck NOS	Malignancy	CALIBER
Read	B561000	Secondary and unspec malig neop internal mammary lymph nodes	Malignancy	CALIBER
Read	B561100	Secondary and unspec malig neop intercostal lymph nodes	Malignancy	CALIBER
Read	B561200	Secondary and unspec malig neop diaphragmatic lymph nodes	Malignancy	CALIBER
Read	B561300	Secondary and unspec malig neop ant mediastinal lymph nodes	Malignancy	CALIBER
Read	B561400	Secondary and unspec malig neop post mediastinal lymph nodes	Malignancy	CALIBER
Read	B561500	Secondary and unspec malig neop paratracheal lymph nodes	Malignancy	CALIBER
Read	B561600	Secondary and unspec malig neop superfic tracheobronchial LN	Malignancy	CALIBER
Read	B561700	Secondary and unspec malig neop inferior tracheobronchial LN	Malignancy	CALIBER
Read	B561800	Secondary and unspec malig neop bronchopulmonary lymph nodes	Malignancy	CALIBER
Read	B561900	Secondary and unspec malig neop pulmonary lymph nodes	Malignancy	CALIBER
Read	B561.00	Secondary and unspec malig neop intrathoracic lymph nodes	Malignancy	CALIBER
Read	B561z00	Secondary and unspec malig neop intrathoracic LN NOS	Malignancy	CALIBER
Read	B562000	Secondary and unspec malig neop coeliac lymph nodes	Malignancy	CALIBER
Read	B562100	Secondary and unspec malig neop superficial mesenteric LN	Malignancy	CALIBER

Read	B562200	Secondary and unspec malig neop inferior mesenteric LN	Malignancy	CALIBER
Read	B562300	Secondary and unspec malig neop common iliac lymph nodes	Malignancy	CALIBER
Read	B562400	Secondary and unspec malig neop external iliac lymph nodes	Malignancy	CALIBER
Read	B562.00	Secondary and unspec malig neop intra-abdominal lymph nodes	Malignancy	CALIBER
Read	B562z00	Secondary and unspec malig neop intra-abdominal LN NOS	Malignancy	CALIBER
Read	B563000	Secondary and unspec malig neop axillary lymph nodes	Malignancy	CALIBER
Read	B563100	Secondary and unspec malig neop supratrochlear lymph nodes	Malignancy	CALIBER
Read	B563200	Secondary and unspec malig neop infraclavicular lymph nodes	Malignancy	CALIBER
Read	B563300	Secondary and unspec malig neop pectoral lymph nodes	Malignancy	CALIBER
Read	B563.00	Secondary and unspec malig neop axilla and upper limb LN	Malignancy	CALIBER
Read	B563z00	Secondary and unspec malig neop axilla and upper limb LN NOS	Malignancy	CALIBER
Read	B564000	Secondary and unspec malig neop superficial inguinal LN	Malignancy	CALIBER
Read	B564100	Secondary and unspec malig neop deep inguinal lymph nodes	Malignancy	CALIBER
Read	B564.00	Secondary and unspec malig neop inguinal and lower limb LN	Malignancy	CALIBER
Read	B564z00	Secondary and unspec malig neop of inguinal and leg LN NOS	Malignancy	CALIBER
Read	B565000	Secondary and unspec malig neop internal iliac lymph nodes	Malignancy	CALIBER
Read	B565200	Secondary and unspec malig neop circumflex iliac LN	Malignancy	CALIBER
Read	B565300	Secondary and unspec malig neop sacral lymph nodes	Malignancy	CALIBER
Read	B565.00	Secondary and unspec malig neop intrapelvic lymph nodes	Malignancy	CALIBER
Read	B565z00	Secondary and unspec malig neop intrapelvic LN NOS	Malignancy	CALIBER
Read	B56..00	Secondary and unspecified malignant neoplasm of lymph nodes	Malignancy	CALIBER
Read	B56..11	Lymph node metastases	Malignancy	CALIBER
Read	B56y.00	Secondary and unspec malig neop lymph nodes multiple sites	Malignancy	CALIBER
Read	B56z.00	Secondary and unspec malig neop lymph nodes NOS	Malignancy	CALIBER

Read	ByuC200	[X]2ndry+unspcf malignant neoplasm lymph nodes/multi regions	Malignancy	CALIBER
ICD10	C77	Secondary and unspecified malignant neoplasm of lymph nodes	Malignancy	CALIBER
Read	1D18.00	Pain from metastases	Malignancy	CALIBER
Read	5136	X-ray metastasis control	Malignancy	CALIBER
Read	B571.00	Secondary malignant neoplasm of mediastinum	Malignancy	CALIBER
Read	B573.00	Secondary malignant neoplasm of other respiratory organs	Malignancy	CALIBER
Read	B57..00	Secondary malig neop of respiratory and digestive systems	Malignancy	CALIBER
Read	B57..11	Metastases of respiratory and/or digestive systems	Malignancy	CALIBER
Read	B57..12	Secondary carcinoma of respiratory and/or digestive systems	Malignancy	CALIBER
Read	B57y.00	Secondary malignant neoplasm of other digestive organ	Malignancy	CALIBER
Read	B57z.00	Secondary malig neop of respiratory or digestive system NOS	Malignancy	CALIBER
Read	B580.00	Secondary malignant neoplasm of kidney	Malignancy	CALIBER
Read	B581000	Secondary malignant neoplasm of ureter	Malignancy	CALIBER
Read	B581100	Secondary malignant neoplasm of bladder	Malignancy	CALIBER
Read	B581200	Secondary malignant neoplasm of urethra	Malignancy	CALIBER
Read	B581.00	Secondary malignant neoplasm of other urinary organs	Malignancy	CALIBER
Read	B581z00	Secondary malignant neoplasm of other urinary organ NOS	Malignancy	CALIBER
Read	B582000	Secondary malignant neoplasm of skin of head	Malignancy	CALIBER
Read	B582100	Secondary malignant neoplasm of skin of face	Malignancy	CALIBER
Read	B582200	Secondary malignant neoplasm of skin of neck	Malignancy	CALIBER
Read	B582300	Secondary malignant neoplasm of skin of trunk	Malignancy	CALIBER
Read	B582400	Secondary malignant neoplasm of skin of shoulder and arm	Malignancy	CALIBER
Read	B582500	Secondary malignant neoplasm of skin of hip and leg	Malignancy	CALIBER
Read	B582600	Secondary malignant neoplasm of skin of breast	Malignancy	CALIBER
Read	B582.00	Secondary malignant neoplasm of skin	Malignancy	CALIBER
Read	B582z00	Secondary malignant neoplasm of skin NOS	Malignancy	CALIBER
Read	B584.00	Secondary malignant neoplasm of other part of nervous system	Malignancy	CALIBER
Read	B586.00	Secondary malignant neoplasm of ovary	Malignancy	CALIBER

Read	B58..00	Secondary malignant neoplasm of other specified sites	Malignancy	CALIBER
Read	B58..11	Secondary carcinoma of other specified sites	Malignancy	CALIBER
Read	B58y000	Secondary malignant neoplasm of breast	Malignancy	CALIBER
Read	B58y100	Secondary malignant neoplasm of uterus	Malignancy	CALIBER
Read	B58y200	Secondary malignant neoplasm of cervix uteri	Malignancy	CALIBER
Read	B58y211	Secondary cancer of the cervix	Malignancy	CALIBER
Read	B58y300	Secondary malignant neoplasm of vagina	Malignancy	CALIBER
Read	B58y400	Secondary malignant neoplasm of vulva	Malignancy	CALIBER
Read	B58y411	Secondary cancer of the vulva	Malignancy	CALIBER
Read	B58y500	Secondary malignant neoplasm of prostate	Malignancy	CALIBER
Read	B58y600	Secondary malignant neoplasm of testis	Malignancy	CALIBER
Read	B58y700	Secondary malignant neoplasm of penis	Malignancy	CALIBER
Read	B58y800	Secondary malignant neoplasm of epididymis and vas deferens	Malignancy	CALIBER
Read	B58y900	Secondary malignant neoplasm of tongue	Malignancy	CALIBER
Read	B58y.00	Secondary malignant neoplasm of other specified sites	Malignancy	CALIBER
Read	B58yz00	Secondary malignant neoplasm of other specified site NOS	Malignancy	CALIBER
Read	B58z.00	Secondary malignant neoplasm of other specified site NOS	Malignancy	CALIBER
Read	B590.00	Disseminated malignancy NOS	Malignancy	CALIBER
Read	B590.11	Carcinomatosis	Malignancy	CALIBER
Read	B594.00	Secondary malignant neoplasm of unknown site	Malignancy	CALIBER
Read	BB03.00	[M]Neoplasm, metastatic	Malignancy	CALIBER
Read	BB03.11	[M]Secondary neoplasm	Malignancy	CALIBER
Read	BB13.00	[M]Carcinoma, metastatic, NOS	Malignancy	CALIBER
Read	BB13.11	[M]Secondary carcinoma	Malignancy	CALIBER
Read	BB2B.00	[M]Squamous cell carcinoma, metastatic NOS	Malignancy	CALIBER
Read	BB53.00	[M]Adenocarcinoma, metastatic, NOS	Malignancy	CALIBER
Read	BB85100	[M]Metastatic signet ring cell carcinoma	Malignancy	CALIBER
Read	BBy2.00	[M]No microscopic confirmation tumour, clinically metastatic	Malignancy	CALIBER
Read	ByuC300	[X]Secondary malignant neoplasm/oth+unspc respiratory organs	Malignancy	CALIBER
Read	ByuC400	[X]Secondary malignant neoplasm/oth+unspcfd digestive organs	Malignancy	CALIBER
Read	ByuC500	[X]2ndry malignant neoplasm/bladder+oth+unsp urinary organs	Malignancy	CALIBER

Read	ByuC600	[X]2ndry malignant neoplasm/oth+unspec parts/nervous system	Malignancy	CALIBER
Read	ByuC700	[X]Secondary malignant neoplasm of other specified sites	Malignancy	CALIBER
ICD10	C78.1	Secondary malignant neoplasm of mediastinum	Malignancy	CALIBER
ICD10	C78.3	Secondary malignant neoplasm of other and unspecified respiratory organs	Malignancy	CALIBER
ICD10	C78.8	Secondary malignant neoplasm of other and unspecified digestive organs	Malignancy	CALIBER
ICD10	C79.0	Secondary malignant neoplasm of kidney and renal pelvis	Malignancy	CALIBER
ICD10	C79.1	Secondary malignant neoplasm of bladder and other and unspecified urinary organs	Malignancy	CALIBER
ICD10	C79.2	Secondary malignant neoplasm of skin	Malignancy	CALIBER
ICD10	C79.6	Secondary malignant neoplasm of ovary	Malignancy	CALIBER
ICD10	C79.8	Secondary malignant neoplasm of other specified sites	Malignancy	CALIBER
ICD10	C79.9	Secondary malignant neoplasm, unspecified site	Malignancy	CALIBER
Read	B572.00	Secondary malignant neoplasm of pleura	Malignancy	CALIBER
ICD10	C78.2	Secondary malignant neoplasm of pleura	Malignancy	CALIBER
Read	B576000	Secondary malignant neoplasm of retroperitoneum	Malignancy	CALIBER
Read	B576100	Secondary malignant neoplasm of peritoneum	Malignancy	CALIBER
Read	B576200	Malignant ascites	Malignancy	CALIBER
Read	B576.00	Secondary malig neop of retroperitoneum and peritoneum	Malignancy	CALIBER
Read	B576z00	Secondary malig neop of retroperitoneum or peritoneum NOS	Malignancy	CALIBER
ICD10	C78.6	Secondary malignant neoplasm of retroperitoneum and peritoneum	Malignancy	CALIBER
Read	B153.00	Secondary malignant neoplasm of liver	Malignancy	CALIBER
Read	B577.00	Secondary malignant neoplasm of liver	Malignancy	CALIBER
Read	B577.11	Liver metastases	Malignancy	CALIBER
ICD10	C78.7	Secondary malignant neoplasm of liver and intrahepatic bile duct	Malignancy	CALIBER
Read	B330.00	Malignant neoplasm of skin of lip	Malignancy	CALIBER
Read	B331000	Malignant neoplasm of canthus	Malignancy	CALIBER
Read	B331100	Malignant neoplasm of upper eyelid	Malignancy	CALIBER
Read	B331200	Malignant neoplasm of lower eyelid	Malignancy	CALIBER
Read	B331.00	Malignant neoplasm of eyelid including canthus	Malignancy	CALIBER
Read	B332000	Malignant neoplasm of skin of auricle (ear)	Malignancy	CALIBER

Read	B332100	Malignant neoplasm of skin of external auditory meatus	Malignancy	CALIBER
Read	B332200	Malignant neoplasm of pinna NEC	Malignancy	CALIBER
Read	B332.00	Malignant neoplasm skin of ear and external auricular canal	Malignancy	CALIBER
Read	B332z00	Malig neop skin of ear and external auricular canal NOS	Malignancy	CALIBER
Read	B333000	Malignant neoplasm of skin of cheek, external	Malignancy	CALIBER
Read	B333100	Malignant neoplasm of skin of chin	Malignancy	CALIBER
Read	B333200	Malignant neoplasm of skin of eyebrow	Malignancy	CALIBER
Read	B333300	Malignant neoplasm of skin of forehead	Malignancy	CALIBER
Read	B333400	Malignant neoplasm of skin of nose (external)	Malignancy	CALIBER
Read	B333500	Malignant neoplasm of skin of temple	Malignancy	CALIBER
Read	B333.00	Malignant neoplasm skin of other and unspecified parts face	Malignancy	CALIBER
Read	B333z00	Malignant neoplasm skin other and unspec part of face NOS	Malignancy	CALIBER
Read	B334000	Malignant neoplasm of scalp	Malignancy	CALIBER
Read	B334100	Malignant neoplasm of skin of neck	Malignancy	CALIBER
Read	B334.00	Malignant neoplasm of scalp and skin of neck	Malignancy	CALIBER
Read	B334z00	Malignant neoplasm of scalp or skin of neck NOS	Malignancy	CALIBER
Read	B335000	Malignant neoplasm of skin of axillary fold	Malignancy	CALIBER
Read	B335100	Malignant neoplasm of skin of chest, excluding breast	Malignancy	CALIBER
Read	B335200	Malignant neoplasm of skin of breast	Malignancy	CALIBER
Read	B335300	Malignant neoplasm of skin of abdominal wall	Malignancy	CALIBER
Read	B335400	Malignant neoplasm of skin of umbilicus	Malignancy	CALIBER
Read	B335500	Malignant neoplasm of skin of groin	Malignancy	CALIBER
Read	B335600	Malignant neoplasm of skin of perineum	Malignancy	CALIBER
Read	B335700	Malignant neoplasm of skin of back	Malignancy	CALIBER
Read	B335800	Malignant neoplasm of skin of buttock	Malignancy	CALIBER
Read	B335900	Malignant neoplasm of perianal skin	Malignancy	CALIBER
Read	B335A00	Malignant neoplasm of skin of scapular region	Malignancy	CALIBER
Read	B335.00	Malignant neoplasm of skin of trunk, excluding scrotum	Malignancy	CALIBER
Read	B335z00	Malignant neoplasm of skin of trunk, excluding scrotum, NOS	Malignancy	CALIBER
Read	B336000	Malignant neoplasm of skin of shoulder	Malignancy	CALIBER
Read	B336100	Malignant neoplasm of skin of upper arm	Malignancy	CALIBER

Read	B336200	Malignant neoplasm of skin of fore-arm	Malignancy	CALIBER
Read	B336300	Malignant neoplasm of skin of hand	Malignancy	CALIBER
Read	B336400	Malignant neoplasm of skin of finger	Malignancy	CALIBER
Read	B336500	Malignant neoplasm of skin of thumb	Malignancy	CALIBER
Read	B336.00	Malignant neoplasm of skin of upper limb and shoulder	Malignancy	CALIBER
Read	B336z00	Malignant neoplasm of skin of upper limb or shoulder NOS	Malignancy	CALIBER
Read	B337000	Malignant neoplasm of skin of hip	Malignancy	CALIBER
Read	B337100	Malignant neoplasm of skin of thigh	Malignancy	CALIBER
Read	B337200	Malignant neoplasm of skin of knee	Malignancy	CALIBER
Read	B337300	Malignant neoplasm of skin of popliteal fossa area	Malignancy	CALIBER
Read	B337400	Malignant neoplasm of skin of lower leg	Malignancy	CALIBER
Read	B337500	Malignant neoplasm of skin of ankle	Malignancy	CALIBER
Read	B337600	Malignant neoplasm of skin of heel	Malignancy	CALIBER
Read	B337700	Malignant neoplasm of skin of foot	Malignancy	CALIBER
Read	B337800	Malignant neoplasm of skin of toe	Malignancy	CALIBER
Read	B337900	Malignant neoplasm of skin of great toe	Malignancy	CALIBER
Read	B337.00	Malignant neoplasm of skin of lower limb and hip	Malignancy	CALIBER
Read	B337z00	Malignant neoplasm of skin of lower limb or hip NOS	Malignancy	CALIBER
Read	B338.00	Squamous cell carcinoma of skin	Malignancy	CALIBER
Read	B339.00	Dermatofibrosarcoma protuberans	Malignancy	CALIBER
Read	B33..00	Other malignant neoplasm of skin	Malignancy	CALIBER
Read	B33..14	Malignant neoplasm of sebaceous gland	Malignancy	CALIBER
Read	B33..15	Malignant neoplasm of sweat gland	Malignancy	CALIBER
Read	B33X.00	Malignant neoplasm overlapping lesion of skin	Malignancy	CALIBER
Read	B33y.00	Malignant neoplasm of other specified skin sites	Malignancy	CALIBER
Read	B33z.00	Malignant neoplasm of skin NOS	Malignancy	CALIBER
Read	B33z.11	Squamous cell carcinoma of skin NOS	Malignancy	CALIBER
Read	BB2A.13	[M]Squamous cell carcinoma of skin NOS	Malignancy	CALIBER
Read	BB35.00	[M]Basosquamous carcinoma	Malignancy	CALIBER
Read	BB36.00	[M]Metatypical carcinoma	Malignancy	CALIBER
Read	BB60100	[M]Skin appendage carcinoma	Malignancy	CALIBER
Read	BB61200	[M]Sweat gland adenocarcinoma	Malignancy	CALIBER
Read	BB62100	[M]Apocrine adenocarcinoma	Malignancy	CALIBER
Read	BB69100	[M]Sebaceous adenocarcinoma	Malignancy	CALIBER
Read	Byu4200	[X]Oth malignant neoplasm/skin of oth+unspecfd parts of face	Malignancy	CALIBER
Read	Byu4300	[X]Malignant neoplasm of skin, unspecified	Malignancy	CALIBER
Read	Byu5A00	[X]Malignant neoplasm overlapping lesion of skin	Malignancy	CALIBER
ICD10	C44	Other malignant neoplasms of skin	Malignancy	CALIBER

Read	F380100	Juvenile or adult myasthenia gravis	Myasthenia gravis	CALIBER
Read	F380.00	Myasthenia gravis	Myasthenia gravis	CALIBER
Read	F380z00	Myasthenia gravis NOS	Myasthenia gravis	CALIBER
ICD10	G70.0	Myasthenia gravis	Myasthenia gravis	CALIBER
Read	G30X000	Acute ST segment elevation myocardial infarction	Myocardial infarction	CALIBER
Read	G307100	Acute non-ST segment elevation myocardial infarction	Myocardial infarction	CALIBER
Read	323..00	ECG: myocardial infarction	Myocardial infarction	CALIBER
Read	3233	ECG: antero-septal infarct.	Myocardial infarction	CALIBER
Read	3234	ECG:posterior/inferior infarct	Myocardial infarction	CALIBER
Read	3235	ECG: subendocardial infarct	Myocardial infarction	CALIBER
Read	3236	ECG: lateral infarction	Myocardial infarction	CALIBER
Read	323Z.00	ECG: myocardial infarct NOS	Myocardial infarction	CALIBER
Read	889A.00	Diab mellit insulin-glucose infus acute myocardial infarct	Myocardial infarction	CALIBER
Read	G30..00	Acute myocardial infarction	Myocardial infarction	CALIBER
Read	G30..12	Coronary thrombosis	Myocardial infarction	CALIBER
Read	G30..13	Cardiac rupture following myocardial infarction (MI)	Myocardial infarction	CALIBER
Read	G30..15	MI - acute myocardial infarction	Myocardial infarction	CALIBER
Read	G30..16	Thrombosis - coronary	Myocardial infarction	CALIBER
Read	G300.00	Acute anterolateral infarction	Myocardial infarction	CALIBER
Read	G301.00	Other specified anterior myocardial infarction	Myocardial infarction	CALIBER
Read	G301000	Acute anteroapical infarction	Myocardial infarction	CALIBER
Read	G301100	Acute anteroseptal infarction	Myocardial infarction	CALIBER
Read	G301z00	Anterior myocardial infarction NOS	Myocardial infarction	CALIBER
Read	G302.00	Acute inferolateral infarction	Myocardial infarction	CALIBER
Read	G303.00	Acute inferoposterior infarction	Myocardial infarction	CALIBER

Read	G304.00	Posterior myocardial infarction NOS	Myocardial infarction	CALIBER
Read	G305.00	Lateral myocardial infarction NOS	Myocardial infarction	CALIBER
Read	G306.00	True posterior myocardial infarction	Myocardial infarction	CALIBER
Read	G307.00	Acute subendocardial infarction	Myocardial infarction	CALIBER
Read	G307000	Acute non-Q wave infarction	Myocardial infarction	CALIBER
Read	G308.00	Inferior myocardial infarction NOS	Myocardial infarction	CALIBER
Read	G309.00	Acute Q-wave infarct	Myocardial infarction	CALIBER
Read	G30B.00	Acute posterolateral myocardial infarction	Myocardial infarction	CALIBER
Read	G30X.00	Acute transmural myocardial infarction of unspecif site	Myocardial infarction	CALIBER
Read	G30y.00	Other acute myocardial infarction	Myocardial infarction	CALIBER
Read	G30y000	Acute atrial infarction	Myocardial infarction	CALIBER
Read	G30y100	Acute papillary muscle infarction	Myocardial infarction	CALIBER
Read	G30y200	Acute septal infarction	Myocardial infarction	CALIBER
Read	G30yz00	Other acute myocardial infarction NOS	Myocardial infarction	CALIBER
Read	G30z.00	Acute myocardial infarction NOS	Myocardial infarction	CALIBER
Read	G31y100	Microinfarction of heart	Myocardial infarction	CALIBER
Read	G38..00	Postoperative myocardial infarction	Myocardial infarction	CALIBER
Read	G380.00	Postoperative transmural myocardial infarction anterior wall	Myocardial infarction	CALIBER
Read	G381.00	Postoperative transmural myocardial infarction inferior wall	Myocardial infarction	CALIBER
Read	G384.00	Postoperative subendocardial myocardial infarction	Myocardial infarction	CALIBER
Read	G38z.00	Postoperative myocardial infarction, unspecified	Myocardial infarction	CALIBER
Read	Gyu3400	[X]Acute transmural myocardial infarction of unspecif site	Myocardial infarction	CALIBER
ICD10	I21	Acute myocardial infarction	Myocardial infarction	CALIBER
OPCS	K50.2	Percutaneous transluminal coronary thrombolysis using streptokinase	Myocardial infarction	CALIBER

OPCS	K50.3	Percutaneous transluminal injection of therapeutic substance into coronary artery NEC	Myocardial infarction	CALIBER
Read	A544400	Herpes simplex iridocyclitis	Ocular herpes simplex	CALIBER
Read	A544200	Herpes simplex dendritic keratitis	Ocular herpes simplex	CALIBER
Read	A544300	Herpes simplex disciform keratitis	Ocular herpes simplex	CALIBER
Read	A544.00	Ophthalmic herpes simplex	Ocular herpes simplex	Code Browser
Read	A544z00	Ophthalmic herpes simplex NOS	Ocular herpes simplex	Code Browser
Read	A544500	Herpes simplex ophthalmicus	Ocular herpes simplex	Code Browser
ICD10	H19.1	Herpesviral keratitis and keratoconjunctivitis	Ocular herpes simplex	CALIBER
ICD10	B00.5	Herpesviral ocular disease	Ocular herpes simplex	CALIBER
Read	14GB.00	History of osteoporosis	Osteoporosis	CALIBER
Read	58EG.00	Hip DXA scan result osteoporotic	Osteoporosis	CALIBER
Read	58EM.00	Lumbar DXA scan result osteoporotic	Osteoporosis	CALIBER
Read	58EV.00	Femoral neck DEXA scan result osteoporotic	Osteoporosis	CALIBER
Read	66a..00	Osteoporosis monitoring	Osteoporosis	CALIBER
Read	9kj0.00	Bone sparing drug treatment offered for osteoporosis - ESA	Osteoporosis	CALIBER
Read	9kj..00	Osteoporosis - enhanced services administration	Osteoporosis	CALIBER
Read	9Od0.00	Attends osteoporosis monitoring	Osteoporosis	CALIBER
Read	9Od2.00	Osteoporosis monitoring default	Osteoporosis	CALIBER
Read	9Od3.00	Osteoporosis monitoring first letter	Osteoporosis	CALIBER
Read	9Od4.00	Osteoporosis monitoring second letter	Osteoporosis	CALIBER
Read	9Od5.00	Osteoporosis monitoring third letter	Osteoporosis	CALIBER
Read	9Od6.00	Osteoporosis monitoring verbal invitation	Osteoporosis	CALIBER
Read	9Od7.00	Osteoporosis monitoring telephone invitation	Osteoporosis	CALIBER
Read	9Od8.00	Osteoporosis monitoring deleted	Osteoporosis	CALIBER
Read	9Od9.00	Osteoporosis monitoring check done	Osteoporosis	CALIBER
Read	9Od..00	Osteoporosis monitoring administration	Osteoporosis	CALIBER
Read	N330000	Osteoporosis, unspecified	Osteoporosis	CALIBER
Read	N330100	Senile osteoporosis	Osteoporosis	CALIBER
Read	N330200	Postmenopausal osteoporosis	Osteoporosis	CALIBER
Read	N330300	Idiopathic osteoporosis	Osteoporosis	CALIBER
Read	N330400	Dissuse osteoporosis	Osteoporosis	CALIBER
Read	N330500	Drug-induced osteoporosis	Osteoporosis	CALIBER

Read	N330600	Postoophorectomy osteoporosis	Osteoporosis	CALIBER
Read	N330700	Postsurgical malabsorption osteoporosis	Osteoporosis	CALIBER
Read	N330A00	Osteoporosis in endocrine disorders	Osteoporosis	CALIBER
Read	N330B00	Vertebral osteoporosis	Osteoporosis	CALIBER
Read	N330C00	Osteoporosis localized to spine	Osteoporosis	CALIBER
Read	N330D00	Osteoporosis due to corticosteroids	Osteoporosis	CALIBER
Read	N330.00	Osteoporosis	Osteoporosis	CALIBER
Read	N330z00	Osteoporosis NOS	Osteoporosis	CALIBER
Read	N331200	Postoophorectomy osteoporosis with pathological fracture	Osteoporosis	CALIBER
Read	N331300	Osteoporosis of disuse with pathological fracture	Osteoporosis	CALIBER
Read	N331400	Postsurgical malabsorption osteoporosis with path fracture	Osteoporosis	CALIBER
Read	N331500	Drug-induced osteoporosis with pathological fracture	Osteoporosis	CALIBER
Read	N331600	Idiopathic osteoporosis with pathological fracture	Osteoporosis	CALIBER
Read	N331800	Osteoporosis + pathological fracture lumbar vertebrae	Osteoporosis	CALIBER
Read	N331900	Osteoporosis + pathological fracture thoracic vertebrae	Osteoporosis	CALIBER
Read	N331A00	Osteoporosis + pathological fracture cervical vertebrae	Osteoporosis	CALIBER
Read	N331B00	Postmenopausal osteoporosis with pathological fracture	Osteoporosis	CALIBER
Read	N331H00	Collapse of cervical vertebra due to osteoporosis	Osteoporosis	CALIBER
Read	N331J00	Collapse of lumbar vertebra due to osteoporosis	Osteoporosis	CALIBER
Read	N331K00	Collapse of thoracic vertebra due to osteoporosis	Osteoporosis	CALIBER
Read	N331L00	Collapse of vertebra due to osteoporosis NOS	Osteoporosis	CALIBER
Read	N331M00	Fragility fracture due to unspecified osteoporosis	Osteoporosis	CALIBER
Read	N331M11	Minimal trauma fracture due to unspecified osteoporosis	Osteoporosis	CALIBER
Read	N331N00	Fragility fracture	Osteoporosis	CALIBER
Read	N331N11	Minimal trauma fracture	Osteoporosis	CALIBER
Read	N374600	Osteoporotic kyphosis	Osteoporosis	CALIBER
Read	NyuB000	[X]Other osteoporosis with pathological fracture	Osteoporosis	CALIBER
Read	NyuB100	[X]Other osteoporosis	Osteoporosis	CALIBER
Read	NyuB200	[X]Osteoporosis in other disorders classified elsewhere	Osteoporosis	CALIBER

Read	NyuB800	[X]Unspecified osteoporosis with pathological fracture	Osteoporosis	CALIBER
ICD10	M80	Osteoporosis with pathological fracture	Osteoporosis	CALIBER
ICD10	M81	Osteoporosis without pathological fracture	Osteoporosis	CALIBER
ICD10	M82	Osteoporosis in diseases classified elsewhere	Osteoporosis	CALIBER
Read	7612111	Balfour excision of gastric ulcer	Peptic ulcer	CALIBER
Read	7612500	Resection of gastric ulcer by cautery	Peptic ulcer	CALIBER
Read	761D500	Endoscopic injection haemostasis of duodenal ulcer	Peptic ulcer	CALIBER
Read	761D600	Endoscopic injection haemostasis of gastric ulcer	Peptic ulcer	CALIBER
Read	761J000	Closure of perforated gastric ulcer	Peptic ulcer	CALIBER
Read	761J100	Closure of gastric ulcer NEC	Peptic ulcer	CALIBER
Read	761J111	Suture of ulcer of stomach NEC	Peptic ulcer	CALIBER
Read	761J.00	Operations on gastric ulcer	Peptic ulcer	CALIBER
Read	761J.11	Stomach ulcer operations	Peptic ulcer	CALIBER
Read	761Jy00	Other specified operation on gastric ulcer	Peptic ulcer	CALIBER
Read	761Jz00	Operation on gastric ulcer NOS	Peptic ulcer	CALIBER
Read	7627000	Closure of perforated duodenal ulcer	Peptic ulcer	CALIBER
Read	7627100	Suture of duodenal ulcer not elsewhere classified	Peptic ulcer	CALIBER
Read	7627200	Oversew of blood vessel of duodenal ulcer	Peptic ulcer	CALIBER
Read	7627	Operations on duodenal ulcer	Peptic ulcer	CALIBER
Read	7627y00	Other specified operation on duodenal ulcer	Peptic ulcer	CALIBER
Read	7627z00	Operation on duodenal ulcer NOS	Peptic ulcer	CALIBER
Read	J110000	Acute gastric ulcer without mention of complication	Peptic ulcer	CALIBER
Read	J110100	Acute gastric ulcer with haemorrhage	Peptic ulcer	CALIBER
Read	J110111	Bleeding acute gastric ulcer	Peptic ulcer	CALIBER
Read	J110200	Acute gastric ulcer with perforation	Peptic ulcer	CALIBER
Read	J110300	Acute gastric ulcer with haemorrhage and perforation	Peptic ulcer	CALIBER
Read	J110400	Acute gastric ulcer with obstruction	Peptic ulcer	CALIBER
Read	J110.00	Acute gastric ulcer	Peptic ulcer	CALIBER
Read	J110y00	Acute gastric ulcer unspecified	Peptic ulcer	CALIBER
Read	J110z00	Acute gastric ulcer NOS	Peptic ulcer	CALIBER
Read	J111000	Chronic gastric ulcer without mention of complication	Peptic ulcer	CALIBER
Read	J111100	Chronic gastric ulcer with haemorrhage	Peptic ulcer	CALIBER
Read	J111111	Bleeding chronic gastric ulcer	Peptic ulcer	CALIBER
Read	J111200	Chronic gastric ulcer with perforation	Peptic ulcer	CALIBER
Read	J111211	Perforated chronic gastric ulcer	Peptic ulcer	CALIBER

Read	J111300	Chronic gastric ulcer with haemorrhage and perforation	Peptic ulcer	CALIBER
Read	J111400	Chronic gastric ulcer with obstruction	Peptic ulcer	CALIBER
Read	J111.00	Chronic gastric ulcer	Peptic ulcer	CALIBER
Read	J111y00	Chronic gastric ulcer unspecified	Peptic ulcer	CALIBER
Read	J111z00	Chronic gastric ulcer NOS	Peptic ulcer	CALIBER
Read	J112.00	Anti-platelet induced gastric ulcer	Peptic ulcer	CALIBER
Read	J112z00	Anti-platelet induced gastric ulcer NOS	Peptic ulcer	CALIBER
Read	J113.00	Non steroidal anti inflammatory drug induced gastric ulcer	Peptic ulcer	CALIBER
Read	J113z00	Non steroidal anti inflammatory drug induced gastric ulc NOS	Peptic ulcer	CALIBER
Read	J11..00	Gastric ulcer - (GU)	Peptic ulcer	CALIBER
Read	J11..11	Prepyloric ulcer	Peptic ulcer	CALIBER
Read	J11..12	Pyloric ulcer	Peptic ulcer	CALIBER
Read	J11y000	Unspecified gastric ulcer without mention of complication	Peptic ulcer	CALIBER
Read	J11y100	Unspecified gastric ulcer with haemorrhage	Peptic ulcer	CALIBER
Read	J11y200	Unspecified gastric ulcer with perforation	Peptic ulcer	CALIBER
Read	J11y400	Unspecified gastric ulcer with obstruction	Peptic ulcer	CALIBER
Read	J11y.00	Unspecified gastric ulcer	Peptic ulcer	CALIBER
Read	J11yy00	Unspec gastric ulcer; unspec haemorrhage and/or perforation	Peptic ulcer	CALIBER
Read	J11yz00	Unspecified gastric ulcer NOS	Peptic ulcer	CALIBER
Read	J11z.00	Gastric ulcer NOS	Peptic ulcer	CALIBER
Read	J11z.11	Gastric erosions	Peptic ulcer	CALIBER
Read	J11z.12	Multiple gastric ulcers	Peptic ulcer	CALIBER
Read	J120000	Acute duodenal ulcer without mention of complication	Peptic ulcer	CALIBER
Read	J120100	Acute duodenal ulcer with haemorrhage	Peptic ulcer	CALIBER
Read	J120200	Acute duodenal ulcer with perforation	Peptic ulcer	CALIBER
Read	J120300	Acute duodenal ulcer with haemorrhage and perforation	Peptic ulcer	CALIBER
Read	J120400	Acute duodenal ulcer with obstruction	Peptic ulcer	CALIBER
Read	J120.00	Acute duodenal ulcer	Peptic ulcer	CALIBER
Read	J120y00	Acute duodenal ulcer unspecified	Peptic ulcer	CALIBER
Read	J120z00	Acute duodenal ulcer NOS	Peptic ulcer	CALIBER
Read	J121000	Chronic duodenal ulcer without mention of complication	Peptic ulcer	CALIBER
Read	J121100	Chronic duodenal ulcer with haemorrhage	Peptic ulcer	CALIBER
Read	J121111	Bleeding chronic duodenal ulcer	Peptic ulcer	CALIBER
Read	J121200	Chronic duodenal ulcer with perforation	Peptic ulcer	CALIBER
Read	J121211	Perforated chronic duodenal ulcer	Peptic ulcer	CALIBER

Read	J121300	Chronic duodenal ulcer with haemorrhage and perforation	Peptic ulcer	CALIBER
Read	J121400	Chronic duodenal ulcer with obstruction	Peptic ulcer	CALIBER
Read	J121.00	Chronic duodenal ulcer	Peptic ulcer	CALIBER
Read	J121y00	Chronic duodenal ulcer unspecified	Peptic ulcer	CALIBER
Read	J121z00	Chronic duodenal ulcer NOS	Peptic ulcer	CALIBER
Read	J122.00	Duodenal ulcer disease	Peptic ulcer	CALIBER
Read	J123.00	Duodenal erosion	Peptic ulcer	CALIBER
Read	J124.00	Recurrent duodenal ulcer	Peptic ulcer	CALIBER
Read	J125.00	Anti-platelet induced duodenal ulcer	Peptic ulcer	CALIBER
Read	J126.00	Non steroidal anti inflammatory drug induced duodenal ulcer	Peptic ulcer	CALIBER
Read	J12..00	Duodenal ulcer - (DU)	Peptic ulcer	CALIBER
Read	J12y000	Unspecified duodenal ulcer without mention of complication	Peptic ulcer	CALIBER
Read	J12y100	Unspecified duodenal ulcer with haemorrhage	Peptic ulcer	CALIBER
Read	J12y200	Unspecified duodenal ulcer with perforation	Peptic ulcer	CALIBER
Read	J12y300	Unspecified duodenal ulcer with haemorrhage and perforation	Peptic ulcer	CALIBER
Read	J12y400	Unspecified duodenal ulcer with obstruction	Peptic ulcer	CALIBER
Read	J12y.00	Unspecified duodenal ulcer	Peptic ulcer	CALIBER
Read	J12yy00	Unspec duodenal ulcer; unspec haemorrhage and/or perforation	Peptic ulcer	CALIBER
Read	J12yz00	Unspecified duodenal ulcer NOS	Peptic ulcer	CALIBER
Read	J12z.00	Duodenal ulcer NOS	Peptic ulcer	CALIBER
Read	J130000	Acute peptic ulcer without mention of complication	Peptic ulcer	CALIBER
Read	J130100	Acute peptic ulcer with haemorrhage	Peptic ulcer	CALIBER
Read	J130200	Acute peptic ulcer with perforation	Peptic ulcer	CALIBER
Read	J130300	Acute peptic ulcer with haemorrhage and perforation	Peptic ulcer	CALIBER
Read	J130.00	Acute peptic ulcer	Peptic ulcer	CALIBER
Read	J130y00	Acute peptic ulcer unspecified	Peptic ulcer	CALIBER
Read	J130z00	Acute peptic ulcer NOS	Peptic ulcer	CALIBER
Read	J131000	Chronic peptic ulcer without mention of complication	Peptic ulcer	CALIBER
Read	J131100	Chronic peptic ulcer with haemorrhage	Peptic ulcer	CALIBER
Read	J131200	Chronic peptic ulcer with perforation	Peptic ulcer	CALIBER
Read	J131400	Chronic peptic ulcer with obstruction	Peptic ulcer	CALIBER
Read	J131.00	Chronic peptic ulcer	Peptic ulcer	CALIBER
Read	J131y00	Chronic peptic ulcer unspecified	Peptic ulcer	CALIBER
Read	J131z00	Chronic peptic ulcer NOS	Peptic ulcer	CALIBER

Read	J13..00	Peptic ulcer - (PU) site unspecified	Peptic ulcer	CALIBER
Read	J13..11	Stress ulcer NOS	Peptic ulcer	CALIBER
Read	J13y000	Unspecified peptic ulcer without mention of complication	Peptic ulcer	CALIBER
Read	J13y100	Unspecified peptic ulcer with haemorrhage	Peptic ulcer	CALIBER
Read	J13y200	Unspecified peptic ulcer with perforation	Peptic ulcer	CALIBER
Read	J13y300	Unspecified peptic ulcer with haemorrhage and perforation	Peptic ulcer	CALIBER
Read	J13y400	Unspecified peptic ulcer with obstruction	Peptic ulcer	CALIBER
Read	J13y.00	Unspecified peptic ulcer	Peptic ulcer	CALIBER
Read	J13yz00	Unspecified peptic ulcer NOS	Peptic ulcer	CALIBER
Read	J13z.00	Peptic ulcer NOS	Peptic ulcer	CALIBER
Read	J140100	Acute gastrojejunal ulcer with haemorrhage	Peptic ulcer	CALIBER
Read	J140200	Acute gastrojejunal ulcer with perforation	Peptic ulcer	CALIBER
Read	J140300	Acute gastrojejunal ulcer with haemorrhage and perforation	Peptic ulcer	CALIBER
Read	J140.00	Acute gastrojejunal ulcer	Peptic ulcer	CALIBER
Read	J140z00	Acute gastrojejunal ulcer NOS	Peptic ulcer	CALIBER
Read	J141300	Chronic gastrojejunal ulcer with haemorrhage and perforation	Peptic ulcer	CALIBER
Read	J141.00	Chronic gastrojejunal ulcer	Peptic ulcer	CALIBER
Read	J14..00	Gastrojejunal ulcer (GJU)	Peptic ulcer	CALIBER
Read	J14..11	Anastomotic ulcer	Peptic ulcer	CALIBER
Read	J14..12	Gastrocolic ulcer	Peptic ulcer	CALIBER
Read	J14..13	Jejunal ulcer	Peptic ulcer	CALIBER
Read	J14y100	Unspecified gastrojejunal ulcer with haemorrhage	Peptic ulcer	CALIBER
Read	J14y200	Unspecified gastrojejunal ulcer with perforation	Peptic ulcer	CALIBER
Read	J14y.00	Unspecified gastrojejunal ulcer	Peptic ulcer	CALIBER
Read	J14yz00	Unspecified gastrojejunal ulcer NOS	Peptic ulcer	CALIBER
Read	J14z.00	Gastrojejunal ulcer NOS	Peptic ulcer	CALIBER
Read	J17y800	Healed gastric ulcer leaving a scar	Peptic ulcer	CALIBER
ICD10	K25	Gastric ulcer	Peptic ulcer	CALIBER
ICD10	K26	Duodenal ulcer	Peptic ulcer	CALIBER
ICD10	K27	Peptic ulcer, site unspecified	Peptic ulcer	CALIBER
ICD10	K28	Gastrojejunal ulcer	Peptic ulcer	CALIBER
OPCS	G35	Operations on ulcer of stomach	Peptic ulcer	CALIBER
OPCS	G35.1	Closure of perforated ulcer of stomach	Peptic ulcer	CALIBER
OPCS	G35.2	Closure of ulcer of stomach NEC	Peptic ulcer	CALIBER
OPCS	G35.8	Other specified operations on ulcer of stomach	Peptic ulcer	CALIBER
OPCS	G35.9	Unspecified operations on ulcer of stomach	Peptic ulcer	CALIBER

OPCS	G52	Operations on ulcer of duodenum	Peptic ulcer	CALIBER
OPCS	G52.1	Closure of perforated ulcer of duodenum	Peptic ulcer	CALIBER
OPCS	G52.2	Suture of ulcer of duodenum NEC	Peptic ulcer	CALIBER
OPCS	G52.3	Oversew of blood vessel of duodenal ulcer	Peptic ulcer	CALIBER
OPCS	G52.8	Other specified operations on ulcer of duodenum	Peptic ulcer	CALIBER
OPCS	G52.9	Unspecified operations on ulcer of duodenum	Peptic ulcer	CALIBER
ICD10	A02.1	Salmonella sepsis	Systemic infection	CALIBER
ICD10	A20.7	Septicaemic plague	Systemic infection	CALIBER
ICD10	A22.7	Anthrax sepsis	Systemic infection	CALIBER
ICD10	A26.7	Erysipelothrix sepsis	Systemic infection	CALIBER
ICD10	A32.7	Listerial sepsis	Systemic infection	CALIBER
ICD10	A39.1	Waterhouse-Friderichsen syndrome	Systemic infection	CALIBER
ICD10	A39.2	Acute meningococcaemia	Systemic infection	CALIBER
ICD10	A39.3	Chronic meningococcaemia	Systemic infection	CALIBER
ICD10	A39.4	Meningococcaemia, unspecified	Systemic infection	CALIBER
ICD10	A40	Streptococcal sepsis	Systemic infection	CALIBER
ICD10	A41	Other sepsis	Systemic infection	CALIBER
ICD10	A42.7	Actinomycotic sepsis	Systemic infection	CALIBER
ICD10	B37.7	Candidal sepsis	Systemic infection	CALIBER
ICD10	P36	Bacterial sepsis of newborn	Systemic infection	CALIBER
Read	Qyu4800	[X]Sepsis of newborn due to other+unspecified streptococci	Systemic infection	Code Browser
Read	Qyu4200	[X]Other bacterial sepsis of newborn	Systemic infection	Code Browser
Read	Qyu4100	[X]Sepsis/newborn due to other+unspecified staphylococcus	Systemic infection	Code Browser
Read	Q40y011	Congenital sepsis NOS	Systemic infection	Code Browser
Read	Q40y000	Intrauterine fetal sepsis, unspecified	Systemic infection	Code Browser
Read	Q40W.00	Sepsis of newborn due to other+unspecified streptococci	Systemic infection	Code Browser

Read	Q40A200	Sepsis of newborn due to anaerobes	Systemic infection	Code Browser
Read	Q40A100	Sepsis of newborn due to Escherichia coli	Systemic infection	Code Browser
Read	Q40A000	Sepsis of newborn due to Staphylococcus aureus	Systemic infection	Code Browser
Read	Q40A.00	Sepsis of the newborn	Systemic infection	Code Browser
Read	Q404z00	Umbilical sepsis NOS	Systemic infection	Code Browser
Read	Q404y00	Other specified umbilical sepsis	Systemic infection	Code Browser
Read	L40..11	Sepsis - puerperal	Systemic infection	Code Browser
Read	L090y00	Sepsis NOS following abortion/ectopic/molar pregnancy	Systemic infection	Code Browser
Read	L040011	Spontaneous abortion with sepsis	Systemic infection	Code Browser
Read	K190600	Urosepsis	Systemic infection	Code Browser
Read	J666.00	Biliary sepsis	Systemic infection	Code Browser
Read	H5y0100	Tracheostomy sepsis	Systemic infection	Code Browser
Read	AB2y511	Sepsis due to Candida	Systemic infection	Code Browser
Read	AB2y500	Candidal sepsis	Systemic infection	Code Browser
Read	A3Cz.00	Sepsis NOS	Systemic infection	Code Browser
Read	A3Cy.00	Other specified sepsis	Systemic infection	Code Browser
Read	A3C3y00	Sepsis due to other Gram negative organisms	Systemic infection	Code Browser
Read	A3C3000	Sepsis due to Haemophilus influenzae	Systemic infection	Code Browser
Read	A3C3.11	Sepsis due to Gram negative organisms	Systemic infection	Code Browser
Read	A3C3.00	Sepsis due to Gram negative bacteria	Systemic infection	Code Browser
Read	A3C2.11	Sepsis due to anaerobes	Systemic infection	Code Browser
Read	A3C2.00	Sepsis due to anaerobic bacteria	Systemic infection	Code Browser
Read	A3C1z00	Sepsis due to staphylococcus NOS	Systemic infection	Code Browser
Read	A3C1y00	Sepsis due to other specified staphylococcus	Systemic infection	Code Browser

Read	A3C1000	Sepsis due to Staphylococcus aureus	Systemic infection	Code Browser
Read	A3C1.00	Sepsis due to Staphylococcus	Systemic infection	Code Browser
Read	A3C0z00	Streptococcal sepsis, unspecified	Systemic infection	Code Browser
Read	A3C0y00	Other streptococcal sepsis	Systemic infection	Code Browser
Read	A3C0300	Sepsis due to Streptococcus pneumoniae	Systemic infection	Code Browser
Read	A3C0100	Sepsis due to Streptococcus group B	Systemic infection	Code Browser
Read	A3C0000	Sepsis due to Streptococcus group A	Systemic infection	Code Browser
Read	A3C0.11	Streptococcal sepsis	Systemic infection	Code Browser
Read	A3C0.00	Sepsis due to Streptococcus	Systemic infection	Code Browser
Read	A3C..00	Sepsis	Systemic infection	Code Browser
Read	A396.11	Actinomycotic sepsis	Systemic infection	Code Browser
Read	A396.00	Sepsis due to Actinomyces	Systemic infection	Code Browser
Read	A38z.11	Sepsis	Systemic infection	Code Browser
Read	A270611	Listerial sepsis	Systemic infection	Code Browser
Read	A270600	Sepsis due to Listeria monocytogenes	Systemic infection	Code Browser
Read	A224.00	Sepsis due to Bacillus anthracis	Systemic infection	Code Browser
Read	A023.00	Salmonella sepsis	Systemic infection	Code Browser
Read	A380300	Septicaemia due to streptococcus pneumoniae	Systemic infection	Code Browser
Read	A38y.00	Other specified septicaemias	Systemic infection	Code Browser
Read	A366.00	Meningococcal meningitis with meningococcal septicaemia	Systemic infection	Code Browser
Read	Q407511	Neonatal monilial septicaemia	Systemic infection	Code Browser
Read	Ayu3E00	[X]Other streptococcal septicaemia	Systemic infection	Code Browser
Read	A384100	Haemophilus influenzae septicaemia	Systemic infection	Code Browser
Read	A98yz12	Gonococcal septicaemia	Systemic infection	Code Browser

Read	A202.00	Septicaemic plague	Systemic infection	Code Browser
Read	L293.00	Septicaemia during labour	Systemic infection	Code Browser
Read	A380400	Septicaemia due to enterococcus	Systemic infection	Code Browser
Read	L090z00	Septicaemia NOS following abortive pregnancy	Systemic infection	Code Browser
Read	A384211	E.coli septicaemia	Systemic infection	Code Browser
Read	A270100	Listeria septicaemia	Systemic infection	Code Browser
Read	A380500	Vancomycin resistant enterococcal septicaemia	Systemic infection	Code Browser
Read	A271100	Erysipelothrix septicaemia	Systemic infection	Code Browser
Read	A380000	Septicaemia due to streptococcus, group A	Systemic infection	Code Browser
Read	A383.00	Septicaemia due to anaerobes	Systemic infection	Code Browser
Read	A384400	Serratia septicaemia	Systemic infection	Code Browser
Read	Q40y200	Septicaemia of newborn	Systemic infection	Code Browser
Read	L403000	Puerperal septicaemia unspecified	Systemic infection	Code Browser
Read	L403100	Puerperal septicaemia - delivered with postnatal comp	Systemic infection	Code Browser
Read	A381.00	Staphylococcal septicaemia	Systemic infection	Code Browser
Read	R055511	[D]Septicaemic shock	Systemic infection	Code Browser
Read	SP25400	Postoperative septicaemia	Systemic infection	Code Browser
Read	A384200	Escherichia coli septicaemia	Systemic infection	Code Browser
Read	A380100	Septicaemia due to streptococcus, group B	Systemic infection	Code Browser
Read	Q40y012	Congenital septicaemia	Systemic infection	Code Browser
Read	L293100	Septicaemia during labour - delivered	Systemic infection	Code Browser
Read	L403z00	Puerperal septicaemia NOS	Systemic infection	Code Browser
Read	A382.00	Pneumococcal septicaemia	Systemic infection	Code Browser
Read	A380.00	Streptococcal septicaemia	Systemic infection	Code Browser

Read	A384.00	Septicaemia due to other gram negative organisms	Systemic infection	Code Browser
Read	A381100	Septicaemia due to coagulase-negative staphylococcus	Systemic infection	Code Browser
Read	Ayu3J00	[X]Septicaemia, unspecified	Systemic infection	Code Browser
Read	A384300	Pseudomonas septicaemia	Systemic infection	Code Browser
Read	A362.00	Meningococcal septicaemia	Systemic infection	Code Browser
Read	L403.00	Puerperal septicaemia	Systemic infection	Code Browser
Read	Q407500	Neonatal candida septicaemia	Systemic infection	Code Browser
Read	A384z00	Other gram negative septicaemia NOS	Systemic infection	Code Browser
Read	A021.00	Salmonella septicaemia	Systemic infection	Code Browser
Read	Ayu3G00	[X]Septicaemia due to other gram-negative organisms	Systemic infection	Code Browser
Read	A545.00	Herpes simplex septicaemia	Systemic infection	Code Browser
Read	AB2y300	Candidal septicaemia	Systemic infection	Code Browser
Read	A381000	Septicaemia due to Staphylococcus aureus	Systemic infection	Code Browser
Read	Ayu3F00	[X]Streptococcal septicaemia, unspecified	Systemic infection	Code Browser
Read	Ayu3H00	[X]Other specified septicaemia	Systemic infection	Code Browser
Read	A384000	Gram negative septicaemia NOS	Systemic infection	Code Browser
Read	A38..00	Septicaemia	Systemic infection	Code Browser
Read	A38z.00	Septicaemia NOS	Systemic infection	Code Browser
Read	F396600	Myopathy due to scleroderma	Systemic sclerosis	CALIBER
Read	H572.00	Lung disease with systemic sclerosis	Systemic sclerosis	CALIBER
Read	K0H..00	Acute scleroderma renal crisis	Systemic sclerosis	CALIBER
Read	K0J0.00	Renal involvement in scleroderma	Systemic sclerosis	CALIBER
Read	N001000	Progressive systemic sclerosis	Systemic sclerosis	CALIBER
Read	N001100	CREST syndrome	Systemic sclerosis	CALIBER

Read	N001200	Systemic sclerosis induced by drugs and chemicals	Systemic sclerosis	CALIBER
Read	N001.00	Scleroderma	Systemic sclerosis	CALIBER
Read	N001.11	Acrosclerosis	Systemic sclerosis	CALIBER
Read	N001.12	Systemic sclerosis	Systemic sclerosis	CALIBER
Read	Nyu4500	[X]Other forms of systemic sclerosis	Systemic sclerosis	CALIBER
ICD10	M34	Systemic sclerosis	Systemic sclerosis	CALIBER
Read	14A8100	H/O: Deep Vein Thrombosis	Thromboemboli c disorders	CALIBER
Read	8CMWA0 0	On deep vein thrombosis care pathway	Thromboemboli c disorders	CALIBER
Read	9kg0.00	DVT stage 1 service level - enhanced services administration	Thromboemboli c disorders	CALIBER
Read	9kg..00	Deep vein thrombosis - enhanced services administration	Thromboemboli c disorders	CALIBER
Read	9kg0.11	DVT enhanced services administration stage 1 service level	Thromboemboli c disorders	CALIBER
Read	9kg1.00	DVT stage 2 service level - enhanced services administration	Thromboemboli c disorders	CALIBER
Read	9kg2.00	DVT stage 3 service level - enhanced services administration	Thromboemboli c disorders	CALIBER
Read	F050000	Embolism cavernous sinus	Thromboemboli c disorders	CALIBER
Read	F050.00	Embolism of central nervous system venous sinus	Thromboemboli c disorders	CALIBER
Read	F05..00	Phlebitis and thrombophlebitis of intracranial sinuses	Thromboemboli c disorders	CALIBER
Read	F050100	Embolism superior longitudinal sinus	Thromboemboli c disorders	CALIBER
Read	F050300	Embolism transverse sinus	Thromboemboli c disorders	CALIBER
Read	F051000	Thrombosis cavernous sinus	Thromboemboli c disorders	CALIBER
Read	F051.00	Thrombosis of central nervous system venous sinuses	Thromboemboli c disorders	CALIBER
Read	F051100	Thrombosis of superior longitudinal sinus	Thromboemboli c disorders	CALIBER
Read	F051200	Thrombosis lateral sinus	Thromboemboli c disorders	CALIBER
Read	F051300	Thrombosis transverse sinus	Thromboemboli c disorders	CALIBER
Read	F051z00	Thrombosis of central nervous system venous sinus NOS	Thromboemboli c disorders	CALIBER

Read	F053000	Thrombophlebitis of cavernous sinus	Thromboemboli c disorders	CALIBER
Read	F053.00	Thrombophlebitis of central nervous system venous sinuses	Thromboemboli c disorders	CALIBER
Read	F053100	Thrombophlebitis of superior longitudinal venous sinus	Thromboemboli c disorders	CALIBER
Read	F05z.00	Phlebitis or thrombophlebitis of CNS venous sinus NOS	Thromboemboli c disorders	CALIBER
Read	G676000	Cereb infarct due cerebral venous thrombosis, nonpyogenic	Thromboemboli c disorders	CALIBER
Read	G676.00	Nonpyogenic venous sinus thrombosis	Thromboemboli c disorders	CALIBER
Read	G67A.00	Cerebral vein thrombosis	Thromboemboli c disorders	CALIBER
Read	G801.00	Deep vein phlebitis and thrombophlebitis of the leg	Thromboemboli c disorders	CALIBER
Read	G801.11	Deep vein thrombosis	Thromboemboli c disorders	CALIBER
Read	G801.12	Deep vein thrombosis, leg	Thromboemboli c disorders	CALIBER
Read	G801.13	DVT - Deep vein thrombosis	Thromboemboli c disorders	CALIBER
Read	G801600	Thrombophlebitis of the femoral vein	Thromboemboli c disorders	CALIBER
Read	G801700	Thrombophlebitis of the popliteal vein	Thromboemboli c disorders	CALIBER
Read	G801800	Thrombophlebitis of the anterior tibial vein	Thromboemboli c disorders	CALIBER
Read	G801900	Thrombophlebitis of the dorsalis pedis vein	Thromboemboli c disorders	CALIBER
Read	G801A00	Thrombophlebitis of the posterior tibial vein	Thromboemboli c disorders	CALIBER
Read	G801B00	Deep vein thrombophlebitis of the leg unspecified	Thromboemboli c disorders	CALIBER
Read	G801C00	Deep vein thrombosis of leg related to air travel	Thromboemboli c disorders	CALIBER
Read	G801D00	Deep vein thrombosis of lower limb	Thromboemboli c disorders	CALIBER
Read	G801E00	Deep vein thrombosis of leg related to intravenous drug use	Thromboemboli c disorders	CALIBER
Read	G801F00	Deep vein thrombosis of peroneal vein	Thromboemboli c disorders	CALIBER
Read	G801G00	Recurrent deep vein thrombosis	Thromboemboli c disorders	CALIBER
Read	G801z00	Deep vein phlebitis and thrombophlebitis of the leg NOS	Thromboemboli c disorders	CALIBER
Read	G80y.11	Phlebitis and/or thrombophlebitis of iliac vein	Thromboemboli c disorders	CALIBER

Read	G80y400	Thrombophlebitis of the common iliac vein	Thromboembolic disorders	CALIBER
Read	G80y500	Thrombophlebitis of the internal iliac vein	Thromboembolic disorders	CALIBER
Read	G80y600	Thrombophlebitis of the external iliac vein	Thromboembolic disorders	CALIBER
Read	G80y700	Thrombophlebitis of the iliac vein unspecified	Thromboembolic disorders	CALIBER
Read	G80y800	Phlebitis and thrombophlebitis of the iliac vein NOS	Thromboembolic disorders	CALIBER
Read	G81..00	Portal vein thrombosis	Thromboembolic disorders	CALIBER
Read	G820.00	Budd - Chiari syndrome (hepatic vein thrombosis)	Thromboembolic disorders	CALIBER
Read	G820.11	Hepatic vein thrombosis	Thromboembolic disorders	CALIBER
Read	G822000	Thrombosis of inferior vena cava	Thromboembolic disorders	CALIBER
Read	G822.00	Embolism and thrombosis of the vena cava	Thromboembolic disorders	CALIBER
Read	G823.00	Embolism and thrombosis of the renal vein	Thromboembolic disorders	CALIBER
Read	G824.00	Axillary vein thrombosis	Thromboembolic disorders	CALIBER
Read	G825.00	Thrombosis of subclavian vein	Thromboembolic disorders	CALIBER
Read	G826.00	Thrombosis of internal jugular vein	Thromboembolic disorders	CALIBER
Read	G827.00	Thrombosis of external jugular vein	Thromboembolic disorders	CALIBER
Read	Gyu8000	[X]Phlebitis+thrombophlebitis/oth deep vessls/low extremites	Thromboembolic disorders	CALIBER
Read	J420200	Thrombus of the superior mesenteric veins	Thromboembolic disorders	CALIBER
Read	SP12200	Post operative deep vein thrombosis	Thromboembolic disorders	CALIBER
Read	ZV12800	[V] Personal history deep vein thrombosis	Thromboembolic disorders	CALIBER
Read	ZV12811	[V] Personal history DVT- deep vein thrombosis	Thromboembolic disorders	CALIBER
ICD10	I80.1	Phlebitis and thrombophlebitis of femoral vein	Thromboembolic disorders	CALIBER
ICD10	I80.2	Phlebitis and thrombophlebitis of other deep vessels of lower extremities	Thromboembolic disorders	CALIBER
ICD10	I63.6	Cerebral infarction due to cerebral venous thrombosis, nonpyogenic	Thromboembolic disorders	CALIBER
ICD10	I67.6	Nonpyogenic thrombosis of intracranial venous system	Thromboembolic disorders	CALIBER

ICD10	I81	Portal vein thrombosis	Thromboemboli c disorders	CALIBER
ICD10	I82.0	Budd-Chiari syndrome	Thromboemboli c disorders	CALIBER
ICD10	I82.2	Embolism and thrombosis of vena cava	Thromboemboli c disorders	CALIBER
ICD10	I82.3	Embolism and thrombosis of renal vein	Thromboemboli c disorders	CALIBER
Read	14AC.00	H/O: pulmonary embolus	Thromboemboli c disorders	CALIBER
Read	7A09300	Open embolectomy of pulmonary artery	Thromboemboli c disorders	CALIBER
Read	7A09311	Trendelenburg pulmonary embolectomy	Thromboemboli c disorders	CALIBER
Read	7A0A100	Percutaneous transluminal embolectomy of pulmonary artery	Thromboemboli c disorders	CALIBER
Read	7A0B000	Pulmonary thromboendarterectomy	Thromboemboli c disorders	CALIBER
Read	G401000	Post operative pulmonary embolus	Thromboemboli c disorders	CALIBER
Read	G401100	Recurrent pulmonary embolism	Thromboemboli c disorders	CALIBER
Read	G401.00	Pulmonary embolism	Thromboemboli c disorders	CALIBER
Read	G401.12	Pulmonary embolus	Thromboemboli c disorders	CALIBER
Read	ZV12900	[V] Personal history of pulmonary embolism	Thromboemboli c disorders	CALIBER
ICD10	I26	Pulmonary embolism	Thromboemboli c disorders	CALIBER
Read	1411	H/O: tuberculosis	Tuberculosis	CALIBER
Read	14P9.00	History of tuberculosis drug therapy	Tuberculosis	CALIBER
Read	65V9.00	Notification of tuberculosis	Tuberculosis	CALIBER
Read	65V9.11	TB - tuberculosis notification	Tuberculosis	CALIBER
Read	65Y1.00	On tuberculosis chemoprophylaxis	Tuberculosis	CALIBER
Read	65Y2.00	Streptomycin resistant tuberculosis	Tuberculosis	CALIBER
Read	65Y5.00	Isoniazid resistant tuberculosis	Tuberculosis	CALIBER
Read	65Y8.00	Ciprofloxacin resistant tuberculosis	Tuberculosis	CALIBER
Read	65Y9.00	Inactive tuberculosis	Tuberculosis	CALIBER
Read	65Y9.11	Latent tuberculosis	Tuberculosis	CALIBER
Read	8BAD100	TB chemotherapy	Tuberculosis	CALIBER
Read	A100.00	Primary tuberculous complex	Tuberculosis	CALIBER
Read	A101.00	Tuberculous pleurisy in primary progressive tuberculosis	Tuberculosis	CALIBER
Read	A10..00	Primary tuberculous infection	Tuberculosis	CALIBER
Read	A10y.00	Other primary progressive tuberculosis	Tuberculosis	CALIBER

Read	A10z.00	Primary tuberculous infection NOS	Tuberculosis	CALIBER
Read	A110.00	Infiltrative lung tuberculosis	Tuberculosis	CALIBER
Read	A111.00	Nodular lung tuberculosis	Tuberculosis	CALIBER
Read	A112.00	Tuberculosis of lung with cavitation	Tuberculosis	CALIBER
Read	A113.00	Tuberculosis of bronchus	Tuberculosis	CALIBER
Read	A114.00	Tuberculous fibrosis of lung	Tuberculosis	CALIBER
Read	A115.00	Tuberculous bronchiectasis	Tuberculosis	CALIBER
Read	A116.00	Tuberculous pneumonia	Tuberculosis	CALIBER
Read	A117.00	Tuberculous pneumothorax	Tuberculosis	CALIBER
Read	A11..00	Pulmonary tuberculosis	Tuberculosis	CALIBER
Read	A11..11	Lung tuberculosis	Tuberculosis	CALIBER
Read	A11y.00	Other specified pulmonary tuberculosis	Tuberculosis	CALIBER
Read	A11z.00	Pulmonary tuberculosis NOS	Tuberculosis	CALIBER
Read	A120000	Tuberculosis of pleura	Tuberculosis	CALIBER
Read	A120100	Tuberculous empyema	Tuberculosis	CALIBER
Read	A120200	Tuberculous hydrothorax	Tuberculosis	CALIBER
Read	A120.00	Tuberculous pleurisy	Tuberculosis	CALIBER
Read	A120z00	Tuberculous pleurisy NOS	Tuberculosis	CALIBER
Read	A121000	Tuberculosis of hilar lymph nodes	Tuberculosis	CALIBER
Read	A121100	Tuberculosis of mediastinal lymph nodes	Tuberculosis	CALIBER
Read	A121200	Tuberculosis of tracheobronchial lymph nodes	Tuberculosis	CALIBER
Read	A121.00	Tuberculosis of intrathoracic lymph nodes	Tuberculosis	CALIBER
Read	A121z00	Tuberculosis of intrathoracic lymph nodes NOS	Tuberculosis	CALIBER
Read	A122000	Isolated tracheal tuberculosis	Tuberculosis	CALIBER
Read	A122100	Isolated bronchial tuberculosis	Tuberculosis	CALIBER
Read	A122.00	Isolated tracheal or bronchial tuberculosis	Tuberculosis	CALIBER
Read	A122z00	Isolated tracheal or bronchial tuberculosis NOS	Tuberculosis	CALIBER
Read	A123.00	Tuberculous laryngitis	Tuberculosis	CALIBER
Read	A124000	TB lung confirm sputum microscopy with or without culture	Tuberculosis	CALIBER
Read	A124100	Tuberculosis of lung, confirmed by culture only	Tuberculosis	CALIBER
Read	A124200	Tuberculosis of lung, confirmed histologically	Tuberculosis	CALIBER
Read	A124300	Tuberculosis of lung, confirmed by unspecified means	Tuberculosis	CALIBER
Read	A124400	TB intrathoracic lymph nodes confirm bact histologically	Tuberculosis	CALIBER
Read	A124500	Tuberculosis of larynx, trachea & bronchus conf bact/hist'y	Tuberculosis	CALIBER
Read	A124600	Tuberculous pleurisy, conf bacteriologically/histologically	Tuberculosis	CALIBER
Read	A124700	Primary respiratory TB confirm bact and histologically	Tuberculosis	CALIBER

Read	A124.00	Resp TB bacteriologically and histologically confirmed	Tuberculosis	CALIBER
Read	A125000	Tuberculosis of lung, bacteriologically & histolog'y neg	Tuberculosis	CALIBER
Read	A125100	Tuberculosis lung bact and histological examin not done	Tuberculosis	CALIBER
Read	A125200	Prim respiratory TB without mention of bact or hist confirm	Tuberculosis	CALIBER
Read	A125.00	Respiratory TB not confirmed bact or histologically	Tuberculosis	CALIBER
Read	A125X00	Resp TB unspcf,w'out mention/bacterial or histol confrmtn	Tuberculosis	CALIBER
Read	A12..00	Other respiratory tuberculosis	Tuberculosis	CALIBER
Read	A12y000	Tuberculosis of mediastinum	Tuberculosis	CALIBER
Read	A12y100	Tuberculosis of nasopharynx	Tuberculosis	CALIBER
Read	A12y200	Tuberculosis of nasal septum	Tuberculosis	CALIBER
Read	A12y300	Tuberculosis of nasal sinus	Tuberculosis	CALIBER
Read	A12y.00	Other specified respiratory tuberculosis	Tuberculosis	CALIBER
Read	A12yz00	Other specified respiratory tuberculosis NOS	Tuberculosis	CALIBER
Read	A130000	Tuberculosis of cerebral meninges	Tuberculosis	CALIBER
Read	A130100	Tuberculosis of spinal meninges	Tuberculosis	CALIBER
Read	A130300	Tuberculous meningoencephalitis	Tuberculosis	CALIBER
Read	A130.00	Tuberculous meningitis	Tuberculosis	CALIBER
Read	A130z00	Tuberculous meningitis NOS	Tuberculosis	CALIBER
Read	A131.00	Tuberculoma of meninges	Tuberculosis	CALIBER
Read	A132.00	Tuberculoma of brain	Tuberculosis	CALIBER
Read	A133.00	Tuberculous abscess of brain	Tuberculosis	CALIBER
Read	A134.00	Tuberculoma of spinal cord	Tuberculosis	CALIBER
Read	A135.00	Tuberculous abscess of spinal cord	Tuberculosis	CALIBER
Read	A136000	Tuberculous encephalitis	Tuberculosis	CALIBER
Read	A136100	Tuberculous myelitis	Tuberculosis	CALIBER
Read	A136.00	Tuberculous encephalitis or myelitis	Tuberculosis	CALIBER
Read	A136z00	Tuberculous encephalitis or myelitis NOS	Tuberculosis	CALIBER
Read	A13..00	Tuberculosis of meninges and central nervous system	Tuberculosis	CALIBER
Read	A13y.00	Other specified tuberculosis of central nervous system	Tuberculosis	CALIBER
Read	A13z.00	Tuberculosis of central nervous system NOS	Tuberculosis	CALIBER
Read	A140.00	Tuberculous peritonitis	Tuberculosis	CALIBER
Read	A14..00	Tuberculosis of intestines, peritoneum and mesenteric glands	Tuberculosis	CALIBER
Read	A14y100	Tuberculosis of large intestine	Tuberculosis	CALIBER
Read	A14y200	Tuberculosis of small intestine	Tuberculosis	CALIBER
Read	A14y300	Tuberculosis of mesenteric lymph glands	Tuberculosis	CALIBER
Read	A14y400	Tuberculosis of rectum	Tuberculosis	CALIBER
Read	A14y500	Tuberculosis of retroperitoneal lymph nodes	Tuberculosis	CALIBER
Read	A14y.00	Other gastrointestinal tract tuberculosis	Tuberculosis	CALIBER

Read	A14yz00	Other gastrointestinal tract tuberculosis NOS	Tuberculosis	CALIBER
Read	A14z.00	Tuberculosis of gastrointestinal tract NOS	Tuberculosis	CALIBER
Read	A150.00	Tuberculosis of vertebral column - Pott's	Tuberculosis	CALIBER
Read	A151.00	Tuberculosis of hip	Tuberculosis	CALIBER
Read	A152.00	Tuberculosis of knee	Tuberculosis	CALIBER
Read	A153.00	Tuberculosis limb bones - Tuberculous dactylitis	Tuberculosis	CALIBER
Read	A154.00	Tuberculous mastoiditis	Tuberculosis	CALIBER
Read	A15..00	Tuberculosis of bones and joints	Tuberculosis	CALIBER
Read	A15..11	Tuberculous osteomylelytis	Tuberculosis	CALIBER
Read	A15..12	Tuberculous arthritis	Tuberculosis	CALIBER
Read	A15..13	Tuberculous synovitis	Tuberculosis	CALIBER
Read	A15x.00	Tuberculosis of other specified bones	Tuberculosis	CALIBER
Read	A15y.00	Tuberculosis of other specified joint	Tuberculosis	CALIBER
Read	A15z.00	Tuberculosis of bones or joints NOS	Tuberculosis	CALIBER
Read	A160000	Tuberculous nephropathy	Tuberculosis	CALIBER
Read	A160100	Tuberculous pyelitis	Tuberculosis	CALIBER
Read	A160200	Tuberculous pyelonephritis	Tuberculosis	CALIBER
Read	A160.00	Tuberculosis of kidney	Tuberculosis	CALIBER
Read	A160.11	Renal tuberculosis	Tuberculosis	CALIBER
Read	A160z00	Tuberculosis of kidney NOS	Tuberculosis	CALIBER
Read	A161.00	Tuberculosis of bladder	Tuberculosis	CALIBER
Read	A162.00	Tuberculosis of ureter	Tuberculosis	CALIBER
Read	A163.00	Tuberculosis of other urinary organs	Tuberculosis	CALIBER
Read	A164.00	Tuberculosis of epididymis	Tuberculosis	CALIBER
Read	A165000	Tuberculosis of prostate	Tuberculosis	CALIBER
Read	A165100	Tuberculosis seminal vesicle	Tuberculosis	CALIBER
Read	A165200	Tuberculosis of testis	Tuberculosis	CALIBER
Read	A165.00	Tuberculosis of other male genital organs	Tuberculosis	CALIBER
Read	A165z00	Tuberculosis of other male genital organs NOS	Tuberculosis	CALIBER
Read	A166000	Tuberculous oophoritis	Tuberculosis	CALIBER
Read	A166100	Tuberculous salpingitis	Tuberculosis	CALIBER
Read	A166111	Fallopian tube tuberculosis	Tuberculosis	CALIBER
Read	A166.00	Tuberculous oophoritis or salpingitis	Tuberculosis	CALIBER
Read	A166z00	Tuberculous oophoritis or salpingitis NOS	Tuberculosis	CALIBER
Read	A167000	Tuberculous cervicitis	Tuberculosis	CALIBER
Read	A167100	Tuberculous endometritis	Tuberculosis	CALIBER
Read	A167.00	Tuberculosis of other female genital organs	Tuberculosis	CALIBER
Read	A167z00	Tuberculosis of other female genital organs NOS	Tuberculosis	CALIBER
Read	A168.00	Tuberculosis of urinary tract	Tuberculosis	CALIBER
Read	A16..00	Tuberculosis of genitourinary system	Tuberculosis	CALIBER
Read	A16z.00	Genitourinary tuberculosis NOS	Tuberculosis	CALIBER
Read	A170000	Tuberculosis - lupus exedens	Tuberculosis	CALIBER
Read	A170100	Tuberculosis - lupus vulgaris	Tuberculosis	CALIBER
Read	A170200	Tuberculosis - scrofuloderma	Tuberculosis	CALIBER

Read	A170300	Tuberculosis - lupus NOS	Tuberculosis	CALIBER
Read	A170500	Tuberculosis cutis	Tuberculosis	CALIBER
Read	A170600	Tuberculosis lichenoides	Tuberculosis	CALIBER
Read	A170700	Tuberculosis papulonecrotica	Tuberculosis	CALIBER
Read	A170800	Tuberculosis verrucosa cutis	Tuberculosis	CALIBER
Read	A170.00	Tuberculosis of skin and subcutaneous tissue	Tuberculosis	CALIBER
Read	A170.11	Lupus - tuberculous	Tuberculosis	CALIBER
Read	A170z00	Tuberculosis of skin and subcutaneous tissue NOS	Tuberculosis	CALIBER
Read	A171000	Bazin's disease - erythema induratum - TB hypersensitivity	Tuberculosis	CALIBER
Read	A171100	Tuberculous erythema nodosum	Tuberculosis	CALIBER
Read	A171.00	Tuberculosis with erythema nodosum hypersensitivity reaction	Tuberculosis	CALIBER
Read	A171z00	Erythema nodosum with tuberculosis NOS	Tuberculosis	CALIBER
Read	A172000	Tuberculous - cervical lymphadenitis	Tuberculosis	CALIBER
Read	A172011	Scrofula - tuberculous cervical lymph nodes	Tuberculosis	CALIBER
Read	A172100	Scrofulous tuberculous abscess	Tuberculosis	CALIBER
Read	A172200	Tuberculous adenitis	Tuberculosis	CALIBER
Read	A172.00	Tuberculosis of peripheral lymph nodes	Tuberculosis	CALIBER
Read	A172z00	Tuberculosis of peripheral lymph nodes NOS	Tuberculosis	CALIBER
Read	A173000	Tuberculous chorioretinitis	Tuberculosis	CALIBER
Read	A173100	Tuberculous episcleritis	Tuberculosis	CALIBER
Read	A173200	Tuberculous interstitial keratitis	Tuberculosis	CALIBER
Read	A173300	Tuberculous chronic iridocyclitis	Tuberculosis	CALIBER
Read	A173400	Tuberculous keratoconjunctivitis	Tuberculosis	CALIBER
Read	A173.00	Tuberculosis of eye	Tuberculosis	CALIBER
Read	A173z00	Tuberculosis of eye NOS	Tuberculosis	CALIBER
Read	A174.00	Tuberculosis of ear	Tuberculosis	CALIBER
Read	A175.00	Tuberculosis of thyroid gland	Tuberculosis	CALIBER
Read	A176.00	Tuberculosis of adrenal glands - Addison's disease	Tuberculosis	CALIBER
Read	A177.00	Tuberculosis spleen	Tuberculosis	CALIBER
Read	A178.00	Tuberculosis oesophagus	Tuberculosis	CALIBER
Read	A17..00	Tuberculosis of other organs	Tuberculosis	CALIBER
Read	A17y000	Tuberculosis endocardium	Tuberculosis	CALIBER
Read	A17y100	Tuberculosis myocardium	Tuberculosis	CALIBER
Read	A17y200	Tuberculosis pericardium	Tuberculosis	CALIBER
Read	A17y300	Tuberculosis of stomach	Tuberculosis	CALIBER
Read	A17y400	Tuberculosis of liver	Tuberculosis	CALIBER
Read	A17y.00	Tuberculosis of other specified organs	Tuberculosis	CALIBER
Read	A17yz00	Tuberculosis of other specified organs NOS	Tuberculosis	CALIBER
Read	A17z.00	Tuberculosis of other organs NOS	Tuberculosis	CALIBER
Read	A180000	Acute miliary tuberculosis of a single specified site	Tuberculosis	CALIBER
Read	A180100	Acute miliary tuberculosis of multiple sites	Tuberculosis	CALIBER
Read	A180.00	Acute miliary tuberculosis	Tuberculosis	CALIBER

Read	A18..00	Miliary tuberculosis	Tuberculosis	CALIBER
Read	A18y.00	Other specified miliary tuberculosis	Tuberculosis	CALIBER
Read	A18z.00	Miliary tuberculosis NOS	Tuberculosis	CALIBER
Read	A1...00	Tuberculosis	Tuberculosis	CALIBER
Read	A1y..00	Other specified tuberculosis	Tuberculosis	CALIBER
Read	A1z..00	Tuberculosis NOS	Tuberculosis	CALIBER
Read	AE00.00	Late effects of respiratory tuberculosis	Tuberculosis	CALIBER
Read	AE01.00	Late effects of central nervous system tuberculosis	Tuberculosis	CALIBER
Read	AE02.00	Late effects of genitourinary system tuberculosis	Tuberculosis	CALIBER
Read	AE03.00	Late effects of tuberculosis of bones and joints	Tuberculosis	CALIBER
Read	AE04.00	Late effects of tuberculosis of other specified organs	Tuberculosis	CALIBER
Read	AE0..00	Late effects of tuberculosis	Tuberculosis	CALIBER
Read	AE0z.00	Late effects of tuberculosis NOS	Tuberculosis	CALIBER
Read	Ayu1000	[X]Other resp tubercul,confirmd bacteriologicly+histologicly	Tuberculosis	CALIBER
Read	Ayu1100	[X]Resp tuberculos unspcfd,confirmd bacteriolog+histologicly	Tuberculosis	CALIBER
Read	Ayu1300	[X]Resp TB unspcf,w'out mention/bacterial or histol confrmtn	Tuberculosis	CALIBER
Read	Ayu1400	[X]Other tuberculosis of nervous system	Tuberculosis	CALIBER
Read	Ayu1600	[X]Tuberculosis of other specified organs	Tuberculosis	CALIBER
Read	Ayu1800	[X]Other miliary tuberculosis	Tuberculosis	CALIBER
Read	Ayu1900	[X]Miliary tuberculosis, unspecified	Tuberculosis	CALIBER
Read	Ayu1.00	[X]Tuberculosis	Tuberculosis	CALIBER
Read	AyuJ200	[X]Sequelae of tuberculosis of bones and joints	Tuberculosis	CALIBER
Read	AyuJ400	[X]Sequelae of respiratory and unspecified tuberculosis	Tuberculosis	CALIBER
Read	F004.00	Meningitis - tuberculous	Tuberculosis	CALIBER
Read	F033300	Encephalitis due to tuberculosis	Tuberculosis	CALIBER
Read	F033311	Tuberculous encephalitis	Tuberculosis	CALIBER
Read	F040600	Tuberculous intracranial abscess	Tuberculosis	CALIBER
Read	F041300	Tuberculous intraspinal abscess	Tuberculosis	CALIBER
Read	F4A5500	Keratitis due to tuberculosis	Tuberculosis	CALIBER
Read	G500300	Acute pericarditis - tuberculous	Tuberculosis	CALIBER
Read	G500311	TB - acute pericarditis	Tuberculosis	CALIBER
Read	G520600	Acute myocarditis - tuberculous	Tuberculosis	CALIBER
Read	H450.00	Pneumoconiosis associated with tuberculosis	Tuberculosis	CALIBER
Read	J550200	Peritonitis - tuberculous	Tuberculosis	CALIBER
Read	K154800	Cystitis in tuberculosis	Tuberculosis	CALIBER
Read	K214300	Prostatitis in tuberculosis	Tuberculosis	CALIBER

Read	K43..00	Female tuberculous pelvic inflammatory disease	Tuberculosis	CALIBER
Read	N018.00	Tuberculous arthritis	Tuberculosis	CALIBER
Read	N304000	Tuberculosis of cervical spine	Tuberculosis	CALIBER
Read	N304100	Tuberculosis of thoracic spine	Tuberculosis	CALIBER
Read	N304200	Tuberculosis of lumbar spine	Tuberculosis	CALIBER
Read	N304300	Tuberculosis of sacrum/coccyx	Tuberculosis	CALIBER
Read	N304.00	Tuberculosis of spine (Pott's)	Tuberculosis	CALIBER
Read	N304.11	Tuberculosis of spine	Tuberculosis	CALIBER
Read	N305000	Tuberculosis of unspecified limb bone	Tuberculosis	CALIBER
Read	N305100	Tuberculosis of the upper arm bone	Tuberculosis	CALIBER
Read	N305200	Tuberculosis of the forearm bone	Tuberculosis	CALIBER
Read	N305300	Tuberculosis of the pelvic and thigh bones	Tuberculosis	CALIBER
Read	N305400	Tuberculosis of the lower leg bone	Tuberculosis	CALIBER
Read	N305500	Tuberculosis of other limb bones	Tuberculosis	CALIBER
Read	N305.00	Tuberculosis of limb bones	Tuberculosis	CALIBER
Read	N306000	Tuberculosis of bone, site unspecified	Tuberculosis	CALIBER
Read	N306100	Tuberculosis of the bones of the shoulder region	Tuberculosis	CALIBER
Read	N306200	Tuberculosis of the bones of the hand	Tuberculosis	CALIBER
Read	N306300	Tuberculosis of the bones of the ankle and foot	Tuberculosis	CALIBER
Read	N306400	Tuberculosis of the bones of other sites	Tuberculosis	CALIBER
Read	N306500	Tuberculosis of the bones of multiple sites	Tuberculosis	CALIBER
Read	N306.00	Tuberculosis of other bones	Tuberculosis	CALIBER
Read	N306z00	Tuberculosis of bone NOS	Tuberculosis	CALIBER
Read	Q402400	Congenital tuberculosis	Tuberculosis	CALIBER
Read	ZV12A00	[V] Personal history of pulmonary tuberculosis	Tuberculosis	CALIBER
Read	ZV12B00	[V] Personal history of tuberculosis	Tuberculosis	CALIBER
ICD10	A15	Respiratory tuberculosis, bacteriologically and histologically confirmed	Tuberculosis	CALIBER
ICD10	A16	Respiratory tuberculosis, not confirmed bacteriologically or histologically	Tuberculosis	CALIBER
ICD10	A17	Tuberculosis of nervous system	Tuberculosis	CALIBER
ICD10	A18	Tuberculosis of other organs	Tuberculosis	CALIBER
ICD10	A19	Miliary tuberculosis	Tuberculosis	CALIBER
ICD10	B20.0	HIV disease resulting in mycobacterial infection	Tuberculosis	CALIBER
ICD10	B90	Sequelae of tuberculosis	Tuberculosis	CALIBER
ICD10	J65	Pneumoconiosis associated with tuberculosis	Tuberculosis	CALIBER
ICD10	K23.0	Tuberculous oesophagitis	Tuberculosis	CALIBER
ICD10	K67.3	Tuberculous peritonitis	Tuberculosis	CALIBER

ICD10	K93.0	Tuberculous disorders of intestines, peritoneum and mesenteric glands	Tuberculosis	CALIBER
ICD10	M01.1	Tuberculous arthritis	Tuberculosis	CALIBER
ICD10	M49.0	Tuberculosis of spine	Tuberculosis	CALIBER
ICD10	M90.0	Tuberculosis of bone	Tuberculosis	CALIBER
ICD10	N33.0	Tuberculous cystitis	Tuberculosis	CALIBER
ICD10	N74.0	Tuberculous infection of cervix uteri	Tuberculosis	CALIBER
ICD10	N74.1	Female tuberculous pelvic inflammatory disease	Tuberculosis	CALIBER
ICD10	P37.0	Congenital tuberculosis	Tuberculosis	CALIBER
Read	J410000	Ulcerative ileocolitis	Ulcerative colitis	CALIBER
Read	J410100	Ulcerative colitis	Ulcerative colitis	CALIBER
Read	J410200	Ulcerative rectosigmoiditis	Ulcerative colitis	CALIBER
Read	J410300	Ulcerative proctitis	Ulcerative colitis	CALIBER
Read	J410400	Exacerbation of ulcerative colitis	Ulcerative colitis	CALIBER
Read	J410.00	Ulcerative proctocolitis	Ulcerative colitis	CALIBER
Read	J410z00	Ulcerative proctocolitis NOS	Ulcerative colitis	CALIBER
Read	J411.00	Ulcerative (chronic) enterocolitis	Ulcerative colitis	CALIBER
Read	J412.00	Ulcerative (chronic) ileocolitis	Ulcerative colitis	CALIBER
Read	J413.00	Ulcerative pancolitis	Ulcerative colitis	CALIBER
Read	J41..00	Idiopathic proctocolitis	Ulcerative colitis	CALIBER
Read	J41..11	Mucous colitis and/or proctitis	Ulcerative colitis	CALIBER
Read	J41..12	Ulcerative colitis and/or proctitis	Ulcerative colitis	CALIBER
Read	J41y.00	Other idiopathic proctocolitis	Ulcerative colitis	CALIBER
Read	J41yz00	Other idiopathic proctocolitis NOS	Ulcerative colitis	CALIBER
Read	J41z.00	Idiopathic proctocolitis NOS	Ulcerative colitis	CALIBER
Read	Jyu4100	[X]Other ulcerative colitis	Ulcerative colitis	CALIBER
Read	N031000	Arthropathy in ulcerative colitis	Ulcerative colitis	CALIBER
Read	N045400	Juvenile arthritis in ulcerative colitis	Ulcerative colitis	CALIBER
ICD10	K51	Ulcerative colitis	Ulcerative colitis	CALIBER
Read	65FY.00	Herpes zoster vaccination	VZ Vaccine	Jain 2018
Read	65FY.11	Shingles vaccination	VZ Vaccine	Jain 2018
Read	65FY000	Herpes zoster vaccination given by other health care provide	VZ Vaccine	Jain 2018
Read	68Nv.00	No consent for herpes zoster vaccination	VZ Vaccine	Jain 2018
Read	8I2r.00	Herpes zoster vaccination contraindicated	VZ Vaccine	Jain 2018
Read	8IEl.00	Herpes zoster vaccination declined	VZ Vaccine	Jain 2018
Read	9Nig.00	Did not attend herpes zoster vaccination	VZ Vaccine	Jain 2018
Read	U60K600	[X]Herpes zoster vacc caus adverse effects therapeutic use	VZ Vaccine	Jain 2018
prodcode	47327	Zostavax vaccine powder and solvent for suspension for injection 0.65ml pre-filled syringes (sanofi pasteur MSD Ltd)	VZ Vaccine	Jain 2018

prodcode	48314	Shingles (Herpes Zoster) vaccine (live) powder and solvent for suspension for injection 0.65ml pre-filled syringes	VZ Vaccine	Jain 2018
immtype	88	Shingles	VZ Vaccine	Jain 2018
immtype	91	Shingles OHP	VZ Vaccine	Jain 2018
Read	E100.00	Simple schizophrenia	Severe mental illness	Kontopantelis 2015
Read	E100000	Unspecified schizophrenia	Severe mental illness	Kontopantelis 2015
Read	E100100	Subchronic schizophrenia	Severe mental illness	Kontopantelis 2015
Read	E100.11	Schizophrenia simplex	Severe mental illness	Kontopantelis 2015
Read	E100200	Chronic schizophrenic	Severe mental illness	Kontopantelis 2015
Read	E100300	Acute exacerbation of subchronic schizophrenia	Severe mental illness	Kontopantelis 2015
Read	E100400	Acute exacerbation of chronic schizophrenia	Severe mental illness	Kontopantelis 2015
Read	E100500	Schizophrenia in remission	Severe mental illness	Kontopantelis 2015
Read	E100z00	Simple schizophrenia NOS	Severe mental illness	Kontopantelis 2015
Read	E101.00	Hebephrenic schizophrenia	Severe mental illness	Kontopantelis 2015
Read	E101000	Unspecified hebephrenic schizophrenia	Severe mental illness	Kontopantelis 2015
Read	E101400	Acute exacerbation of chronic hebephrenic schizophrenia	Severe mental illness	Kontopantelis 2015
Read	E101500	Hebephrenic schizophrenia in remission	Severe mental illness	Kontopantelis 2015
Read	E101z00	Hebephrenic schizophrenia NOS	Severe mental illness	Kontopantelis 2015
Read	E102.00	Catatonic schizophrenia	Severe mental illness	Kontopantelis 2015
Read	E102000	Unspecified catatonic schizophrenia	Severe mental illness	Kontopantelis 2015
Read	E102100	Subchronic catatonic schizophrenia	Severe mental illness	Kontopantelis 2015
Read	E102z00	Catatonic schizophrenia NOS	Severe mental illness	Kontopantelis 2015
Read	E103.00	Paranoid schizophrenia	Severe mental illness	Kontopantelis 2015
Read	E103000	Unspecified paranoid schizophrenia	Severe mental illness	Kontopantelis 2015
Read	E103200	Chronic paranoid schizophrenia	Severe mental illness	Kontopantelis 2015
Read	E103300	Acute exacerbation of subchronic paranoid schizophrenia	Severe mental illness	Kontopantelis 2015
Read	E103400	Acute exacerbation of chronic paranoid schizophrenia	Severe mental illness	Kontopantelis 2015

Read	E103z00	Paranoid schizophrenia NOS	Severe mental illness	Kontopanteli s 2015
Read	E104.00	Acute schizophrenic episode	Severe mental illness	Kontopanteli s 2015
Read	E105.00	Latent schizophrenia	Severe mental illness	Kontopanteli s 2015
Read	E105000	Unspecified latent schizophrenia	Severe mental illness	Kontopanteli s 2015
Read	E105200	Chronic latent schizophrenia	Severe mental illness	Kontopanteli s 2015
Read	E105z00	Latent schizophrenia NOS	Severe mental illness	Kontopanteli s 2015
Read	E106.00	Residual schizophrenia	Severe mental illness	Kontopanteli s 2015
Read	E107.00	Schizo-affective schizophrenia	Severe mental illness	Kontopanteli s 2015
Read	E107000	Unspecified schizo-affective schizophrenia	Severe mental illness	Kontopanteli s 2015
Read	E107100	Subchronic schizo-affective schizophrenia	Severe mental illness	Kontopanteli s 2015
Read	E107.11	Cyclic schizophrenia	Severe mental illness	Kontopanteli s 2015
Read	E107200	Chronic schizo-affective schizophrenia	Severe mental illness	Kontopanteli s 2015
Read	E107300	Acute exacerbation subchronic schizo-affective schizophrenia	Severe mental illness	Kontopanteli s 2015
Read	E107400	Acute exacerbation of chronic schizo-affective schizophrenia	Severe mental illness	Kontopanteli s 2015
Read	E107z00	Schizo-affective schizophrenia NOS	Severe mental illness	Kontopanteli s 2015
Read	E10y.00	Other schizophrenia	Severe mental illness	Kontopanteli s 2015
Read	E10y000	Atypical schizophrenia	Severe mental illness	Kontopanteli s 2015
Read	E10y100	Coenesthopathic schizophrenia	Severe mental illness	Kontopanteli s 2015
Read	E10y.11	Cenesthopathic schizophrenia	Severe mental illness	Kontopanteli s 2015
Read	E10yz00	Other schizophrenia NOS	Severe mental illness	Kontopanteli s 2015
Read	E10z.00	Schizophrenia NOS	Severe mental illness	Kontopanteli s 2015
Read	E11..00	Affective psychoses	Severe mental illness	Kontopanteli s 2015
Read	E110.00	Manic disorder; single episode	Severe mental illness	Kontopanteli s 2015
Read	E110000	Single manic episode; unspecified	Severe mental illness	Kontopanteli s 2015
Read	E110100	Single manic episode; mild	Severe mental illness	Kontopanteli s 2015

Read	E110.11	Hypomanic psychoses	Severe mental illness	Kontopantelis 2015
Read	E110200	Single manic episode; moderate	Severe mental illness	Kontopantelis 2015
Read	E110300	Single manic episode; severe without mention of psychosis	Severe mental illness	Kontopantelis 2015
Read	E110400	Single manic episode; severe; with psychosis	Severe mental illness	Kontopantelis 2015
Read	E110600	Single manic episode in full remission	Severe mental illness	Kontopantelis 2015
Read	E110z00	Manic disorder; single episode NOS	Severe mental illness	Kontopantelis 2015
Read	E111.00	Recurrent manic episodes	Severe mental illness	Kontopantelis 2015
Read	E111000	Recurrent manic episodes; unspecified	Severe mental illness	Kontopantelis 2015
Read	E111100	Recurrent manic episodes; mild	Severe mental illness	Kontopantelis 2015
Read	E11..12	Depressive psychoses	Severe mental illness	Kontopantelis 2015
Read	E111200	Recurrent manic episodes; moderate	Severe mental illness	Kontopantelis 2015
Read	E111300	Recurrent manic episodes; severe without mention psychosis	Severe mental illness	Kontopantelis 2015
Read	E111400	Recurrent manic episodes; severe; with psychosis	Severe mental illness	Kontopantelis 2015
Read	E111500	Recurrent manic episodes; partial or unspecified remission	Severe mental illness	Kontopantelis 2015
Read	E111z00	Recurrent manic episode NOS	Severe mental illness	Kontopantelis 2015
Read	E112400	Single major depressive episode; severe; with psychosis	Severe mental illness	Kontopantelis 2015
Read	E113400	Recurrent major depressive episodes; severe; with psychosis	Severe mental illness	Kontopantelis 2015
Read	E114.00	Bipolar affective disorder; currently manic	Severe mental illness	Kontopantelis 2015
Read	E114000	Bipolar affective disorder; currently manic; unspecified	Severe mental illness	Kontopantelis 2015
Read	E114100	Bipolar affective disorder; currently manic; mild	Severe mental illness	Kontopantelis 2015
Read	E114.11	Manic-depressive - now manic	Severe mental illness	Kontopantelis 2015
Read	E114200	Bipolar affective disorder; currently manic; moderate	Severe mental illness	Kontopantelis 2015
Read	E114300	Bipolar affect disord; currently manic; severe; no psychosis	Severe mental illness	Kontopantelis 2015
Read	E114400	Bipolar affect disord; currently manic;severe with psychosis	Severe mental illness	Kontopantelis 2015
Read	E114500	Bipolar affect disord;currently manic; part/unspec remission	Severe mental illness	Kontopantelis 2015

Read	E114z00	Bipolar affective disorder; currently manic; NOS	Severe mental illness	Kontopantelis 2015
Read	E115.00	Bipolar affective disorder; currently depressed	Severe mental illness	Kontopantelis 2015
Read	E115000	Bipolar affective disorder; currently depressed; unspecified	Severe mental illness	Kontopantelis 2015
Read	E115100	Bipolar affective disorder; currently depressed; mild	Severe mental illness	Kontopantelis 2015
Read	E115.11	Manic-depressive - now depressed	Severe mental illness	Kontopantelis 2015
Read	E115200	Bipolar affective disorder; currently depressed; moderate	Severe mental illness	Kontopantelis 2015
Read	E115300	Bipolar affect disord; now depressed; severe; no psychosis	Severe mental illness	Kontopantelis 2015
Read	E115400	Bipolar affect disord; now depressed; severe with psychosis	Severe mental illness	Kontopantelis 2015
Read	E115500	Bipolar affect disord; now depressed; part/unspec remission	Severe mental illness	Kontopantelis 2015
Read	E115z00	Bipolar affective disorder; currently depressed; NOS	Severe mental illness	Kontopantelis 2015
Read	E116.00	Mixed bipolar affective disorder	Severe mental illness	Kontopantelis 2015
Read	E116000	Mixed bipolar affective disorder; unspecified	Severe mental illness	Kontopantelis 2015
Read	E116100	Mixed bipolar affective disorder; mild	Severe mental illness	Kontopantelis 2015
Read	E116200	Mixed bipolar affective disorder; moderate	Severe mental illness	Kontopantelis 2015
Read	E116300	Mixed bipolar affective disorder; severe; without psychosis	Severe mental illness	Kontopantelis 2015
Read	E116400	Mixed bipolar affective disorder; severe; with psychosis	Severe mental illness	Kontopantelis 2015
Read	E116500	Mixed bipolar affective disorder; partial/unspec remission	Severe mental illness	Kontopantelis 2015
Read	E116z00	Mixed bipolar affective disorder; NOS	Severe mental illness	Kontopantelis 2015
Read	E117.00	Unspecified bipolar affective disorder	Severe mental illness	Kontopantelis 2015
Read	E117000	Unspecified bipolar affective disorder; unspecified	Severe mental illness	Kontopantelis 2015
Read	E117100	Unspecified bipolar affective disorder; mild	Severe mental illness	Kontopantelis 2015
Read	E117200	Unspecified bipolar affective disorder; moderate	Severe mental illness	Kontopantelis 2015
Read	E117300	Unspecified bipolar affective disorder; severe; no psychosis	Severe mental illness	Kontopantelis 2015
Read	E117400	Unspecified bipolar affective disorder;severe with psychosis	Severe mental illness	Kontopantelis 2015
Read	E117500	Unspecified bipolar affect disord; partial/unspec remission	Severe mental illness	Kontopantelis 2015

Read	E117z00	Unspecified bipolar affective disorder; NOS	Severe mental illness	Kontopantelis 2015
Read	E11y.00	Other and unspecified manic-depressive psychoses	Severe mental illness	Kontopantelis 2015
Read	E11y000	Unspecified manic-depressive psychoses	Severe mental illness	Kontopantelis 2015
Read	E11y100	Atypical manic disorder	Severe mental illness	Kontopantelis 2015
Read	E11y300	Other mixed manic-depressive psychoses	Severe mental illness	Kontopantelis 2015
Read	E11yz00	Other and unspecified manic-depressive psychoses NOS	Severe mental illness	Kontopantelis 2015
Read	E11z.00	Other and unspecified affective psychoses	Severe mental illness	Kontopantelis 2015
Read	E11z000	Unspecified affective psychoses NOS	Severe mental illness	Kontopantelis 2015
Read	E11zz00	Other affective psychosis NOS	Severe mental illness	Kontopantelis 2015
Read	E120.00	Simple paranoid state	Severe mental illness	Kontopantelis 2015
Read	E121.00	Chronic paranoid psychosis	Severe mental illness	Kontopantelis 2015
Read	E122.00	Paraphrenia	Severe mental illness	Kontopantelis 2015
Read	E123.00	Shared paranoid disorder	Severe mental illness	Kontopantelis 2015
Read	E123.11	Folie a deux	Severe mental illness	Kontopantelis 2015
Read	E12y.00	Other paranoid states	Severe mental illness	Kontopantelis 2015
Read	E12y000	Paranoia querulans	Severe mental illness	Kontopantelis 2015
Read	E12yz00	Other paranoid states NOS	Severe mental illness	Kontopantelis 2015
Read	E12z.00	Paranoid psychosis NOS	Severe mental illness	Kontopantelis 2015
Read	E13..00	Other nonorganic psychoses	Severe mental illness	Kontopantelis 2015
Read	E130.00	Reactive depressive psychosis	Severe mental illness	Kontopantelis 2015
Read	E130.11	Psychotic reactive depression	Severe mental illness	Kontopantelis 2015
Read	E131.00	Acute hysterical psychosis	Severe mental illness	Kontopantelis 2015
Read	E13..11	Reactive psychoses	Severe mental illness	Kontopantelis 2015
Read	E132.00	Reactive confusion	Severe mental illness	Kontopantelis 2015
Read	E133.00	Acute paranoid reaction	Severe mental illness	Kontopantelis 2015

Read	E133.11	Bouffee delirante	Severe mental illness	Kontopantelis 2015
Read	E134.00	Psychogenic paranoid psychosis	Severe mental illness	Kontopantelis 2015
Read	E13y.00	Other reactive psychoses	Severe mental illness	Kontopantelis 2015
Read	E13y000	Psychogenic stupor	Severe mental illness	Kontopantelis 2015
Read	E13y100	Brief reactive psychosis	Severe mental illness	Kontopantelis 2015
Read	E13yz00	Other reactive psychoses NOS	Severe mental illness	Kontopantelis 2015
Read	E13z.00	Nonorganic psychosis NOS	Severe mental illness	Kontopantelis 2015
Read	E13z.11	Psychotic episode NOS	Severe mental illness	Kontopantelis 2015
Read	E1z..00	Non-organic psychosis NOS	Severe mental illness	Kontopantelis 2015
Read	E212200	Schizotypal personality	Severe mental illness	Kontopantelis 2015
Read	Eu20.00	[X]Schizophrenia	Severe mental illness	Kontopantelis 2015
Read	Eu20000	[X]Paranoid schizophrenia	Severe mental illness	Kontopantelis 2015
Read	Eu20011	[X]Paraphrenic schizophrenia	Severe mental illness	Kontopantelis 2015
Read	Eu20100	[X]Hebephrenic schizophrenia	Severe mental illness	Kontopantelis 2015
Read	Eu20111	[X]Disorganised schizophrenia	Severe mental illness	Kontopantelis 2015
Read	Eu20200	[X]Catatonic schizophrenia	Severe mental illness	Kontopantelis 2015
Read	Eu20211	[X]Catatonic stupor	Severe mental illness	Kontopantelis 2015
Read	Eu20212	[X]Schizophrenic catalepsy	Severe mental illness	Kontopantelis 2015
Read	Eu20213	[X]Schizophrenic catatonia	Severe mental illness	Kontopantelis 2015
Read	Eu20214	[X]Schizophrenic flexibilatis cerea	Severe mental illness	Kontopantelis 2015
Read	Eu20300	[X]Undifferentiated schizophrenia	Severe mental illness	Kontopantelis 2015
Read	Eu20311	[X]Atypical schizophrenia	Severe mental illness	Kontopantelis 2015
Read	Eu20400	[X]Post-schizophrenic depression	Severe mental illness	Kontopantelis 2015
Read	Eu20500	[X]Residual schizophrenia	Severe mental illness	Kontopantelis 2015
Read	Eu20511	[X]Chronic undifferentiated schizophrenia	Severe mental illness	Kontopantelis 2015

Read	Eu20600	[X]Simple schizophrenia	Severe mental illness	Kontopantelis 2015
Read	Eu20y00	[X]Other schizophrenia	Severe mental illness	Kontopantelis 2015
Read	Eu20y12	[X]Schizophreniform disord NOS	Severe mental illness	Kontopantelis 2015
Read	Eu20y13	[X]Schizophrenifrm psychos NOS	Severe mental illness	Kontopantelis 2015
Read	Eu20z00	[X]Schizophrenia; unspecified	Severe mental illness	Kontopantelis 2015
Read	Eu21.00	[X]Schizotypal disorder	Severe mental illness	Kontopantelis 2015
Read	Eu21.11	[X]Latent schizophrenic reaction	Severe mental illness	Kontopantelis 2015
Read	Eu21.12	[X]Borderline schizophrenia	Severe mental illness	Kontopantelis 2015
Read	Eu21.13	[X]Latent schizophrenia	Severe mental illness	Kontopantelis 2015
Read	Eu21.14	[X]Prepsychotic schizophrenia	Severe mental illness	Kontopantelis 2015
Read	Eu21.15	[X]Prodromal schizophrenia	Severe mental illness	Kontopantelis 2015
Read	Eu21.16	[X]Pseudoneurotic schizophrenia	Severe mental illness	Kontopantelis 2015
Read	Eu21.17	[X]Pseudopsychopathic schizophrenia	Severe mental illness	Kontopantelis 2015
Read	Eu21.18	[X]Schizotypal personality disorder	Severe mental illness	Kontopantelis 2015
Read	Eu22.00	[X]Persistent delusional disorders	Severe mental illness	Kontopantelis 2015
Read	Eu22000	[X]Delusional disorder	Severe mental illness	Kontopantelis 2015
Read	Eu22011	[X]Paranoid psychosis	Severe mental illness	Kontopantelis 2015
Read	Eu22012	[X]Paranoid state	Severe mental illness	Kontopantelis 2015
Read	Eu22013	[X]Paraphrenia - late	Severe mental illness	Kontopantelis 2015
Read	Eu22014	[X]Sensitiver Beziehungswahn	Severe mental illness	Kontopantelis 2015
Read	Eu22015	[X]Paranoia	Severe mental illness	Kontopantelis 2015
Read	Eu22100	[X]Delusional misidentification syndrome	Severe mental illness	Kontopantelis 2015
Read	Eu22111	[X]Capgras syndrome	Severe mental illness	Kontopantelis 2015
Read	Eu22200	[X]Cotard syndrome	Severe mental illness	Kontopantelis 2015
Read	Eu22y00	[X]Other persistent delusional disorders	Severe mental illness	Kontopantelis 2015

Read	Eu22y11	[X]Delusional dysmorphophobia	Severe mental illness	Kontopantelis 2015
Read	Eu22y12	[X]Involutional paranoid state	Severe mental illness	Kontopantelis 2015
Read	Eu22y13	[X]Paranoia querulans	Severe mental illness	Kontopantelis 2015
Read	Eu22z00	[X]Persistent delusional disorder; unspecified	Severe mental illness	Kontopantelis 2015
Read	Eu23.00	[X]Acute and transient psychotic disorders	Severe mental illness	Kontopantelis 2015
Read	Eu23000	[X]Acute polymorphic psychot disord without symp of schizoph	Severe mental illness	Kontopantelis 2015
Read	Eu23011	[X]Bouffee delirante	Severe mental illness	Kontopantelis 2015
Read	Eu23012	[X]Cycloid psychosis	Severe mental illness	Kontopantelis 2015
Read	Eu23100	[X]Acute polymorphic psychot disord with symp of schizophren	Severe mental illness	Kontopantelis 2015
Read	Eu23112	[X]Cycloid psychosis with symptoms of schizophrenia	Severe mental illness	Kontopantelis 2015
Read	Eu23200	[X]Acute schizophrenia-like psychotic disorder	Severe mental illness	Kontopantelis 2015
Read	Eu23211	[X]Brief schizophreniform disorder	Severe mental illness	Kontopantelis 2015
Read	Eu23212	[X]Brief schizophrenifrm psych	Severe mental illness	Kontopantelis 2015
Read	Eu23214	[X]Schizophrenic reaction	Severe mental illness	Kontopantelis 2015
Read	Eu23300	[X]Other acute predominantly delusional psychotic disorders	Severe mental illness	Kontopantelis 2015
Read	Eu23312	[X]Psychogenic paranoid psychosis	Severe mental illness	Kontopantelis 2015
Read	Eu23y00	[X]Other acute and transient psychotic disorders	Severe mental illness	Kontopantelis 2015
Read	Eu23z00	[X]Acute and transient psychotic disorder; unspecified	Severe mental illness	Kontopantelis 2015
Read	Eu23z11	[X]Brief reactive psychosis NOS	Severe mental illness	Kontopantelis 2015
Read	Eu23z12	[X]Reactive psychosis	Severe mental illness	Kontopantelis 2015
Read	Eu24.00	[X]Induced delusional disorder	Severe mental illness	Kontopantelis 2015
Read	Eu24.12	[X]Induced paranoid disorder	Severe mental illness	Kontopantelis 2015
Read	Eu24.13	[X]Induced psychotic disorder	Severe mental illness	Kontopantelis 2015
Read	Eu25.00	[X]Schizoaffective disorders	Severe mental illness	Kontopantelis 2015
Read	Eu25000	[X]Schizoaffective disorder; manic type	Severe mental illness	Kontopantelis 2015

Read	Eu25011	[X]Schizoaffective psychosis; manic type	Severe mental illness	Kontopantelis 2015
Read	Eu25012	[X]Schizophreniform psychosis; manic type	Severe mental illness	Kontopantelis 2015
Read	Eu25100	[X]Schizoaffective disorder; depressive type	Severe mental illness	Kontopantelis 2015
Read	Eu25111	[X]Schizoaffective psychosis; depressive type	Severe mental illness	Kontopantelis 2015
Read	Eu25112	[X]Schizophreniform psychosis; depressive type	Severe mental illness	Kontopantelis 2015
Read	Eu25200	[X]Schizoaffective disorder; mixed type	Severe mental illness	Kontopantelis 2015
Read	Eu25212	[X]Mixed schizophrenic and affective psychosis	Severe mental illness	Kontopantelis 2015
Read	Eu25y00	[X]Other schizoaffective disorders	Severe mental illness	Kontopantelis 2015
Read	Eu25z00	[X]Schizoaffective disorder; unspecified	Severe mental illness	Kontopantelis 2015
Read	Eu25z11	[X]Schizoaffective psychosis NOS	Severe mental illness	Kontopantelis 2015
Read	Eu2y.00	[X]Other nonorganic psychotic disorders	Severe mental illness	Kontopantelis 2015
Read	Eu2y.11	[X]Chronic hallucinatory psychosis	Severe mental illness	Kontopantelis 2015
Read	Eu2z.00	[X]Unspecified nonorganic psychosis	Severe mental illness	Kontopantelis 2015
Read	Eu2z.11	[X]Psychosis NOS	Severe mental illness	Kontopantelis 2015
Read	Eu30.00	[X]Manic episode	Severe mental illness	Kontopantelis 2015
Read	Eu30000	[X]Hypomania	Severe mental illness	Kontopantelis 2015
Read	Eu30100	[X]Mania without psychotic symptoms	Severe mental illness	Kontopantelis 2015
Read	Eu30.11	[X]Bipolar disorder; single manic episode	Severe mental illness	Kontopantelis 2015
Read	Eu30200	[X]Mania with psychotic symptoms	Severe mental illness	Kontopantelis 2015
Read	Eu30211	[X]Mania with mood-congruent psychotic symptoms	Severe mental illness	Kontopantelis 2015
Read	Eu30212	[X]Mania with mood-incongruent psychotic symptoms	Severe mental illness	Kontopantelis 2015
Read	Eu30y00	[X]Other manic episodes	Severe mental illness	Kontopantelis 2015
Read	Eu30z00	[X]Manic episode; unspecified	Severe mental illness	Kontopantelis 2015
Read	Eu30z11	[X]Mania NOS	Severe mental illness	Kontopantelis 2015
Read	Eu31.00	[X]Bipolar affective disorder	Severe mental illness	Kontopantelis 2015

Read	Eu31000	[X]Bipolar affective disorder; current episode hypomanic	Severe mental illness	Kontopantelis 2015
Read	Eu31100	[X]Bipolar affect disorder cur epi manic wout psychotic symp	Severe mental illness	Kontopantelis 2015
Read	Eu31.11	[X]Manic-depressive illness	Severe mental illness	Kontopantelis 2015
Read	Eu31.12	[X]Manic-depressive psychosis	Severe mental illness	Kontopantelis 2015
Read	Eu31.13	[X]Manic-depressive reaction	Severe mental illness	Kontopantelis 2015
Read	Eu31200	[X]Bipolar affect disorder cur epi manic with psychotic symp	Severe mental illness	Kontopantelis 2015
Read	Eu31300	[X]Bipolar affect disorder cur epi mild or moderate depressn	Severe mental illness	Kontopantelis 2015
Read	Eu31400	[X]Bipol aff disord; curr epis sev depress; no psychot symp	Severe mental illness	Kontopantelis 2015
Read	Eu31500	[X]Bipolar affect dis cur epi severe depres with psyc symp	Severe mental illness	Kontopantelis 2015
Read	Eu31600	[X]Bipolar affective disorder; current episode mixed	Severe mental illness	Kontopantelis 2015
Read	Eu31800	[X]Bipolar affective disorder type I	Severe mental illness	Kontopantelis 2015
Read	Eu31900	[X]Bipolar affective disorder type II	Severe mental illness	Kontopantelis 2015
Read	Eu31911	[X]Bipolar II disorder	Severe mental illness	Kontopantelis 2015
Read	Eu31y00	[X]Other bipolar affective disorders	Severe mental illness	Kontopantelis 2015
Read	Eu31y11	[X]Bipolar II disorder	Severe mental illness	Kontopantelis 2015
Read	Eu31y12	[X]Recurrent manic episodes	Severe mental illness	Kontopantelis 2015
Read	Eu31z00	[X]Bipolar affective disorder; unspecified	Severe mental illness	Kontopantelis 2015
Read	Eu32300	[X]Severe depressive episode with psychotic symptoms	Severe mental illness	Kontopantelis 2015
Read	Eu32311	[X]Single episode of major depression and psychotic symptoms	Severe mental illness	Kontopantelis 2015
Read	Eu32312	[X]Single episode of psychogenic depressive psychosis	Severe mental illness	Kontopantelis 2015
Read	Eu32313	[X]Single episode of psychotic depression	Severe mental illness	Kontopantelis 2015
Read	Eu32314	[X]Single episode of reactive depressive psychosis	Severe mental illness	Kontopantelis 2015
Read	Eu32800	[X]Major depression; severe with psychotic symptoms	Severe mental illness	Kontopantelis 2015
Read	Eu33213	[X]Manic-depress psychosis;depressd;no psychotic symptoms	Severe mental illness	Kontopantelis 2015
Read	Eu33300	[X]Recurrent depress disorder cur epi severe with psyc symp	Severe mental illness	Kontopantelis 2015

Read	Eu33311	[X]Endogenous depression with psychotic symptoms	Severe mental illness	Kontopantelis 2015
Read	Eu33312	[X]Manic-depress psychosis;depressed type+psychotic symptoms	Severe mental illness	Kontopantelis 2015
Read	Eu33313	[X]Recurr severe episodes/major depression+psychotic symptom	Severe mental illness	Kontopantelis 2015
Read	Eu33314	[X]Recurr severe episodes/psychogenic depressive psychosis	Severe mental illness	Kontopantelis 2015
Read	Eu33315	[X]Recurrent severe episodes of psychotic depression	Severe mental illness	Kontopantelis 2015
Read	Eu33316	[X]Recurrent severe episodes/reactive depressive psychosis	Severe mental illness	Kontopantelis 2015
Read	Eu3z.11	[X]Affective psychosis NOS	Severe mental illness	Kontopantelis 2015
Read	Eu44.14	[X]Hysterical psychosis	Severe mental illness	Kontopantelis 2015
ICD10	F20	Schizophrenia	Severe mental illness	Kontopantelis 2015
ICD10	F21	Schizotypal disorder	Severe mental illness	Kontopantelis 2015
ICD10	F23	Acute and transient psychotic disorders	Severe mental illness	Kontopantelis 2015
ICD10	F24	Induced delusional disorder	Severe mental illness	Kontopantelis 2015
ICD10	F25	Schizoaffective disorders	Severe mental illness	Kontopantelis 2015
ICD10	F28	Other nonorganic psychotic disorders	Severe mental illness	Kontopantelis 2015
ICD10	F29	Unspecified nonorganic psychosis	Severe mental illness	Kontopantelis 2015
ICD10	F30	Manic episode	Severe mental illness	Kontopantelis 2015
ICD10	F31	Bipolar affective disorder	Severe mental illness	Kontopantelis 2015
ICD10	F32.2	Severe depressive episode without psychotic symptoms	Severe mental illness	Kontopantelis 2015
ICD10	F32.3	Severe depressive episode with psychotic symptoms	Severe mental illness	Kontopantelis 2015
ICD10	F33.2	Recurrent depressive disorder, current episode severe without psychotic symptoms	Severe mental illness	Kontopantelis 2015
ICD10	F33.3	Recurrent depressive disorder, current episode severe with psychotic symptoms	Severe mental illness	Kontopantelis 2015
Read	F390000	Benign congenital myopathy	Myopathy	Code Browser
Read	F390500	Congenital myopathy	Myopathy	Code Browser
Read	F390400	Nemaline body disease	Myopathy	Code Browser
Read	F390300	Myotubular myopathy	Myopathy	Code Browser

Read	F390200	Centronuclear myopathy	Myopathy	Code Browser
Read	F390100	Central core disease	Myopathy	Code Browser
Read	F39z.00	Myopathy or muscular dystrophy NOS	Myopathy	Code Browser
Read	F39B.00	Muscular dystrophy	Myopathy	Code Browser
Read	F391000	Duchenne muscular dystrophy	Myopathy	Code Browser
Read	F391800	Becker muscular dystrophy	Myopathy	Code Browser
Read	F391400	Facioscapulohumeral muscular dystrophy	Myopathy	Code Browser
Read	G558100	Cardiomyopathy in myotonic dystrophy	Myopathy	Code Browser
Read	F391100	Erb's muscular dystrophy	Myopathy	Code Browser
Read	F391z00	Hereditary progressive muscular dystrophy NOS	Myopathy	Code Browser
Read	F391500	Distal (Gower's) muscular dystrophy	Myopathy	Code Browser
Read	F391y00	Other specified hereditary progressive muscular dystrophy	Myopathy	Code Browser
Read	F390z00	Congenital hereditary muscular dystrophy NOS	Myopathy	Code Browser
Read	F391.00	Hereditary progressive muscular dystrophy	Myopathy	Code Browser
Read	F391200	Pelvic muscular dystrophy	Myopathy	Code Browser
Read	F391A00	Emery-Dreifuss muscular dystrophy	Myopathy	Code Browser
Read	F390.00	Congenital hereditary muscular dystrophy	Myopathy	Code Browser
Read	F391B00	Cardiomyopathy in Duchenne muscular dystrophy	Myopathy	Code Browser
Read	F391700	Oculopharyngeal muscular dystrophy	Myopathy	Code Browser
Read	F391600	Ocular muscular dystrophy	Myopathy	Code Browser
Read	F39X.00	Mitochondrial myopathy, not elsewhere classified	Myopathy	Code Browser
Read	Fyu8A00	[X]Mitochondrial myopathy, not elsewhere classified	Myopathy	Code Browser
Read	C315200	Kearns-Sayre syndrome	Myopathy	Code Browser
Read	C310311	Glycogenosis of liver and muscle	Myopathy	Code Browser
Read	C310313	Glycogenosis, type 3	Myopathy	Code Browser

Read	C310411	Glycogenosis, type 4	Myopathy	Code Browser
Read	C310113	Glycogenosis, type 2	Myopathy	Code Browser
Read	C310200	Hepatorenal glycogenosis	Myopathy	Code Browser
Read	C310.00	Glycogenosis - glycogen storage disease	Myopathy	Code Browser
Read	C310400	Glycogenosis with hepatic cirrhosis	Myopathy	Code Browser
Read	C310100	Generalised glycogenosis	Myopathy	Code Browser
Read	C310213	Glycogenosis, type 1	Myopathy	Code Browser
Read	C310.13	Glycogen storage disease	Myopathy	Code Browser
Read	C310z00	Glycogenosis NOS	Myopathy	Code Browser
Read	C310y00	Other specified glycogenosis	Myopathy	Code Browser
Read	C310012	Glycogenosis, type 5	Myopathy	Code Browser
Read	C310112	Pompe's disease	Myopathy	Code Browser
Read	C310412	Andersen's disease	Myopathy	Code Browser
Read	N003.00	Dermatomyositis	Myopathy	Code Browser
Read	N003000	Juvenile dermatomyositis	Myopathy	Code Browser
Read	Nyu4400	[X]Other dermatomyositis	Myopathy	Code Browser
Read	N003.11	Poikilodermatomyositis	Myopathy	Code Browser
Read	N231200	Traumatic myositis ossificans	Myopathy	Code Browser
Read	H57y100	Lung disease with polymyositis	Myopathy	Code Browser
Read	N241.00	Myalgia and myositis unspecified	Myopathy	Code Browser
Read	N003X00	Dermatopolymyositis, unspecified	Myopathy	Code Browser
Read	N241z00	Myalgia or myositis NOS	Myopathy	Code Browser
Read	N231400	Polymyositis ossificans	Myopathy	Code Browser
Read	N004.00	Polymyositis	Myopathy	Code Browser
Read	N003100	Dermatopolymyositis in neoplastic disease	Myopathy	Code Browser

Read	N231100	Progressive myositis ossificans	Myopathy	Code Browser
Read	Nyu4E00	[X]Dermatopolymyositis, unspecified	Myopathy	Code Browser
Read	R113.00	[D]Myoglobinuria	Myopathy	Code Browser
Read	C310000	McArdle's disease	Myopathy	Code Browser
Read	F393.00	Familial periodic paralysis	Myopathy	Code Browser
Read	F393.11	Familial hypokalaemic periodic paralysis	Myopathy	Code Browser
Read	F396z00	Symptomatic inflammatory myopathy in disease NOS	Myopathy	Code Browser
Read	F39W.00	Inflammatory myopathy, not elsewhere classified	Myopathy	Code Browser
Read	Fyu8B00	[X]Inflammatory myopathy, not elsewhere classified	Myopathy	Code Browser
Read	F396.00	Symptomatic inflammatory myopathy in disease EC	Myopathy	Code Browser
Read	F392400	Neuromyotonia	Myopathy	Code Browser
Read	F13z100	Stiff-man syndrome	Myopathy	Code Browser
Read	F13z111	Stiff person syndrome	Myopathy	Code Browser
ICD10	G71.2	Congenital myopathies	Myopathy	ICD10 browser
ICD10	G71.0	Muscular dystrophy	Myopathy	ICD10 browser
ICD10	G71.3	Mitochondrial myopathy, not elsewhere classified	Myopathy	ICD10 browser
ICD10	E74.0	Glycogen storage disease	Myopathy	ICD10 browser
ICD10	M33	Dermatopolymyositis	Myopathy	ICD10 browser
ICD10	M61.1	Myositis ossificans progressiva	Myopathy	ICD10 browser
ICD10	M61.2	Paralytic calcification and ossification of muscle	Myopathy	ICD10 browser
ICD10	R82.1	Myoglobulinuria	Myopathy	ICD10 browser
ICD10	G72.3	Periodic paralysis	Myopathy	ICD10 browser
ICD10	G72.4	Inflammatory myopathy, not elsewhere classified	Myopathy	ICD10 browser
ICD10	G71.1	Myotonic disorders	Myopathy	ICD10 browser
ICD10	G25.82	Stiff-man syndrome	Myopathy	ICD10 browser

ICD10	K57.0	Diverticulitis of small intestine with perforation and abscess	Diverticulitis	ICD10 browser
ICD10	K57.12	Diverticulitis of small intestine without perforation or abscess without bleeding	Diverticulitis	ICD10 browser
ICD10	K57.13	Diverticulitis of small intestine without perforation or abscess with bleeding	Diverticulitis	ICD10 browser
ICD10	K57.2	Diverticulitis of large intestine with perforation and abscess	Diverticulitis	ICD10 browser
ICD10	K57.32	Diverticulitis of large intestine without perforation or abscess without bleeding	Diverticulitis	ICD10 browser
ICD10	K57.33	Diverticulitis of large intestine without perforation or abscess with bleeding	Diverticulitis	ICD10 browser
ICD10	K57.4	Diverticulitis of both small and large intestines with perforation and abscess	Diverticulitis	ICD10 browser
ICD10	K57.52	Diverticulitis of both small and large intestines without perforation or abscess without bleeding	Diverticulitis	ICD10 browser
ICD10	K57.53	Diverticulitis of both small and large intestines without perforation or abscess with bleeding	Diverticulitis	ICD10 browser
ICD10	K57.8	Diverticulitis of intestine, part unspecified, with perforation and abscess	Diverticulitis	ICD10 browser
ICD10	K57.92	Diverticulitis of intestine, part unspecified, without perforation or abscess without bleeding	Diverticulitis	ICD10 browser
ICD10	K57.93	Diverticulitis of intestine, part unspecified, without perforation or abscess with bleeding	Diverticulitis	ICD10 browser
ICD10	E89.0	Postprocedural hypothyroidism	Hypothyroidism	ICD10 browser

References

CALIBER: Kuan V, Denaxas S, Gonzalez-Izquierdo A, Direk K, Bhatti O, Husain S, Sutaria S, Hingorani M, Nitsch D, Parisinos CA, Lumbers RT. A chronological map of 308 physical and mental health conditions from 4 million individuals in the English National Health Service. The Lancet Digital Health. 2019 Jun 1;1(2):e63-77.

Kontopantelis: Kontopantelis E, Olier I, Planner C, Reeves D, Ashcroft DM, Gask L, Doran T, Reilly S. Primary care consultation rates among people with and without severe mental illness: a UK cohort study using the Clinical Practice Research Datalink. BMJ open. 2015 Dec 1;5(12).

Jain: Jain A, Walker JL, Mathur R, Forbes HJ, Langan SM, Smeeth L, van Hoek AJ, Thomas SL. Zoster vaccination inequalities: A population based cohort study using linked data from the UK Clinical Practice Research Datalink. PloS one. 2018 Nov 15;13(11):e0207183.
