# Supplementary material for: Using electronic health records to inform trial feasibility in a rare autoimmune blistering skin disease in England
Source: BMC Med Res Methodol. 2021 Feb 4;21:22. doi: 10.1186/s12874-021-01212-1 (PMC7863423; doi:10.1186/s12874-021-01212-1)
Supplement: Supplementary file 2 — Additional file 2:. Identification of incident cases of bullous pemphigoid in HES-linked CPRD practices and HES admitted patient care in England 2015–2017 [file 12874_2021_1212_MOESM2_ESM.pdf]

Additional File 2

Figure. Identification of incident cases of bullous pemphigoid in HES-linked CPRD practices and HES admitted patient care in England between January 2015 and December 2017.

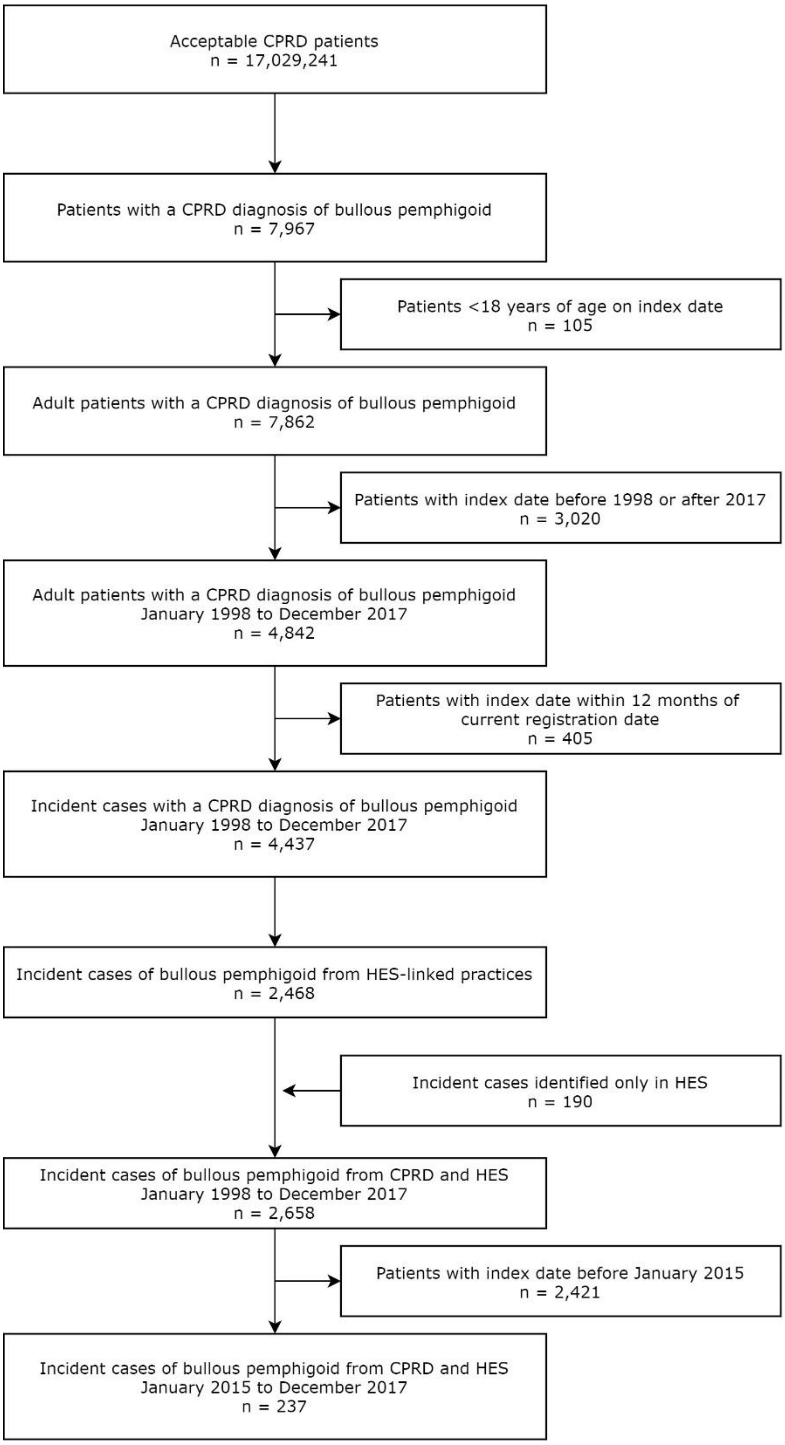

Identification of incident cases of bullous pemphigoid followed previously described methods,(1) where cases between 1998 and 2017 were identified. For the present study, only patients with an index date between 2015 and 2017 were included.

1. Persson MSM, Harman KE, Vinogradova Y, Langan SM, Hippisley-Cox J, Thomas KS, et al. Incidence, prevalence and mortality of bullous pemphigoid in England 1998–2017: a population-based cohort study. British Journal of Dermatology.n/a(n/a).
