# Supplementary material for: Using electronic health records to inform trial feasibility in a rare autoimmune blistering skin disease in England
Source: BMC Med Res Methodol. 2021 Feb 4;21:22. doi: 10.1186/s12874-021-01212-1 (PMC7863423; doi:10.1186/s12874-021-01212-1)
Supplement: Supplementary file 3 — Additional file 3:. Incidence rate of bullous pemphigoid per 100,000 person-years in England 2015–2017 by age category [file 12874_2021_1212_MOESM3_ESM.pdf]

Additional File 3

Table. Incidence rate of bullous pemphigoid per 100,000 person-years in England from 2015 to 2017 according to age category (years)

Age category	Incidence rate	Lower limit of 95% CI	Upper limit of 95% CI
18-19	1.36	0.19	9.63
20-24	0		
25-29	0.47	0.07	3.32
30-34	0.88	0.22	3.53
35-39	0.85	0.21	3.42
40-44	1.6	0.6	4.27
45-49	1.09	0.35	3.39
50-54	2.94	1.47	5.89
55-59	4.29	2.31	7.97
60-64	3.55	1.69	7.45
65-69	8.54	5.31	13.74
70-74	21.58	15.42	30.2
75-79	33.72	24.73	45.97
80-84	35.8	25.32	50.63
85-89	65.65	47.36	91.02
90+	124.23	90.76	170.03
